# Molecular mechanisms of transhydrogenase activity and allosteric regulation in eukaryotic type II PHGDH Ser33

**DOI:** 10.1038/s41467-026-74211-9

**Published:** 2026-06-19

**Authors:** Sebastian Perrone, Javier O. Cifuente, Leonardo Mastrella, Alberto Marina, Beatriz Trastoy, Julia Becker-Kettern, Jean-François Conrotte, Adrià Alcaide-Jiménez, Francisco Corzana, Enrico Glaab, Marcelo E. Guerin, Carole L. Linster

**Affiliations:** 1 Structural Glycobiology Laboratory, Biobizkaia Health Research Institute, Cruces University Hospital, Barakaldo, Bizkaia Spain; 2 Structural Glycobiology Laboratory, Center for Cooperative Research in Biosciences (CIC bioGUNE), Basque Research and Technology Alliance (BRTA), Bizkaia Technology Park, Derio, Spain; 3 Luxembourg Centre for Systems Biomedicine, University of Luxembourg, Esch-sur-Alzette, Luxembourg; 4 Instituto Biofisika (UPV/EHU, CSIC), University of the Basque Country, Leioa, Spain; 5 Structural Glycoimmunology Laboratory, Biobizkaia Health Research Institute, Cruces University Hospital, Barakaldo, Bizkaia Spain; 6 Ikerbasque, Basque Foundation for Science, Bilbao, Spain; 7 Structural Glycobiology Laboratory, Department of Structural and Molecular Biology; Molecular Biology Institute of Barcelona (IBMB), Spanish National Research Council (CSIC), Barcelona, Catalonia Spain; 8 Departamento Química and Instituto de Investigación en Química de la Universidad de La Rioja (IQUR) Universidad de La Rioja, Logroño, Spain

**Keywords:** X-ray crystallography, Enzyme mechanisms, Enzymes

## Abstract

L-serine is a critical structural constituent of proteins and membrane phospholipids, playing major roles in cell signaling, metabolism and development. L-Serine is synthesized through a conserved de novo pathway starting from the glycolytic intermediate 3-phosphoglycerate (PGA), being oxidized by 3-phosphoglycerate dehydrogenase (PHGDH) into 3-phosphohydroxypyruvate (PHP). In certain organisms, PHGDH operates as a transhydrogenase using α-ketoglutarate rather than NAD^+^ as the final electron acceptor and producing both PHP and D-2-hydroxyglutarate (2HG). We provide high-resolution X-ray crystal structures of the transhydrogenase Ser33 from *Saccharomyces cerevisiae*, in complex with the cofactor NADH, and with PGA, PHP, 2HG and the negative allosteric regulator L-serine. Combining extensive alanine scanning mutagenesis, enzyme activity assays and kinetics, molecular dynamics simulations, biophysical methods, and phylogenetic analysis, we establish the molecular basis of substrate recognition, transhydrogenase activity, and allosteric inhibition mechanisms, including the role of an N-terminal extension in the regulation of eukaryotic Type II PHGDHs.

## Abstract (web summary)

PHGDH plays a key role in the biosynthesis of L-serine. In this study, the authors elucidate the molecular mechanisms underlying substrate recognition, transhydrogenase activity, and allosteric inhibition in the eukaryotic Type II Ser33 enzyme.

## Introduction

L-serine plays a central role in metabolism, linking several biochemical pathways critical for the function of different types of cells and organisms^1^. In addition, L-serine is the building block of main structural components of the cell, in the form of proteins and membrane phospholipids^2^. L-serine is available from different sources, comprising (i) dietary intake, (ii) de novo synthesis from carbohydrates, (iii) regeneration from glycine and one carbon units, and (iv) recycling from protein/phospholipid degradation^1^. L-serine can be synthetized by conversion of glycine, in a reaction catalyzed by serine hydroxymethyltransferase (SHMT)^3^. L-serine and glycine are rapidly exchanged in reactions catalyzed by two protein isoforms of SHMT located in the cytosol (SHMT1) and mitochondria (SHMT2)^4^.

**Fig. 1:**
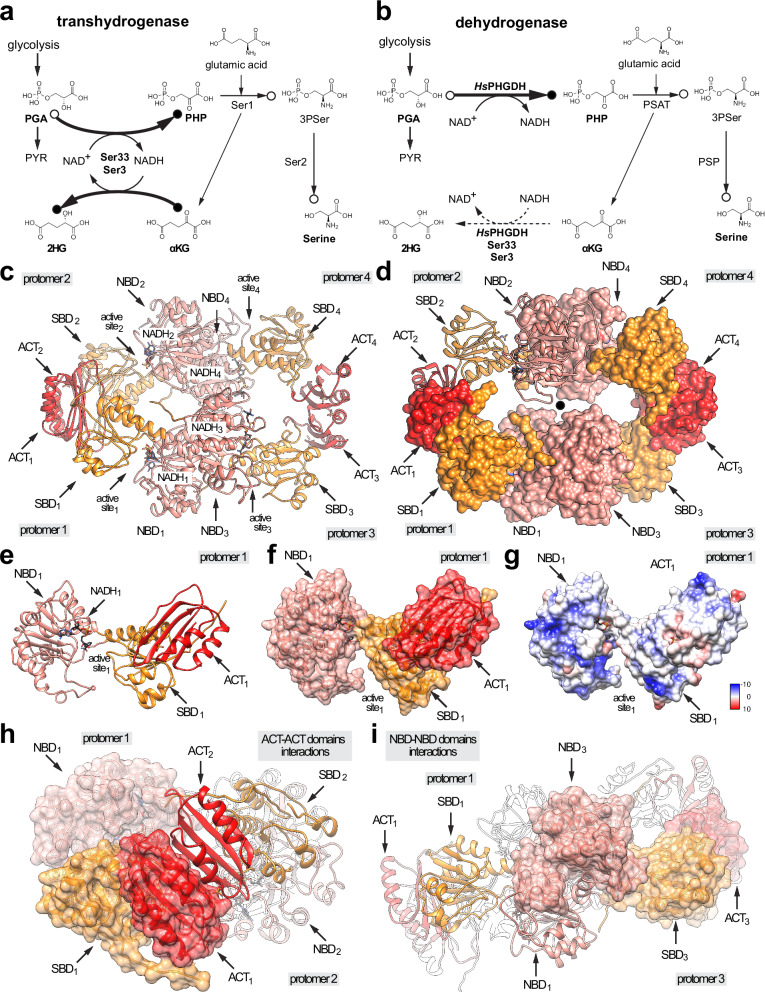
The Ser33 transhydrogenase from *S. cerevisiae.* **a**, **b** PHGDH dehydrogenase and transhydrogenase mechanisms. De novo L-serine synthesis involves three consecutive steps, (i) oxidation of 3PGA to PHP, (ii) transamination of PHP to 3PSer by Ser1 (yeast) or PSAT1 (humans), and (iii) dephosphorylation of 3PSer to serine by Ser2 (yeast) or PSPH (humans). In *S. cerevisiae*, the first step is catalyzed by two isozymes, Ser3 and Ser33, which carry out a 3PGA-αKG transhydrogenase reaction. The first half-reaction involves oxidation of 3PGA to PHP, accompanied by the reduction of the enzyme-bound NAD^+^ coenzyme to NADH. In the second half-reaction, αKG is reduced to D-2HG with concomitant regeneration of NAD^+^. In contrast, *Hs*PHGDH consumes external NAD^+^ for 3PGA oxidation and does not couple this reaction to αKG reduction. Both the Ser3/Ser33 and *Hs*PHGDH enzymes can, however, reduce αKG to 2HG in the presence of external NADH and absence of 3PGA. **c**, **d** The overall structure of the Ser33 homotetramer in complex with NADH. Three protomers shown in surface representation and one protomer shown in cartoon representation to visualize the secondary structural organization and the NADH. Each protomer of the homotetramer is composed of the NBD domain (salmon), the SBD domain (orange), and the ACT regulatory domain (red). The solid circle corresponds to a 2-fold axis. **e**–**g** The overall structure of the Ser33 protomer in complex with NADH shown in cartoon representation (**e**), surface representation (**f**), and electrostatic surface representation (**g**). **h** Close-up view of the ACT1-ACT2 interface. **i** Close-up view of the NBD1-NBD3 interface, and the active site cavity.

The de novo L-serine biosynthesis pathway comprises three sequential enzymatic steps: (i) the phosphoglycerate dehydrogenase PHGDH catalyzes the oxidation of the glycolytic intermediate 3-phosphoglycerate (PGA) to 3-phosphohydroxypyruvate (PHP), in the presence of NAD^+^ as a cofactor (Fig. 1a,b)^5–7^, (ii) the phosphoserine aminotransferase PSAT catalyzes the transamination of PHP to 3-phosphoserine (3PSer), using glutamate as amino donor, and (iii) the phosphoserine phosphatase PSP^8^ catalyzes the irreversible hydrolysis of 3PSer to L-serine and inorganic phosphate (Pi). It is worth noting that the reaction catalyzed by PHGDH is thermodynamically unfavored, and in vitro assays should be coupled with the PSAT reaction^9–12^. This metabolic pathway, including the PHGDH enzyme, is highly conserved across all kingdoms of life and has been extensively studied in diverse model organisms^8,13–16^.

PHGDHs are classified in three different groups: Type I, II and III, based on their overall architecture^7,17–24^. All types of PHGDHs contain two highly conserved domains forming the catalytic core: (i) the nucleotide binding domain (NBD) that binds NAD^+^ or NADH and (ii) the substrate binding domain (SBD). Both domains comprise Rossmann folds and are connected through a flexible hinge^18^, forming the active site^25^. Compared to other categories, Type I PHGDHs display two extra domains: (i) the allosteric substrate binding domain (ASB)^26^ and (ii) the regulatory aspartate kinase-chorismate mutase-tyrA domain (ACT). Type II PHGDHs consist of the catalytic core and the ACT domain, while Type III PHGDHs contain only the catalytic core^17,24,27^. PHGDHs also differ at their quaternary levels, with Type I and II enzymes being assembled as tetramers, while Type III PHGDHs form dimers^9,17,24,28^. Type III PHGDHs are further divided in two subtypes: (i) one displaying a conserved histidine as the catalytic residue (IIIH) and (ii) a second one where histidine is replaced by lysine (IIIK)^17^.

Type II PHGDHs have a distinct and intriguing catalytic ability that was discovered only recently. The oxidation of PGA into PHP is coupled with the concomitant reduction of α-ketoglutarate (αKG) into 2-hydroxyglutarate (2HG), generating a secondary product of the main reaction towards the L-Serine synthesis. Type II PHGDHs thus act as transhydrogenases, using NAD^+^ as an intermediary electron acceptor, rather than as dehydrogenases, classically using NAD^+^ as the final electron acceptor (Fig. 1a,b)^29^. This has been observed in Type II PHGDH enzymes from *Escherichia coli* (*Ec*PHGDH)^30^, a paradigmatic enzyme for prokaryotic Type II PHGDHs, *Pseudomonas stutzeri* (*Ps*PHGDH)^12^, and in two paralogous enzymes, Ser3 and Ser33, from *S. cerevisiae*^24,29^. The de novo L-serine biosynthetic pathway is critical for the physiology of *S. cerevisiae*, with deletion of *SER3* and *SER33* leading to a conditional L-serine auxotrophy during growth on glucose^29,31,32^.

2HG started raising attention when high levels of this dicarboxylic acid were found as a signature molecule in extracellular fluids of certain organic aciduria patients^33,34^. Interest rose even further when 2HG emerged as an oncometabolite, being produced in certain tumors carrying point mutations in the isocitrate dehydrogenase enzymes IDH1^35^ or IDH2^36^. 2HG is a chiral molecule and is now commonly considered as a non-canonical metabolite in mammals, L-2HG or D-2HG being stereospecifically produced, under physiological conditions, by slow undesired side reactions of certain metabolic enzymes, including an αKG reductase activity of human PHGDH^37,38^. Budding yeast, however, produces D-2HG^29^ as a canonical product of the transhydrogenase activity of Ser3 and Ser33 in de novo L-serine synthesis^24^, as described above. Unlike the yeast PHGDHs, human PHGDH (*Hs*PHGDH) behaves as a classical NAD-dependent dehydrogenase for PGA oxidation. This is also the case for other Type I enzymes, including PHGDHs from rat (*r*PHGDH)^6^ and the paradigmatic prokaryotic Type I PHGDH from *Mycobacterium tuberculosis* (*Mt*PHGDH)^21,22^, and for Type III enzymes, such as the PHGDH from *Entamoeba histolytica* (*Eh*PHGDH)^27^. Intriguingly, type II PHGDHs, although they cannot act as dehydrogenases for PGA oxidation, can present a robust NADH-dependent αKG reductase activity—producing D-2HG in the absence of PGA—, as shown for instance for the yeast Ser3 and Ser33 enzymes^29^. Heterologous expression of *Hs*PHGDH cannot rescue growth of a yeast *ser3*Δ*ser33*Δ double mutant strain on a fermentative carbon source^24^, suggesting that the transhydrogenase mechanism — there is no net consumption of NAD^+^—might represent an evolutionary advantage for certain organisms by rendering L-serine synthesis more independent of the NAD^+^:NADH ratio. This may be particularly important for organisms, such as *S. cerevisiae*, that present a generally lower intracellular NAD^+^:NADH ratio than mammalian cells, which would not push the dehydrogenase reaction in the physiological direction^24^. In fact, the coupling of PGA oxidation with reduction of αKG into 2HG in the transhydrogenase reaction confers a thermodynamic advantage by lowering the Gibbs free energy as compared to the dehydrogenase reaction^12^. Finally, it is worth noting that PSAT, the enzyme involved in the second committed step of the Ichihara-Greenberg pathway, produces 3PSer from PHP with concomitant conversion of glutamic acid into αKG, which can in turn push the transhydrogenase reaction towards PHP formation in the first step.

Ser3 and Ser33, as well as other Type II PHGDHs, including prokaryotic *Ec*PHGDH, are allosterically inhibited by L-serine^23,24^, thereby conferring negative feedback regulation to the serine synthesis pathway by its end product. Type I prokaryotic *Mtb*PHGDH is also inhibited by L-serine^22^. Structural work on prokaryotic Type I and Type II PHGDHs has provided insights into catalytic domain organization and tetrameric assembly. The structures of *Ec*PHGDH and *Mtb*PHGDH in complex with L-serine show a pronounced conformational heterogeneity and asymmetry within subunits^18,39^. These studies proposed a flexible hinge region linking the regulatory (ACT) and catalytic domains to modulate enzymatic activity^40^. Intriguingly, mammalian (type I) PHGDHs—e.g., *Hs*PHGDH^9^ and *r*PHGDH^6^—, which also contain the ACT domain, are not allosterically regulated by L-serine. Type III enzymes lack the regulatory ACT domain and are not inhibited by L-serine either.

Structural work on eukaryotic PHGDHs has largely focused on Type I human PHGDH in the context of cancer metabolism and inhibitory mechanisms^41–46^. While these studies identified ligand- and inhibitor-binding sites and enabled the progress of structure-based drug discovery strategies, available structures represent only partial constructs or inhibitor-bound states and provide limited insight into intrinsic catalytic or regulatory mechanisms. Across PHGDH classes, and despite the substantial number of deposited structures, key fundamental questions remain unresolved. These include how conserved active-site residues orchestrate hydride transfer and substrate oxidation, how cofactor and substrate binding are coordinated at the atomic level, and how local catalytic events are coupled to conformational rearrangements within the homotetramer. Importantly, no structural analysis has so far been performed for any eukaryotic Type II PHGDH, whose catalytic mechanism is known to markedly differ from Type I PHGDHs.

In this work, we provide high-resolution X-ray crystal structures of Ser33 from *S. cerevisiae* in the presence of the cofactor NADH, and in complex with NADH-PGA, NADH-PHP, and NADH-2HG, as well as the complex with NADH and the negative allosteric regulator L-serine. Using a multidisciplinary approach, we present a comprehensive model supporting the molecular basis of substrate recognition, transhydrogenase activity, and allosteric inhibition mechanisms for Ser33. In addition, our work enables direct comparisons—based on the current available experimental data—with both Type II prokaryotic and Type I PHGDHs, revealing conserved and divergent mechanistic features, and markedly advancing our understanding of PHGDH catalysis and regulation, particularly in the eukaryotic context.

## Results

### The overall architecture of Ser33

The crystal structure of full-length Ser33 (469 residues; UniProt code P40510) was determined at 3.2 Å resolution using molecular replacement methods (Ser33-NADH hereafter; PDB code 8PIN; Supplementary Table 1; Supplementary Fig. 1a; the structural model used for molecular replacement corresponds to the PDB code 1YBA; see Methods section). The high quality of the electron density maps allowed the trace of residues 46–469 (Supplementary Figs. 2-8). Ser33 crystallized as an oval ring-shaped homotetramer, with each protomer comprising three domains, from N- to C-terminus: (i) the substrate binding domain (SBD; residues 57–155 and 353–386), (ii) the nucleotide binding domain (NBD; residues 156–352) and (iii) the aspartate kinase-chorismate mutase-TyrA domain (ACT; residues 387–469; Fig. 1c-i).

The SBD consists of one Rossmann fold domain^18,25^. The core is composed of a central β-sheet comprising five β-strands with topology β5, β4, β3, β1, and β2, flanked on both sides with several α-helices, and an overall size of 45.1 Å × 37.6 Å × 34.0 Å (Fig. 1e). The NBD domain consists of a second Rossmann fold domain. The core is composed of a central β-sheet comprising six β-strands with topology β7, β6, β8, β9, β10 and β11, flanked on both sides with several α-helices, and an overall size of 54.6 Å × 45.7 Å × 39.8 Å (Fig. 1e). An NADH molecule is associated to all four NBD domains of the Ser33 homotetramer, although the cofactor was not supplemented in the crystallization experiments. The ACT domain consists of a central β-sheet comprising four β-strands with topology β13, β14, β12 and β15, of which β13 and β15 are antiparallel, flanked with two α-helices, and an overall size of 41.7 Å × 37.9 Å × 27.6 Å (Fig. 1e). The SBD domain connects with both NBD and ACT domains of the same protomer. Specifically, the SBD–ACT interaction is mainly mediated by (i) the connecting loop α12–β12 (named regulatory loop ‘RL’ hereafter; residues 373–397), (ii) α4, β4 and the connecting loop α4-β4 from SBD, and (iii) β12, β14 and β15 from ACT. The SBD–NBD interaction is mainly mediated by the connecting loops β4–α5 (residues 150-155) and β11–α12 (residues 345–352).

The Ser33 protomers (51.19 kDa) build into a physiological and functional homotetrameric structure (204.76 kDa), which was further confirmed by size exclusion chromatography (Supplementary Fig. 1b,c) and has an overall size of ca. 121.0 Å × 86.7 Å × 45.7 Å. This quaternary architecture is assembled primarily by NBD–NBD and ACT–ACT interactions of different protomers (Fig. 1c,d). Specifically, the NBD–NBD”’ domains associate by an extensive interaction surface mainly mediated by (i) α5 and α5”’, (ii) α6 and the connecting loop α6–β13 (named ‘WL’ loop hereafter, residues 183-199) with α11”’, β11”’, and the connecting loops β10”’–α11”’ and β11”’–α12”’. Interestingly, the highly conserved Trp186 present in the WL is deeply buried into a hydrophobic pocket of the neighbor NBD domain (Fig. 1i). The ACT–ACT” interaction is mainly mediated by α13 and β13 from ACT with the equivalent secondary structural elements α13” and β13” in ACT” (Fig. 1h). As a consequence, ACT and ACT” form a common β-sheet composed of 8 strands (Fig. 1h).

### The active site and catalytic mechanism of Ser33

**Fig. 2:**
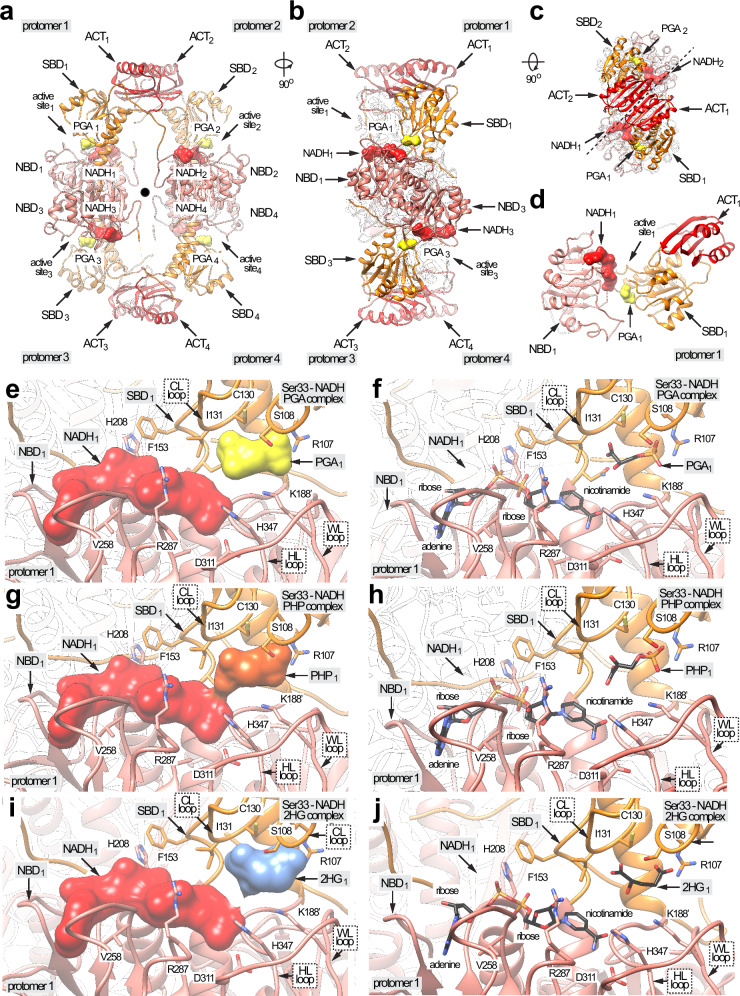
The active site of Ser33. **a**–**c** The overall structure of the Ser33 homotetramer in complex with NADH and PGA. All protomers are shown in cartoon representation, including the NBD domains (salmon), the SBD domains (orange), and the ACT regulatory domains (red). The NADH and PGA ligands are shown in surface representation (red and yellow, respectively). The solid circle corresponds to a 2-fold axis. **d** Close-up view of the Ser33 protomer active site, showing the location of NADH and PGA. **e**, **f** Close-up view of the Ser33 active site in the Ser33-NADH-PGA complex (PDB code 8PIR), showing the location of NADH (red) and PGA (yellow). **g**, **h** Close-up view of the Ser33 active site in the Ser33-NADH-PHP complex (PDB code 8PIO), showing the location of NADH (red) and PHP (orange). **i**, **j** Close-up view of the Ser33 active site in the Ser33-NADH-2HG-SER complex (PDB code 8Q2I), showing the location of NADH (red) and 2HG (blue).

To understand the molecular basis of the transhydrogenase activity, we further solved the crystal structures of Ser33 in the presence of (i) PGA (Ser33-NADH-PGA hereafter; PDB code 8PIR), (ii) PHP (Ser33-NADH-PHP hereafter; PDB code 8PIO), and (iii) 2HG and serine (Ser33-NADH-2HG-SER hereafter; PDB code 8Q2I), at 2.8 Å, 2.7 Å and 2.5 Å resolution, respectively (Figs. 1 and 2; Supplementary Table 1; for a full list of Ser33-ligand/s contacts, please refer to Supplementary Figs. 4-8; Methods section). The active site of Ser33 is located in a deep fissure between the SBD and NBD domains (Figs. 1 and 2). A long, deep, open groove located at the top of the central β-sheet of the NBD contains the binding site for the NADH cofactor (Figs. 1 and 2). The groove is flanked by the connecting loops β7–α8 (residues 227–243), β6–α7 (residues 204–207), β8–α9 (residues 257–269), β9–α10 (residues 285–292), β10–α11 (residues 311–332), β11–α12 (residues 345–352), and β5–α5 (residues 150–155; Fig. 2e-j). The adenine moiety accommodates into a cavity flanked by the connecting loops β6–α7, β7–α8 and β8–α9. The O2 and O3 atoms of the ribose ring attached to the heterocycle make hydrogen bonds with the side chain of D228. The pyrophosphate group is placed at the N-terminal end of α7, with the PO_4_ group attached to adenosine, making a strong electrostatic interaction with the side and main chains of H208. The side chain of H208 also makes an important stacking interaction with the side chain of F153, markedly contributing to the association of the SBD-NBD domains, and a hydrogen bond with the side chain of E316 (Fig. 2). The PO_4_ group attached to the nicotinamide ribose also makes electrostatic interactions with the main chains of H208 and I209. The O2 atom of the ribose ring attached to nicotinamide makes electrostatic interactions with the guanidinium group of R287, while the O3 atom makes a hydrogen bond with the main chain of V258. The carbonyl group of nicotinamide makes a hydrogen bond with the main chain of G349 and an electrostatic interaction with the side chain of H347. The amide group nitrogen makes a hydrogen bond with the main chain of A285 and the side chain of D311 (Fig. 2).

PHGDHs catalyze the first committed step in the Ichihara Greenberg pathway that leads to L-serine synthesis, namely the oxidation of PGA into PHP with the concomitant reduction of NAD^+^ into NADH (Fig. 1a,b; Supplementary Fig. 9a,b). This reaction is considered a classical dehydrogenase reaction; however, Type II PHGDHs, including Ser33, have a distinctive and intriguing catalytic property. The oxidation of PGA into PHP is coupled with the reduction of αKG into 2HG, which is thus formed in stoichiometric amounts with PHP en route towards L-serine synthesis. Ser33 acts therefore as a transhydrogenase enzyme rather than a classical dehydrogenase, which uses NAD^+^ as the final electron acceptor (Fig. 1a,b; Supplementary Fig. 9a,b).

The current accepted mechanism for the classical dehydrogenase reaction catalyzed by PHGDHs involves two steps: (i) the catalytic histidine (H347 in Ser33), located in the Catalytic Loop (named ‘HL’ loop hereafter; residues 345–352) attracts a proton from the hydroxyl group in βC of the PGA substrate, facilitated by a glutamate residue (E316 in Ser33). A hydride from the βC of PGA is transferred to the positively charged nicotinamide ring of NAD^+^, in an aromatic bimolecular nucleophilic addition reaction; (ii) H347 is deprotonated by a hydroxide ion derived from water, regenerating the enzyme and releasing the products, NADH and PHP (Supplementary Fig. 9a,b). The PGA substrate is located at the interface between the SBD and NBD domains (Ser33-NADH-PGA; Fig. 2e,f). Specifically, PGA mainly binds to a small pocket flanked by the connecting loops β3–α3 (residues 107–113) and β4–α4 (named ‘CL’ loop hereafter; residues 129–137). The carboxylate group of PGA makes hydrogen bonds with the main chains of I131 and G132. The PO_4_ moiety makes hydrogen bonds with the main chain of S108 and the guanidinium group of R107 from the SBD, and the side chain of K188”’ from the NBD”’ neighbor protomer. The PHP product is located in the same pocket as PGA (Ser33-NADH-PHP; Fig. 2g,h). The carbonyl group of PHP is mostly oriented towards the CL loop, and the βC keto group making a hydrogen bond with the ε-ammonium group of K188”’. The PO_4_ moiety makes hydrogen bonds with the main chain of S108 and the ε-ammonium atom of R107 from the SBD, and the side chain of K188”’ from the NBD”’. This residue seems to play a key role, interacting with the keto/hydroxyl groups of PHP and PGA/2HG, respectively, orienting not only the substrates/products, but also the phosphate group. The side chains of residues N155 and Q356, located in α5 and α15, respectively, also contribute to the appropriate arrangement of PGA, PHP and 2HG in the active site. The catalytic H347 lies in a middle point between the nicotinamide ring of the cofactor and the βC of PGA and PHP. Interestingly, C130, located in the CXC signature motif conserved in Type II PHGDHs (C128 and C130 in Ser33) seems to also play a role in the proper orientation of PGA and PHP, in the active site. A key difference between the coordination of PGA and PHP is that, in the oxidation reaction, the hybridization of the βC turns from sp^3^ to sp^2^, rearranging the symmetry of the carbon from a distorted tetrahedral to a trigonal plane.

The 2HG product is located in the same pocket as PGA and PHP (Ser33-NADH-2HG-SER; Fig. 2i,j). 2HG does not induce notable conformational changes at the substrate binding site. The lactate moiety of 2HG is positioned towards the nicotinamide group of NADH, in close contact with the CL, HL and WL loops, while the acetate group interacts with the connecting loops β3–α3. Specifically, the C1 carboxyl of 2HG makes a hydrogen bond with the main chain of I131. The C2 hydroxyl of 2HG forms a hydrogen bond with the ε-ammonium group of K188”’, whereas the C5-carboxyl makes hydrogen bonds with the side chain of S108 and the ε-ammonium of R107.

### The transhydrogenase mechanism of Ser33

**Fig. 3:**
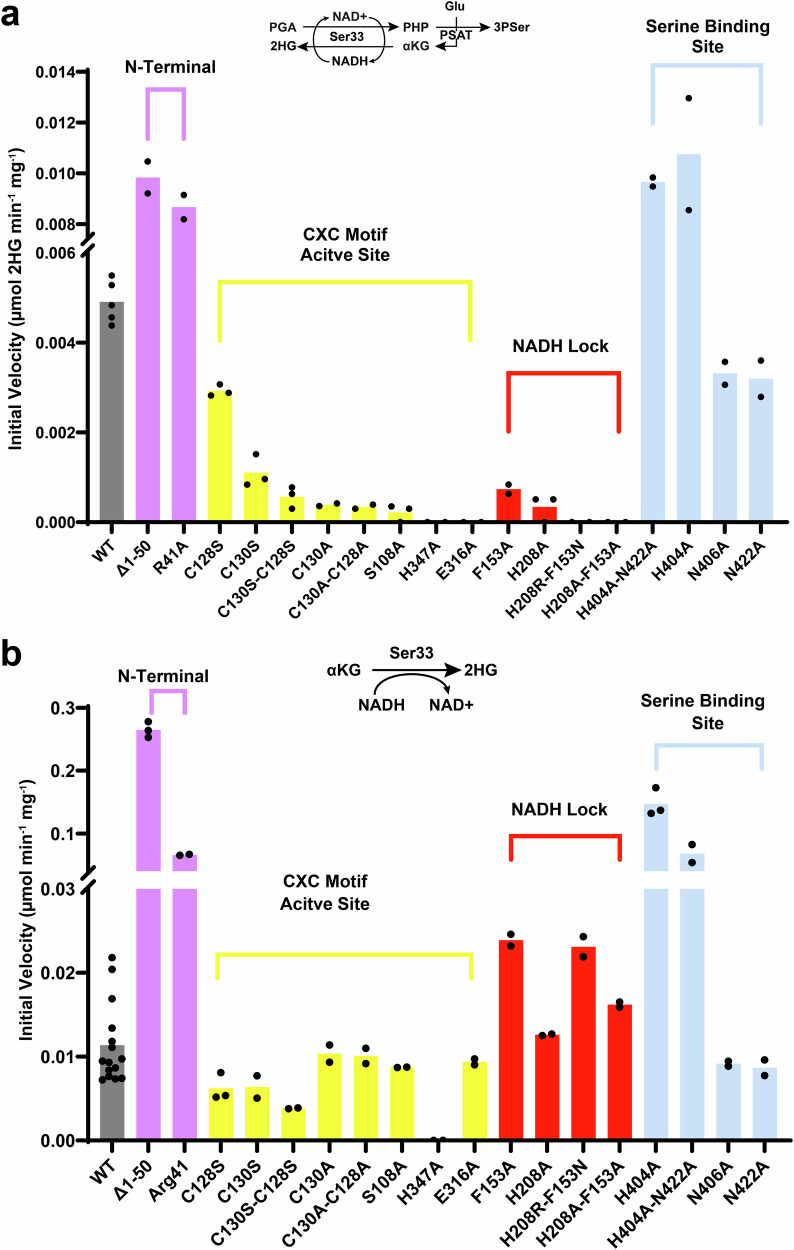
Transhydrogenase and αKG reductase activity of wild-type Ser33 and Ser33 variants. Based on the Ser33 structure, the indicated Ser33 variants and truncated proteins were selected, produced, purified, and functionally characterized. **a** The initial velocities of the transhydrogenase activity of wild-type Ser33 and Ser33 variants (40 μg/ml) were determined by measuring 2HG formation using LC-MS/MS. **b** The initial velocities of the αKG reductase activity of wild-type Ser33 (60 μg/ml) and Ser33 variants (120 μg/ml) were determined by monitoring the consumption of NADH spectrophotometrically at 340 nm. Data are shown as means of independent measurements and are represented by dots (*n* = 2–3 for Ser33 variants; *n* = 5–15 for wild-type Ser33; Source data are provided as a Source Data file).

Dependency of the Ser33 activity on αKG, but not on external NAD^+^. We investigated the role of αKG in the transhydrogenase reaction (Fig. 3; Supplementary Figs. 10 and 11; Source data are provided as a Source Data file). The reaction catalyzed by PHGDHs is thermodynamically unfavored and in vitro assays are coupled with PSAT, the enzyme catalyzing the second step of the serine biosynthetic pathway^9–12^. It is worth noting that, as a consequence of transamination, the reaction catalyzed by PSAT produces αKG, one of the substrates of Ser33^11,47,48^. We measured the enzymatic activity of Ser33 at 50 µM PGA, in a reaction mixture (i) containing αKG and NAD^+^ (complete mixture), (ii) devoid of NAD^+^, or (iii) devoid of αKG, where the initial velocity values were 0.0049 ± 0.005, 0.0043 ± 0.006 and 0.0016 ± 0.004 μmol/min.mg protein respectively (see Methods for details; Fig. 3; Supplementary Figs. 10 and 11). The reaction was not dependent on externally supplemented NAD^+^, confirming that the cofactor bound to the enzyme serves as an intermediary electron acceptor. The activity measurements also support the dependence of the Ser33 activity on αKG. The residual activity measured in the absence of αKG can be explained by sufficient PHP being generated by the first half-reaction of Ser33 (oxidation of PGA to PHP with reduction of NAD^+^) to lead to the formation of catalytic amounts of αKG via the PSAT reaction that can then be used to complete the catalytic cycle of Ser33 (oxidation of the Ser33-bound NADH by αKG with concomitant formation of 2HG). We were also able to measure an αKG reductase activity of Ser33, i.e. the ability of the enzyme to convert αKG to 2HG in the presence of externally supplemented NADH (and in the absence of PGA), displaying an initial velocity of 0.011 ± 0.004 μmol/min.mg protein (Fig. 3; Supplementary Figs. 10 and 11)^29^.

**Fig. 4:**
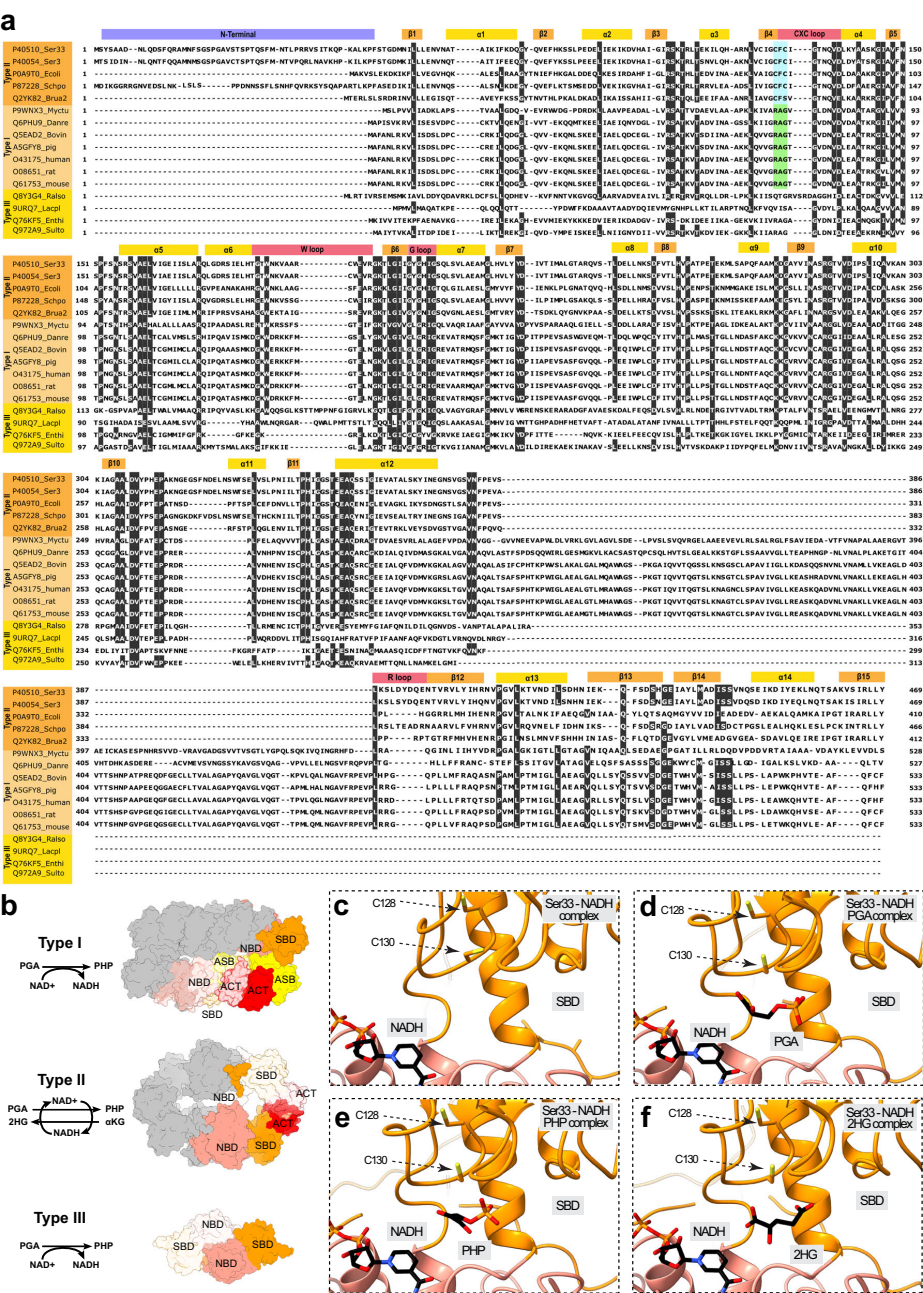
Structure-based sequence alignment of Ser33 and homologs. **a** Comparison of the Type II PHGDH from *S. cerevisiae*, Ser33 (P40510 UniProt code hereafter), with other Type II PHGDHs, *S. cerevisiae* Ser3 (P40054), *E. coli* (P0A9T0), *Schizosaccharomyces pombe* (P87228), and *Brucella abortus* (Q2YK82), Type I PHGDHs from *M. tuberculosis* (P9WNX3), *Danio rerio* (Q6PHU9), *Bos taurus* (Q5EAD2), *Sus scrofa* (A5GFY8), *H. sapiens* (O43175), *Rattus norvegicus* (O08651), and *Mus musculus* (Q61753) and Type III PHGDHs from *Ralstonia solanacearum* (Q8Y3G4), *Lactobacillus plantarum* (F9URQ7), *E. histolytica* (Q76KF5) and *Sulfolobus tokodaii* (Q972A9; see Methods). The distribution of secondary structure elements of Ser33 is displayed above the alignment. The CXC and RAG motifs identified in Type II and Type I PHGDHs are shown in blue and green, respectively. **b** A scheme of the structural arrangement of Type I, II, and III PHGDHs is shown. Close-up view of the CXC motif in the Ser33-NADH (**c**), Ser33-NADH-PGA (**d**), Ser33-NADH-PHP (**e**), and Ser33-NADH-2HG (**f**) complexes.

The Ser33 active site. To study the functional role of selected residues located in the Ser33 active site, we performed single-point mutagenesis and enzyme activity measurements in the above-described conditions to evaluate the effects on both the (i) transhydrogenase and (ii) αKG reductase activities (Fig. 3; Supplementary Figs. 10-12). According to the currently accepted mechanism, the catalytic histidine residue, H347 in Ser3 and Ser33, deprotonates the C2-hydroxyl group of PGA, facilitated by a glutamic acid residue, E316 in Ser3 and Ser33 (Figs. 1 and 3; Supplementary Fig. 9)^18,49,50^. Both the H347 and E316 residues are strictly conserved in Type I, II and III PHGDHs (Fig. 4). Although the binding of PGA, PHP, and 2HG induced only small changes in the overall Ser33 structure, it did promote important local modifications. Specifically, H347 swings its imidazole ring, positioning its Nδ1 group in close contact with the carboxylate group of E316 at electrostatic/hydrogen bonding distance (Fig. 2e-j). The replacement of H347 by alanine resulted in a catalytically inactive variant, devoid of transhydrogenase as well as αKG reductase activities (Fig. 3). The substitution of E316 by alanine also virtually abolished the transhydrogenase activity, but, intriguingly, the Ser33-E316A variant preserved the αKG reductase activity (Fig. 3). Two other residues that are also conserved in all types of PHGDHs reported so far (Fig. 4), R107 and S108, moved their side chains to approach and coordinate the phosphate groups of PGA and PHP. The replacement of S108 by alanine completely abolished the transhydrogenase activity, but did only moderately affect the αKG reductase activity (0.009 ± 0.0003 µmol/min.mg; Fig. 3).

The CXC motif. We recently identified the sequence motif CXC located in the catalytic cleft of *S. cerevisiae* Ser3 and Ser33 (C128 and C130 in Ser33) and in two bacterial PHGDHs displaying transhydrogenase activity^24^. This motif, conserved in all Type II PHGDHs, replaces a conserved RAG motif in the amino acid sequences of Type I and Type III PHGDH enzymes (Fig. 4a). Structural comparison of the unliganded (Ser33-NADH) and complexed forms of Ser33 (Ser33-NADH-PGA, Ser33-NADH-PHP, and Ser33-NADH-2HG-SER) revealed that the conformation of C128 is essentially preserved in all the crystal structures. Interestingly, the presence of PGA, PHP, or 2HG triggers a conformational rearrangement of the C130 side chain (Fig. 4c-f). To study the relevance of the two cysteine residues in the reaction mechanism, we designed single- and double-point variants in which the cysteine residues were replaced either by serine or alanine (Ser33-C128S, Ser33-C130S, Ser33-C128S-C130S, Ser33-C128A, Ser33-C130A and Ser33-C128A-C130A).

We observed that replacement of cysteine by serine residues markedly reduced the transhydrogenase activity, by 42%, 78%, and 89% for the Ser33-C128S, Ser33-C130S, and Ser33-C128S-C130S variants, respectively, compared to wild-type Ser33 (Fig. 3a). The effect was more pronounced when these cysteine residues were replaced by alanine (Ser33-C130A and Ser33-C128A-C130A), reducing the transhydrogenase activity by more than 90%. Despite extensive efforts, we were unable to produce the Ser33-C128A variant. The replacement of cysteine by serine residues also reduced the αKG reductase activity in Ser33-C128S, Ser33-C130S and Ser33-C128S-C130S by 42%, 42%, and 65%, respectively, compared to wild-type Ser33 (Fig. 3b). Intriguingly, the Ser33-C130A (0.010 ± 0.001 µmol/min.mg) and Ser33-C128A-C130A (0.010 ± 0.0009 µmol/min.mg) variants displayed near wild-type activities.

The ‘NADH-locked’ conformation. The NADH binding site, located in the NBD, is largely conserved in all three types of PHGDHs (Fig. 4a,b; Supplementary Fig. 13). Interestingly, the side chains of F153 and H208 establish a strong π-π interaction, which is conserved in Type II PHGDHs (Fig. 2; Supplementary Figs. 4-8 and 13). This interaction is absent in Type I PHGDHs, where F153 and H208 are replaced by conserved asparagine (N99 in *Hs*PHGDH) and arginine (R154 in *Hs*PHGDH) residues, respectively (Supplementary Fig. 13). This structural arrangement generates a stable, locked conformation of the NADH binding site in Ser33 and Type II PHGDH enzymes in general. This arrangement seems critical for the transhydrogenase activity, since the latter was drastically reduced ( > 90% for H208A and >85% for F153A compared to wild-type Ser33) when F153 and H208 were replaced by alanine residues (Fig. 3). This effect was exacerbated—complete loss of transhydrogenase activity—for the double variants, Ser33-F153A-H208A and Ser33-F153N-H208R (Fig. 3), the latter mimicking the homologous NBD residues in Type I PHGDHs (Supplementary Fig. 13). In stark contrast, these same Ser33 variants displayed similar or even increased αKG reductase activities compared to wild-type Ser33 (Fig. 3).

### The allosteric regulation mechanism of Ser33

**Fig. 5:**
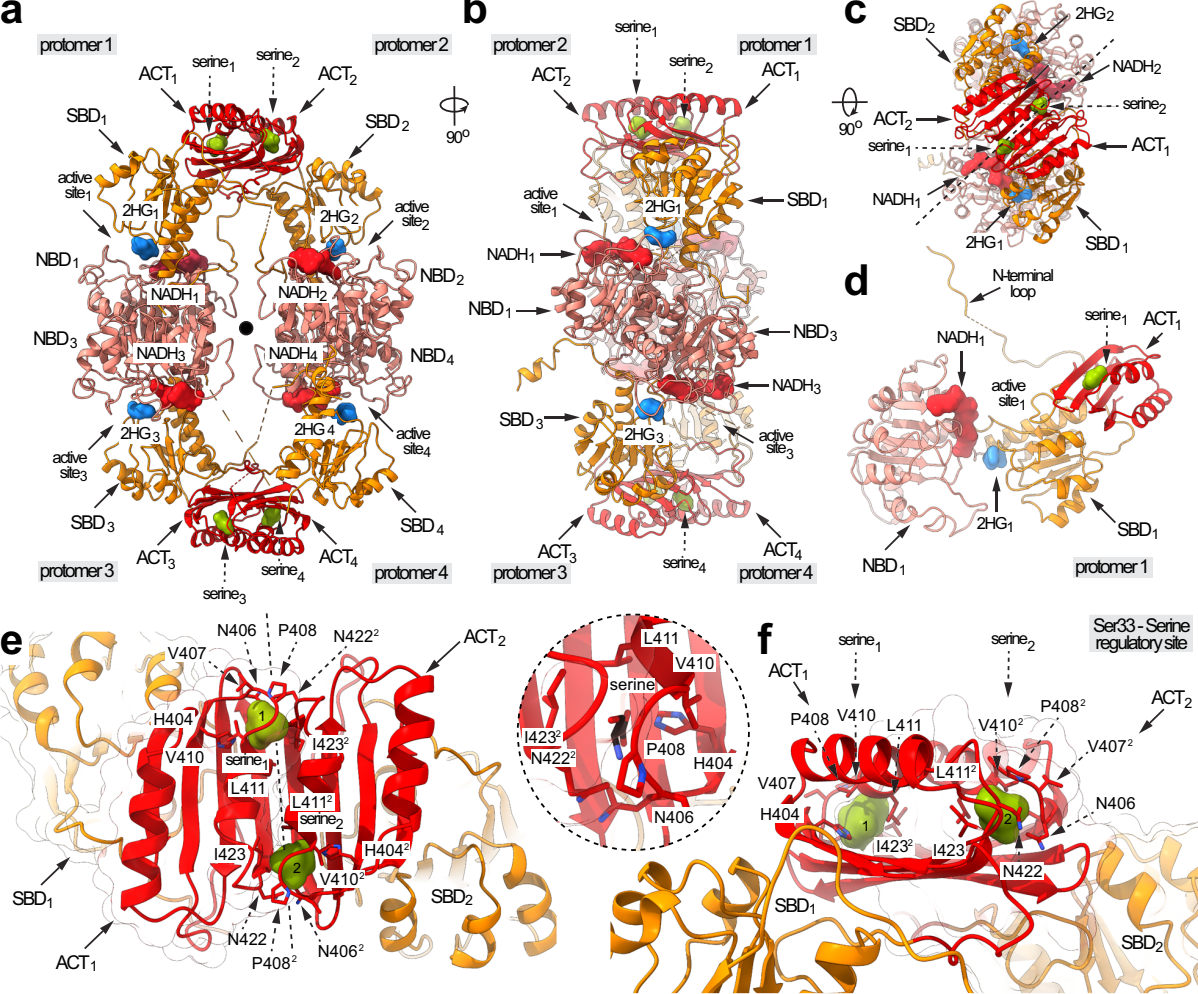
The regulatory site of Ser33. **a**–**c** The overall structure of the Ser33 homotetramer in complex with NADH, 2HG, and the allosteric regulator serine. All protomers are shown in cartoon representation, including the NBD domains (salmon), the SBD domains (orange), and the ACT regulatory domains (red). The NADH, 2HG, and serine ligands are shown in surface representation (red, blue and green, respectively). The solid circle corresponds to a 2-fold axis. **d** Close-up view of the Ser33 protomer active site, showing the location of NADH, 2HG, and serine. **e**, **f** Close-up view of the Ser33 regulatory sites in the Ser33-NADH-2HG-SER complex (PDB code 8Q2I), showing the location of serine (green).

The L-serine binding site. In several microbial species, the Ichihara-Greenberg pathway is regulated by the end product, L-serine. Ser3 and Ser33, as other Type II PHGDHs, are allosterically inhibited by L-serine^13,49,51^. To study the regulatory mechanism of Ser33, we solved the crystal structures of the enzyme in the presence of (i) L-serine, the negative allosteric regulator (Ser33-NADH-SER) and (ii) 2HG and L-serine (Ser33-NADH-2HG-SER; Fig. 5). Serine is clearly visible in the electron density maps and is present in all four allosteric sites located at the ACT-ACT” interface. Structural comparison of the Ser33-NADH-SER and Ser33-NADH-2HG-SER complexes revealed that the location of L-serine in the regulatory sites is preserved. The serine binding site is surrounded by β12 from one protomer, β13” from the neighbor protomer and the connecting loop β12-α13 (Fig. 5e,f). Specifically, the carboxylate group of serine makes hydrogen bonds with the Nε2 group of the H404 imidazole cycle, the side chain of N406 and the main chain of I423” of the neighbor protomer, and electrostatic interaction with the side chain of S428. Furthermore, the amino group of serine makes a hydrogen bond with the main chain of V407 and electrostatic interactions with the side chains of N406 and N422” from the neighbor protomer (Fig. 5e,f). The hydroxyl group of serine makes electrostatic interaction with the main chain of G409. Interestingly, inspection of the electron density maps of the Ser33-NADH, Ser33-NADH-PGA, and Ser33-NADH-PHP complexes revealed the presence of phosphate, ethylene glycol, and glycerol in the L-serine binding site (Supplementary Figs. 2 and 3).

The Ser33-H404A and Ser33-H404A-N422A variants showed a ca. 2-fold increase of the transhydrogenase activity compared to wild-type Ser33 (Fig. 3). In contrast, Ser33-N406A and Ser33-N422A reduced the transhydrogenase activity by 30-35%. Remarkably, the Ser33-H404A and Ser33-H404A-N422A variants showed 13- and 6-fold increases in the αKG reductase activity, respectively, compared to wild-type Ser33. In contrast, the Ser33-N406A and Ser33-N422A variants showed similar or slightly decreased αKG reductase activity compared to the wild-type enzyme. Kinetic analysis of the highly active H404A variant, revealed a 4-fold increase in *V*_max_ and a 4.7-fold decrease in *K*_m_ for the α-ketoglutarate reductase activity of this variant (Supplementary Fig. 14). To further understand the activation effect observed with the Ser33-H404A and Ser33-H404A-N422A variants, we measured their transhydrogenase activity in the presence of L-serine. Regardless of the amount of L-serine added (up to 500 mM), both Ser33 variants remained active, showing that they have lost the property of being allosterically inhibited by serine (Supplementary Fig. 15).

**Fig. 6:**
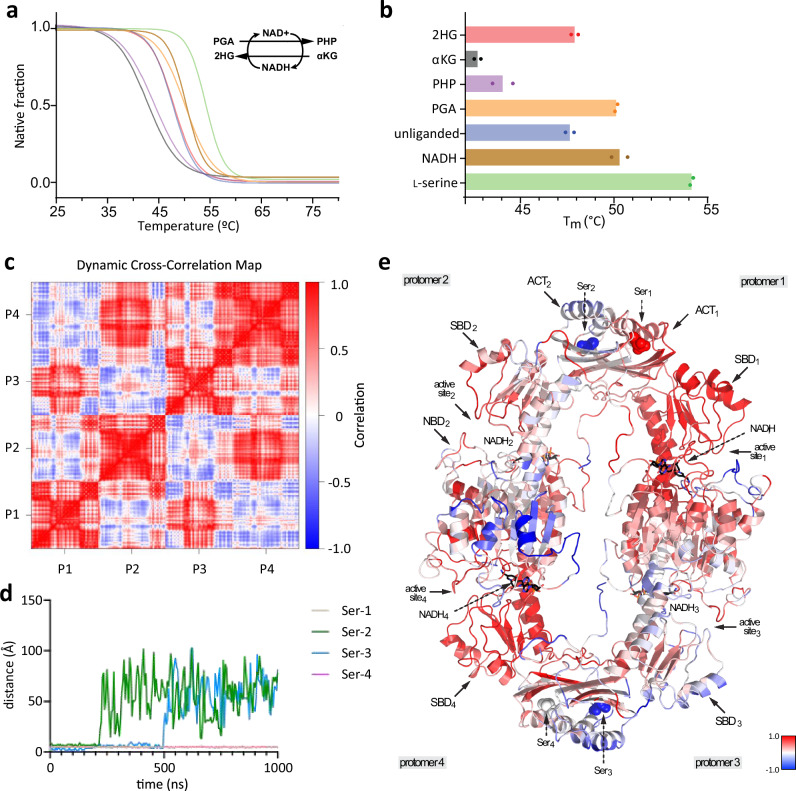
Serine-dependent modulation of inter-protomer communication. **a** Thermal unfolding transitions of Ser33 in the absence (blue) or presence of NADH (brown), PGA (orange), PHP (violet), 2HG (red), αKG (grey), or the negative allosteric regulator L-serine (green), monitored by changes in the CD signal at 222 nm. Raw data were corrected by buffer and blank subtraction, and subsequently normalized. The unfolding curves were fitted using a Boltzmann sigmoidal model in GraphPad Prism based on independent duplicates. **b** Histogram showing the mean apparent melting temperatures (*T*_M_) for each condition tested in (**a**). Dots represent *T*_M_ values from two replicates (*n* = 2). For each replicate, *T*_M_ was determined as the midpoint of the fitted transition assuming a two-state equilibrium model. **c** Dynamic cross-correlation map (DCCM) derived from 200 ns molecular dynamics simulations of the serine-bound tetramer. The color scale (–1 to 1) reflects correlation coefficients between Cα atomic motions (red, positively correlated; blue, negatively correlated), revealing protomer-specific communication patterns. **d** Time evolution of the distance between the serine carboxylate oxygen and the His408 Nε2–H proton for each protomer. Ser-2 dissociates after ~200 ns, followed by Ser-3 at ~500 ns. Notably, these two serine residues occupy diagonally opposed positions within the tetramer, indicating an asymmetric and long-range regulatory effect. **e** Structural mapping of RMSF differences between the serine-bound and serine-free states of the tetrameric complex, obtained from 1 µs molecular dynamics simulations. The protein is represented as ribbons, displaying the four protomers (1–4) with their corresponding substrate-binding domains (SBD₁–SBD₄). The color scale represents normalized RMSF changes (red, increased flexibility; blue, decreased flexibility). Serine residues are shown as spheres, and the NAD ligands are depicted as sticks with carbon atoms colored in black. Serine binding globally reduces conformational fluctuations, particularly at inter-protomer interfaces, while inducing localized flexibility in peripheral regions, consistent with an allosteric rigidification and redistribution of dynamic motions.

Interestingly, thermal unfolding followed by circular dichroism (CD) at a wavelength of 222 nm showed differences in Ser33 protein stability depending on the absence or presence of substrates or products, as well as L-serine. The apparent melting temperature (*T*_M_) of ‘unliganded’ Ser33 was 47.7 °C (Fig. 6a,b; this form is bound to co-purified NADH; Source data are provided as a Source Data file). The addition of the PGA substrate increased the *T*_M_ value to 50.1 °C, whereas the presence of the PHP product decreased the *T*_M_ to 44.0 °C. Conversely, the presence of αKG decreased the *T*_M_ value to 42.7 °C, while the addition of 2HG incremented the *T*_M_ value to 47.9 °C. The addition of L-serine incremented the *T*_M_ value to 54.1 °C, which is 6.4 °C higher than for the ‘unliganded’ form and represents the most stable conformation among the complexes tested. Addition of external NADH also led to a more moderate increase in the *T*_M_ value to 50.3 °C, likely indicating partial cofactor leakage from the Ser33 preparation during purification and/or storage.

To examine how L-serine influences inter-protomer communication in the homotetramer, we performed 1-µs molecular dynamics simulations and computed dynamic cross-correlation maps (DCCMs; Fig. 6c,d). These maps describe how the motions of different residues are correlated throughout the simulation, where red regions indicate atoms moving in concert—positively correlated—and blue regions indicate atoms moving in opposite directions (anti-correlated). When all four serine residues (Ser_1_–Ser_4_) remain bound—≈0–200 ns—the DCCM displays broad regions of positive correlation linking protomers P1–P4, consistent with highly concerted dynamics across the assembly. After ≈200 ns, Ser_2_ dissociates, followed by Ser_3_ at ≈500 ns. These two serine residues occupy diagonally opposed protomers, and their sequential loss disrupts the initially coordinated motions, suggesting an asymmetric and long-range mode of dynamic regulation within the complex. Complementary, RMSF difference analyses further support this idea (Supplementary Fig. 6e). In the serine-bound state, atomic fluctuations are globally reduced, especially at inter-protomer interfaces and within the SBD domains—SBD_1_–SBD_4_—, indicating an overall rigidification of the structure. A few peripheral loops show slightly higher flexibility, which likely reflects adaptive adjustments required to accommodate the conformational coupling triggered by serine binding. Altogether, these results indicate that serine binding reinforces long-range dynamic coupling within the tetramer, promoting structural rigidity and coherent inter-protomer communication essential for maintaining its functional integrity.

**Fig. 7:**
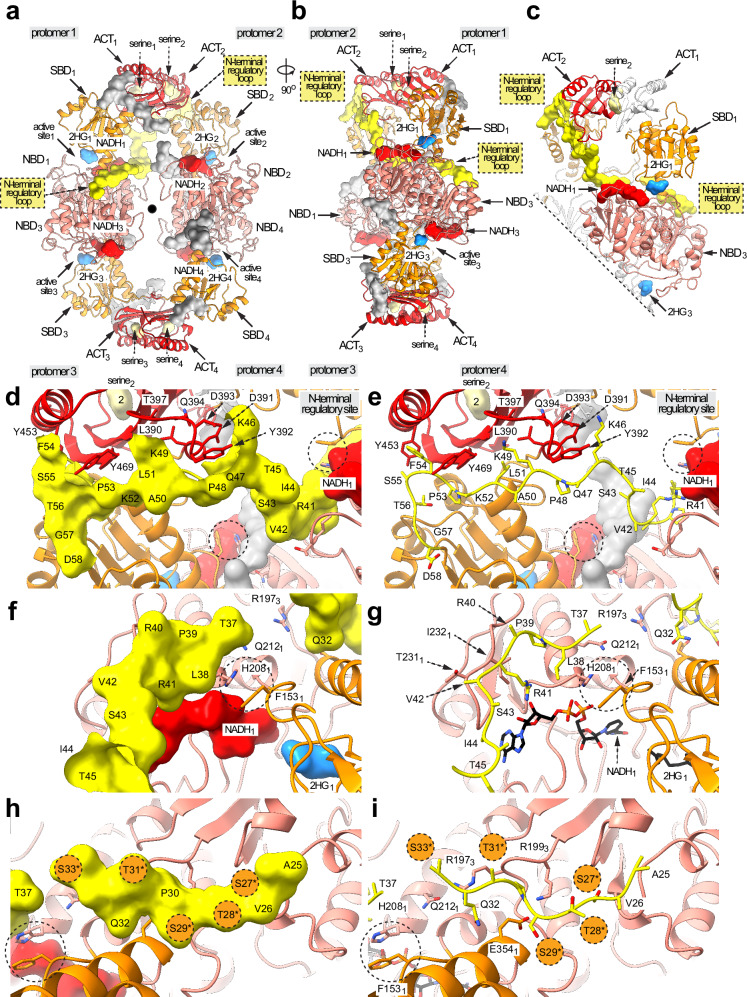
The N-terminus of Ser33. **a**–**c** The overall structure of the Ser33 homotetramer in complex with NADH, 2HG and the allosteric regulator serine. All protomers are shown in cartoon representation, including the NBD domains (salmon), the SBD domains (orange), and the ACT regulatory domains (red). The NADH, 2HG, and serine ligands are shown in surface representation (red, blue, and light yellow, respectively). The regulatory N-terminal extension of protomer 2 is shown in surface yellow representation. The solid circle corresponds to a 2-fold axis. **d**–**i** Close-up views of selected regions of the Ser33-NADH-2HG-SER structure, showing the interaction of the regulatory N-terminal extension from protomer 2 with other Ser33 protomers. The experimentally demonstrated phosphorylation sites are highlighted in orange (**h**, **i**).

The N-terminus of Ser33. Ser3 and Ser33 contain 59 extra residues at the N-terminus, compared to other Type II PHGDHs (Fig. 4a). Strikingly, residues 11 to 21 form an α-helix, whereas residues 23 to 54 comprise a long loop interacting with three domains of different protomers, including (i) the ACT domain (residues 46 to 54) of the same protomer, (ii) the NBD” domain (residues 30 to 42) of a second protomer, and (iii) the NBD”” domain (residues 23 to 29) of a third protomer (Fig. 7). Specifically, the side chain of F54 makes stacking interactions with the side chains of Y453, located in α14, and the C-terminal Y469. The side chain of K46 makes hydrogen bonds with the side chains of D391 and D393, and electrostatic interaction with the side chain of Y392. The main chain carbonyl group of Q47 makes a hydrogen bond with the main chain amino group of Y392, while the main chain amino group of K49 makes a hydrogen bond with the main chain carbonyl group of L390. The side chain of K49 makes a hydrogen bond with the main chain of Q394, and electrostatic interactions with the main chain carboxylates of T397 and Y469. The main chain amino and carboxyl groups of K52 make hydrogen bonds with the main chain carbonyl and amino groups of Y469, respectively. The side chain hydroxyl group of Y469 makes electrostatic interaction with the ε-ammonium group of K52. Two proline residues, P48 and P53, facilitate the loop adopting this structural arrangement (Fig. 7). The residues R40 and R41 facilitate the formation of an antiparallel β-bridge with I232” and T231”, with the guanidinium group of R41 making a hydrogen bond with the hydroxyl group O3B of the ribose’ ring of the AMP moiety in NADH (Fig. 7). The main chain carboxyl group of T37 makes a hydrogen bond with the side chain of H208’, whereas the side chain of N36 makes electrostatic interactions with the main chain of F153” and the side chain of Q212”. In addition, the side chain of T31 makes a hydrogen bond with the main chain carboxyl group of E354”. Finally, the side chain of S29 makes electrostatic interactions with the main chain of R197”” and the side chain of K199”” of the NBD”” domain of the third protomer.

We generated the Ser33-R41A variant and a truncated version of the enzyme lacking the first 50 residues, (Ser33-Δ1-50). Interestingly, the transhydrogenase activity increased ca. 2-fold for both variants (Fig. 3). In addition, Ser33-R41A and Ser33-Δ1-50 displayed an αKG reductase activity that was more than 6- and 20-fold increased compared to wild-type Ser33, representing the quantitatively most important effect observed among our variants (Fig. 3). A more detailed kinetic analysis of the α-ketoglutarate reductase activity of the hyperactive Δ1–50 variant revealed a 9-fold increase in *V*_max_ and a 2.4-fold decrease in *K*_m_ for α-ketoglutarate compared to wild-type Ser33 (Supplementary Fig. 14). To further elucidate the activation effect observed with the Ser33-R41A and Ser33-Δ1-50 variants, we carried out transhydrogenase activity measurements in the presence of L-serine. As depicted in Supplementary Fig. 15, the transhydrogenase activity of Ser33-R41A showed the same sensitivity for L-serine inhibition as wild-type Ser33 (IC50 of 49.7 ± 0.7 µM) whereas the Ser33-Δ1-50 variant became less sensitive to this inhibitory effect. Interestingly, for the serine binding site (Ser33-H404A and Ser33-H404A-N422A) and N-terminal domain variants that acquired hyperactivity (Fig. 3), we observed a complete loss of dependency of the transhydrogenase activity on the addition of αKG (Supplementary Fig. 11). This was not the case for any of the variants with mutations in the SBD (CXC motif, active site) or NBD (NADH lock) domains. Taken together, these observations support serine-independent regulatory effects of the N-terminus on Ser33 activity.

### Evolutionary origin and conservation of the N-terminal extension in Type II PHGDHs

**Fig. 8:**
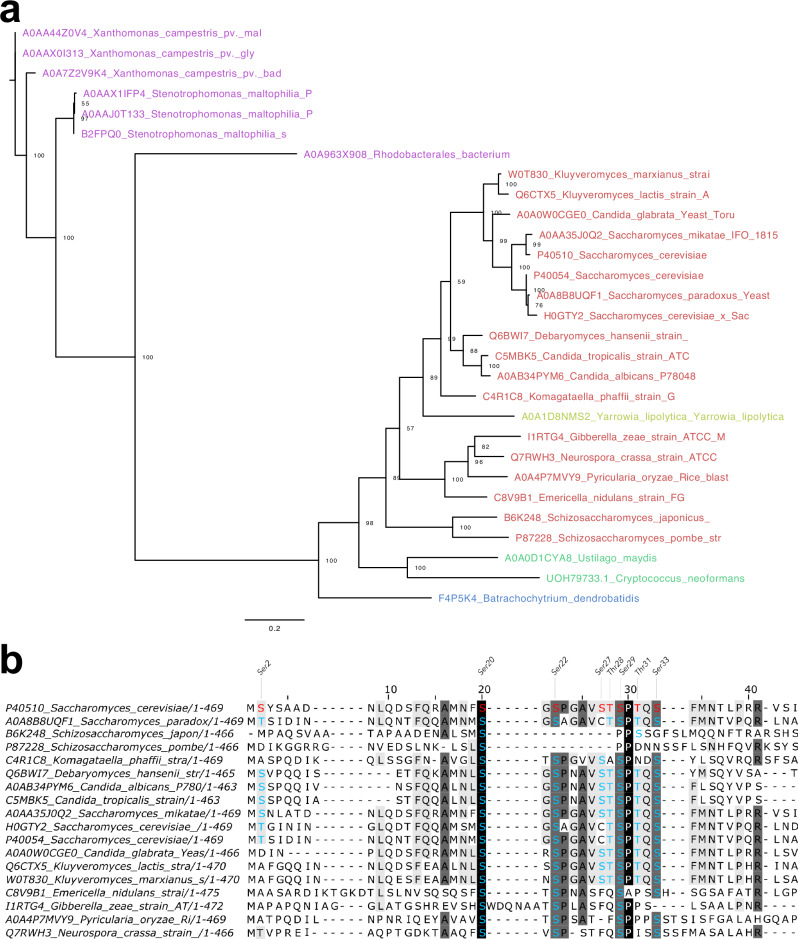
Evolutionary origins of the N-terminal extension. **a** Annotated phylogenetic tree highlighting the emergence and divergence of the N-terminal extension. Ancestral sequence reconstruction indicates that the last common ancestor of bacteria and fungi lacked the extension (purple), while a “proto-extension” emerged in the fungal ancestor (blue). This region subsequently underwent independent elongation and diversification in the Basidiomycota (dark green) and Ascomycota (orange/light green) lineages. **b** Conservation of phosphorylation sites in Ascomycota. Multiple sequence alignment of the N-terminal extension in representative species. This comparative analysis of the putative phosphorylation sites identified within the N-terminal extension of Ser33 suggests a stepwise evolutionary expansion of regulatory potential: (i) Ancestral conservation (Pan-Fungal): The residue S22 appears to represent the most ancient regulatory feature. It is conserved across all major fungal lineages analyzed, including Basidiomycota (Ustilago, Cryptococcus), Chytridiomycota (Batrachochytrium), and fission yeasts (*S. pombe*). This suggests S22 was present in the fungal common ancestor; (ii) Ascomycota expansion: Following the divergence of fission yeasts, the N-terminal extension acquired additional regulatory elements in the common ancestor of filamentous fungi (Pezizomycotina) and budding yeasts (Saccharomycotina). This includes residue S20, which is conserved in Saccharomyces and Candida as well as filamentous species like Emericella and Gibberella. Similarly, the C-terminal motif housing residues S29-S33 is a shared feature of these lineages but is absent in the earlier-branching fission yeasts; (iii) Yeast-Specific Differentiation: The residue S27 shows a specific pattern of divergence within the budding yeasts. While highly conserved as a serine in *S. cerevisiae* Ser33 (P40510) and related species (e.g., Candida, Kluyveromyces), the paralog Ser3 (P40054) has diverged to a cysteine at this position. This pattern supports a conserved regulatory role for the N-terminal extension that is anchored by an ancient phospho-site (S22), expanded with additional sites (S20, S29-S33) in the Ascomycota lineage, and further fine-tuned by gene duplication events in budding yeasts.

To better understand the functional significance of the N-terminal extension of *S. cerevisiae* Ser33, we performed an expanded phylogenetic and comparative sequence analysis of Type II PHGDHs (Fig. 8). A curated dataset of Type II PHGDH orthologs was assembled, spanning prokaryotes and representative fungal lineages, including Ascomycota, Basidiomycota, and Chytridiomycota, all defined by the presence of the characteristic Type II CXC motif. Phylogenetic reconstruction revealed a clear separation between prokaryotic and fungal Type II PHGDHs (Supplementary Fig. 16). All bacterial orthologs lack an N-terminal extension, whereas all fungal sequences possess an additional N-terminal region, indicating that the extension is a eukaryotic innovation absent from prokaryotes (Fig. 8). Ancestral sequence reconstruction further indicated that the last common ancestor of bacteria and fungi lacked the extension, while the inferred ancestor of all fungi already contained a short homologous “proto-extension”. Within fungi, the N-terminal extension underwent lineage-specific elaboration. Early-branching Chytridiomycota species retain only a short version of the extension, whereas both Ascomycota and Basidiomycota independently evolved longer N-terminal regions following their divergence. These extensions are now sequence-divergent and non-alignable between fungal phyla, indicating independent evolutionary trajectories originating from a shared ancestral feature.

Focusing on Ascomycota, a multiple sequence alignment of 19 representative Type II PHGDHs revealed that all possess a substantial N-terminal extension ranging from 45 to 92 amino acids. Excluding the outlier *Yarrowia lipolytica*, which harbors an unusually long extension ( > 90 aa), the remaining Ascomycota sequences cluster tightly around a mean extension length of ~51 amino acids, with low variance across taxonomic groups (Fig. 8). Statistical analysis showed a strong phylogenetic signal for extension length (Pagel’s λ = 1.0), indicating that extension length is evolutionarily constrained and shaped by shared ancestry rather than independent gain or loss events. Notably, both *S. cerevisiae* paralogs, Ser3 and Ser33, exhibit identical extension lengths, indicating that the extension predates the gene duplication event. Mapping functionally characterized residues from Ser33 onto the expanded alignment showed that catalytic residues—S108, E316, H347—, the CXC motif—C128 and C130—as well as cofactor-binding residues—F153, H208—and allosteric sites—H404, N406, N422—are highly conserved across fungal lineages.

## Discussion

In this study, we provide structural, biochemical and biophysical evidence supporting that Ser33 from *S. cerevisiae* catalyzes a transhydrogenase reaction, coupling the oxidation of PGA to PHP with the reduction of αKG to 2HG. We visualize how Ser33 recognizes the NADH cofactor, tightly bound to all NBD domains of the tetrameric enzyme (Fig. 1). We found that the conserved F153 and H208 residues are critical in generating a stable, locked conformation of the NADH/NAD^+^ binding site of the enzyme (Supplementary Fig. 13b). Mutation of these residues, individually, strongly reduced the transhydrogenase activity of Ser33 and double variants were completely inactive. Remarkably, mutation of these same residues did not affect or even increased the αKG reductase activity. This aligns with the fact that for the reductase activity, the NAD^+^ cofactor is exchanged with the medium—net NADH consumption—and does not remain locked in the active site for oxidation-reduction cycles. However, attempts to detect a PGA dehydrogenase activity by measuring net NADH production with these NADH lock variants remained unsuccessful. This suggests that, even if the NADH lock residues are mutated, NAD^+^ cannot displace enzyme-bound NADH for sustaining PGA oxidation—while NADH can displace enzyme-bound NAD^+^ to sustain αKG reduction—, and that additional structural features, in addition to the NADH lock arrangement, must contribute to the dehydrogenase-transhydrogenase transition. As suggested before^24,52^, and further supported by the present study, a CXC motif in transhydrogenases, that is replaced by a RAG motif in type I PHGDHs, also plays an important role in this mechanistic switch of the enzyme; this point is further developed below.

It is worth noting that NADH tightly binds not only to Ser33 but also to *Ec*PHGDH and *Ps*PHGDH, for which the *K*_D_ values were 0.01 and 0.1 μM, respectively^12,53^. *Ec*PHGDH and *Ps*PHGDH follow an ordered Bi Bi mechanism, with the NADH cofactor binding first, and the substrate second^54–56^. Altogether, for the Ser33 transhydrogenase activity, the experimental data support the occurrence of an internal cycle of NAD^+^/NADH interconversion coupled with the reduction of αKG to 2HG; cofactor recycling is dependent on αKG as shown by our activity measurements. Interestingly, intracellularly the cycle is maintained for Ser33 and other members of the Type II family of PHGDHs, by the production of αKG by the second enzyme in the pathway, the PSAT transaminase. The third and last step of the pathway consists in the irreversible dephosphorylation of phosphoserine by PSP, which further pulls the L-serine pathway in the direction of L-serine production. Type II PHGDHs are allosterically inhibited by L-serine, leading to negative feedback regulation of the L-serine synthesis pathway by its end product in prokaryotic and yeast species.

Type II family PHGDHs are grouped as transhydrogenase enzymes displaying a highly conserved CXC motif, located in the active site at the SBD domains of tetramers (Fig. 4). In Type I and Type III PHGDHs, the CXC motif is replaced by a RAG motif^24^. As observed in the five crystal structures reported in this work, the CXC motif, particularly C130, plays a determinant role in the coordination of all substrates/products and mutation of the corresponding cysteines, individually or simultaneously, affects the transhydrogenase activity. Specifically, replacement of these cysteine residues with alanine, produced a more marked decrease in activity compared to the variants where the cysteines were replaced by serine residues (Fig. 3). A possible explanation of the decreased activity of the Cys to Ser variants is that serine residues might establish additional hydrogen bonds with αKG, slowing down the release or exchange of the substrate. Conversely, the replacement of R72—equivalent to position C128 in Ser33—by alanine in *Mtb*PHGDH, a classical Type I dehydrogenase enzyme—containing a RAG motif instead of the CXC motif—, allowed the variant to utilize αKG as a substrate, further supporting that this motif plays an important role in the dehydrogenase versus transhydrogenase activity in PHGDHs by enabling the active site to accommodate αKG or not^52^.

**Fig. 9:**
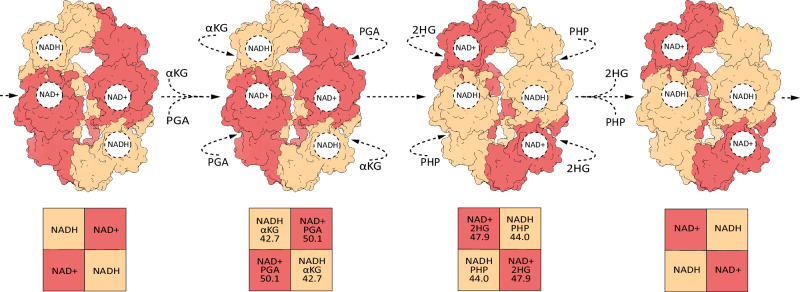
A model of catalytic ‘flip-flop’ mechanism for the Ser33 multidomain homotetramer. Surface representation of the Ser33 homotetramer, showing the simultaneous conversion of the substrates NADH/αKG and NAD + /PGA. The *T*_M_ values of the substrates and corresponding products NAD + /2HG and NADH/PHP are shown.

Ser33, as other Type II PHGDHs, including *Ec*PHGDH^30^, can be viewed as a dimer of dimers. The primary dimer is formed by the interaction of two protomers at each of their NBD domains. The tetramer is completed by the interaction of the four ACT regulatory domains comprising two interfaces at each end of the tetramer. The use of engineered asymmetric hybrid tetramers of *Ec*PHGDH demonstrated that catalytic dimers can act independently of each other, able to carry out the transhydrogenase reaction (Fig. 1)^28,55^. This suggests a ‘flip-flop’ mechanism in which two active sites of the tetramer catalyze the conversion of PGA and NAD^+^ to PHP and NADH, while the other two active sites catalyze the conversion of αKG and NADH to 2HG and NAD^+^ and vice-versa as the reactions progress (Fig. 9). Specifically, active sites found diagonally across the NBD domain interface are active at the same time and that activity flip-flops from one diagonal pair to the other during turnover^9^. Interestingly, the *T*_M_ of unliganded Ser33 was 47.7 °C (Fig. 6a,b). The addition of PGA increased the *T*_M_ value to 50.1 °C, whereas the presence of αKG decreased the *T*_M_ to 42.7 °C. Conversely, the presence of the PHP product decreased the *T*_M_ value to 44.0 °C, while the addition of 2HG incremented the *T*_M_ value to 47.9 °C. Therefore, considering the simultaneous conversion of the substrate pairs, two active sites of the tetramer occupied by PGA/NAD^+^ are more stable than the two active sites occupied by αKG/NADH (Fig. 9). After the transhydrogenase reaction has occurred, the two active sites harboring the PHP/NADH products are less stable compared to those comprising the 2HG/NAD^+^ products. This might facilitate the substrate recognition and product release transitions/processes (Fig. 9).

Allosteric enzymes play critical roles in cell metabolism regulation and homeostasis. A perturbation at the allosteric site is transmitted and functionally connected to the active site of the enzyme through a network of amino acids^56,57^. Allosteric site perturbations are mediated by diverse regulatory mechanisms mainly comprising the binding of small signaling molecules at the regulatory site(s), protein-protein interactions, protein oligomerization, and post translational modifications, including phosphorylation events^58,59^. As a consequence of subtle or important changes in conformation and dynamics, the population of active and inactive states is altered, thus regulating the kinetics and activity of the enzymes^56,57^. Ser33, as other Type II PHGDHs, is allosterically inhibited by L-serine^24,31^. The transhydrogenase activity of Ser33 seems to be much more sensitive to L-serine inhibition than the one of Ser3, with IC_50_ values of 61 ± 5 and 590 ± 54 μM, respectively, in the presence of 50 μM PGA^24^. The IC_50_ value of L-serine for *Ec*PHGDH is 10 μM^9,19^. *Ec*PHGDH tetramers containing only two intact L-serine binding sites show positive cooperativity and produce almost complete inhibition of the enzymatic activity, when these two allosteric L-serine sites are on opposite sides of the NBD interface. The allosteric signal is transmitted through a G336-G337 hinge connecting the regulatory ACT domain to the SBD domain^26^. It is worth noting that the replacement of G336-G337 by valine residues markedly increased the IC_50_ value of *Ec*PHGDH for L-serine to 800 μM^60^. It was proposed that L-serine binding leading to inhibition of enzyme activity requires a domain rotation of ca. 6° in two of the four protomers of *Ec*PHGDH that are oriented diagonally across from each other in the tetrameric architecture^23,26^. However, these Gly residues are not conserved in the Ser33 sequence, where they are replaced by Asn396 and Thr397, and located in a much longer loop—residues 372 to 398—than that observed in the *Ec*PHGDH structure (Fig. 4). This loop directly interacts with the large N-terminal extension observed in Ser33 and is absent in *Ec*PHGDH and other Type II PHGDHs, supporting that other structural elements are involved in the regulatory mechanism of Ser33. Interestingly, the *T*_M_ values of Ser33-NADH and Ser33-NADH-SER were 47.7 and 54.1 °C, respectively, indicating that the L-serine-complexed form is considerably more stable than the Ser33-NADH form (Fig. 6a,b). Our 1-µs molecular dynamics simulations on the Ser33-NADH-SER complex revealed that the presence of two serine residues on diagonally opposed protomers disrupts the initially coordinated motions, indicating an asymmetric, long-range mode of dynamic regulation within the complex (Fig. 6c-e). Collectively, these findings support a negative regulatory mechanism mediated by L-serine that is partially conserved between prokaryotic and eukaryotic Type II PHGDHs.

Another surprising discovery was that the long N-terminal extension—residues 1 to 59—, directly connected to the SBD domain of Ser33, interacts with (i) the ACT domain of the same protomer, (ii) the NBD” domain of a second protomer, including the anchoring of R41 to the ribose’ ring of the adenosine group of NADH, and (iii) the NBD”” domain of a third protomer, including the WL”” loop, located in the active site and in close contact with substrates/products (Fig. 7). In addition, the NBD domain is associated to the NBD”’ domain of a neighbor protomer. Therefore, all subunits and all active sites of Ser33 communicate with each other (Fig. 7). The crystal structures of the Ser33-NADH-SER and Ser33-NADH-2HG-SER complexes reveal that the N-terminal extensions adopt similar conformations, coordinating the four NADH cofactors of neighbor protomers. In contrast, the crystal structure of Ser33-NADH reveals two different conformations for the N-terminal extensions. Strikingly, the Ser33-Δ1-50 variant, in which the first 50 residues were deleted, showed markedly increased transhydrogenase and αKG reductase activities compared to the full-length enzyme, supporting an important role of the N-terminal extension in the regulatory mechanism of Ser33 (Fig. 3). The impressive, more than 20-fold increase in the αKG reductase of the Ser33-Δ1-50 variant compared to the wild-type enzyme, indicates that this N-terminal extension maintains the enzyme in a repressed state. It is remarkable also that the transhydrogenase activity becomes less or completely independent on externally supplemented αKG for the Ser33-Δ1-50 variant, as well as for other variants with strongly increased αKG reductase activities (Ser33-H404A, and Ser33-H404A-N422A; Supplementary Fig. 11). This may be explained by an increased affinity of the transhydrogenase for αKG, as at least indirectly suggested by our kinetic analyses of the αKG reductase activity of the Ser33-Δ1-50 and Ser33-H404A variants (Supplementary Fig. 14); consequently, the low amounts of αKG produced by the PSAT coupling reaction may be sufficient to sustain maximal transhydrogenase activity. Concerning the propensity of Ser33 of being inhibited by L-serine, despite the activation effects of the R41A and Δ1-50 mutations, these variants retain their sensitivity to L-serine inhibition, although it is reduced in the Ser33-R41A variant. This indicates that L-serine-independent regulatory mechanisms exist. Another observation that is in line with a regulatory role of this N-terminal extension independent of L-serine is that both the Ser3 and Ser33 proteins contain this N-terminal extension (including the R41 and K46 residues) although Ser3 is much less sensitive to L-serine inhibition than Ser33^24^.

The close inspection of the extra residues at the N-terminus of Ser33 reveals 15 threonine and serine candidates for protein phosphorylation, including S2, S4, S12, S20, S22, S27, T28, S29, T31, S33, T37, S43, T45, S55 and T56 (Fig. 7). The phosphorylation of eight of them was experimentally determined, including S2^61^, S20^62–65^, S22^61–63,65–67^, S27^63^, T28^63,66^, S29^62,63,65–67^, T31^63,65,66^ and S33^63^. The S20 and S22 residues are located at the N-terminal α-helix. Interestingly, (i) S27, T28 and S29 are in close contact with the WL loop of protomer””, containing K188””, which directly interacts with substrates/products involved in the transhydrogenase mechanism; and (ii) T31 and S33 are in close contact with the β5-α5 loop of the active site (residues 150-155) of protomer 2, containing F153”, a residue that establishes a strong pi-pi interaction with H208”, essential for maintaining the locked conformation of the NADH binding site in Ser33 and Type II PHGDH enzymes, suggesting a role for protein phosphorylation in Ser33 activity regulation.

The evolutionary analyses presented here provide important context for interpreting the functional role of the N-terminal extension of Ser33 (Fig. 8). Rather than being a yeast-specific appendage, this region represents an ancient fungal innovation that emerged after the divergence of fungi from prokaryotes and subsequently diversified across fungal lineages. The tight constraint on extension length within Ascomycota, together with its presence in both *S. cerevisiae* paralogs, argues strongly for a conserved regulatory function rather than a dispensable or idiosyncratic feature. Although the N-terminal extension exhibits lower overall sequence conservation than the catalytic core, the S22 residue is conserved across the entire fungal kingdom, implying an ancient and fundamental regulatory mechanism. Subsequent evolutionary events in the Ascomycota lineage expanded this regulatory potential through the acquisition of S20 and the S29-S33 motif, which are shared by filamentous and budding fungi but absent in fission yeasts. This pattern is consistent with a model in which the extension acts as a flexible regulatory module, integrating post-translational signals to modulate enzyme activity rather than contributing directly to catalysis. While direct experimental validation of phosphorylation-dependent regulation remains an important goal for future work, the strong evolutionary conservation of both the extension and specific phospho-acceptor residues supports the biological relevance of this regulatory mechanism.

Very recently, it was reported that Ser3 and Ser33 physically interact with Pib2, a cysteine sensor that activates TORC1, and that Ser3 is phosphorylated by TORC1 (Ser33 was not tested in that study, but is also a likely substrate)^68^. The phosphorylated Ser3 residues have not been identified yet and the effect of the phosphorylation on Ser3 activity was also not yet investigated. However, together with our structural-functional analyses, it can be speculated that phosphorylation of the N-terminus of Ser3 and Ser33 may be involved in metabolic adaptations in response to changing cysteine levels mediated by Pib2 and TORC1.

It has been proposed since the 1970’s that human PHGDH is involved in cancer^69^ and intense subsequent research efforts have revealed a critical role for increased levels of this rate-limiting serine synthesis enzyme in cancer cell proliferation^2,70^. Very recently, low PHGDH levels have been shown to potentiate metastatic dissemination in breast cancer, further expanding the mechanistic spectrum of PHGDH involvement in cancer pathology^71^. Consequently, human PHGDH has emerged as an attractive target for cancer therapy, notably via the design of human PHGDH inhibitors^72^. Germline mutations in human PHGDH, on the other hand, are known to lead to rare neurological disorders whose clinical presentation spans a spectrum, from severe neurodevelopmental infantile forms to milder, progressive adult forms^73,74^. It is worth noting that, in mammals, L-serine synthesis is organ specific, with the brain relying on glucose and the kidney and liver utilizing glycine^1^. This, combined with a generally low permeability of the blood brain barrier to L-serine, explains that deficiency in the de novo L-serine biosynthetic pathway leads to severe neurological phenotypes in humans, including microcephalia, seizures, and severe psychomotor retardation^74–78^. From a more fundamental point of view, it has only recently been discovered that microbial Type II PHGDHs employ a distinct catalytic mechanism to oxidize PGA to PHP in the conserved de novo serine synthesis pathway compared to human and other type I PHGDHs. This opens the interesting perspective of finding small molecules that differentially affect human PHGDH and homologs in pathogenic microbes. Ser33 is a eukaryotic Type II PHGDH, containing a catalytic core, comprising both NBD and SBD domains, and the regulatory ACT domain; while human Type I PHGDH—31.17% sequence identity with Ser33—presents an additional ASB domain. Our experimental data also point to different regulatory mechanisms between both enzymes (Supplementary Fig. 17). Ser33, but not human PHGDH, is inhibited by l-serine. Interestingly, human PHGDH can be phosphorylated under physiological, non-pathological, and pathological conditions, despite lacking the N-terminal extension found in Ser33^79^. Overall, the regulation of L-serine synthesis in mammals remains poorly understood; however, a recent study indicates that the three enzymes of the serine synthesis pathway (PHGDH, PSAT, and PSP) assemble into a dynamic, metabolically active complex in differentiated human astrocytes, termed the ‘serinosome’, raising the possibility that regulation may occur at the level of assembly of this metabolic cluster^80^.

Given the central role of PHGDH as a gatekeeper for regulating carbon flux from glucose to serine, a precursor with multiple crucial downstream metabolic fates, and the proven role of the human homolog in diseases, it is essential to progress in our understanding of its structure and regulatory properties, which we contribute here by using the *S. cerevisiae* Ser33 protein as a model. Our structural-functional studies of this eukaryotic type II PHGDH enzyme identified residues that critically affect the active site (CXC motif), cofactor binding (NADH lock), and inhibition by serine (ACT domain) and provide evidence that in the yeast enzyme, the N-terminal extension plays an additional role in regulating the enzyme, independently of serine, and potentially involving protein phosphorylation.

## Methods

### Cloning, expression, and purification of Ser33 and variants

The bacterial expression vector pDEST527 (IPTG-inducible T7 promoter, N-terminal His_6_-tag) encoding the wild-type and variant/truncated version of Ser33 was generated by ATG:biosynthetics^24,81^. The bacterial expression vector pET15b (IPTG-inducible T7 promoter, N-terminal His_6_-Tag) containing the human PSAT1 (phosphoserine aminotransferase) coding sequence, was kindly provided by Emile Van Schaftingen (de Duve Institute, Belgium). The recombinant wild-type Ser33 (486 residues, NP_012191.1) and variant/truncated versions have an N-terminal extension of 17 amino acids, including the histidine tag (Supplementary Fig. 1). Wild-type and variant/truncated Ser33 proteins were expressed in *Escherichia coli* BL21(DE3) cells. Transformed cells were grown at 37 °C with shaking in LB medium supplemented with 100 μg ml^−1^ ampicillin. When the culture reached an OD_600_ value of 0.6–0.8, expression was induced by adding 1 mM IPTG. After approximately 16 h at 18 °C with shaking, the cells were harvested by centrifugation at 5000 × *g* for 20 min at 4 °C and resuspended in 25 ml of a lysis buffer containing 50 mM Tris-HCl, pH 7.5, 500 mM NaCl, 20 mM imidazole, 260 U DNAse (Invitrogen, 18047019), and protease inhibitors (Thermo Scientific™, A32955). Cells were disrupted by sonication (21 cycles of 5-sec pulses followed by 60 s cooling, 50% amplitude) at 4 °C, and the lysate was centrifuged at 17,000 × *g* for 30 min at 4 °C. The supernatant was purified by Ni^2+^-affinity chromatography on a HisTrap column (1-5 ml, Cytiva) equilibrated in a buffer containing 50 mM Tris-HCl, pH 7.5, 500 mM NaCl, and 20 mM imidazole. After washing the column with the equilibration buffer, the elution was performed with a 20-min linear imidazole gradient (20 to 250 mM) in 50 mM Tris-HCl, pH 7.5, 500 mM NaCl at 1 ml min^−1^. The protein was further purified by size-exclusion chromatography (SEC) using a Superdex 200 10/30 GL column (Cytiva) equilibrated in 25 mM Tris-HCl pH 7.5, 150 mM NaCl (Supplementary Fig. 1). The concentration of the purified protein was estimated based on A_280_ and an extinction coefficient estimated using ProtParam (https://web.expasy.org/protparam/). For the crystallization experiments, wild-type Ser33 protein was dialyzed, before SEC, against a buffer containing 50 mM Tris-HCl, pH 8.0, 150 mM NaCl, 1 mM DTT, 0.5 mM EDTA, and TEV protease (1:10 ratio with respect to Ser33), overnight at 18 °C. The completeness of the enzymatic digestion reaction was confirmed by SDS-PAGE. The purity of all final Ser33 and PSAT1 protein preparations was estimated to >95% by SDS-PAGE and Coomassie staining (Supplementary Fig. 1; Source data are provided as a Source Data file). They were stored at -80 °C after adding 10 % glycerol.

### Ser33 crystallization conditions and data collection

Crystals seeds of Ser33 were obtained by mixing 0.2 µL of protein solution at 7.5 mg ml^-1^ in 25 mM Tris-HCl pH 7.5, 150 mM NaCl with 0.2 µL of a mixture of several Amino acids (containing 20mM M L-Na-glutamate, 20 mM alanine, 20 mM glycine, 20 mM lysine HCl, 20 mM serine), 0.1 M of a mixture HEPES:MOPS pH=7.5 and 50% v/v of a precipitant mixed solution containing 25% v/v 2-methyl-2,4-pentanediol (MPD), 25% PEG 1000 and 25% w/v PEG 3350 (Morpheus® protein crystallization screen). The crystals obtained in this condition were resuspended in 50 µL of the precipitant solution 10% more concentrated. The mixture was vortexed by using 3-4 beads (Ceramic) for 3 min. After adding 450 µL of the mixture, the seeds were freeze dried and kept at −80 °C.

Ser33 in its unliganded form was crystalized by mixing 0.2 µL of protein solution at 7.5 mg ml^−1^ in 25 mM Tris-HCl pH 7.5, 150 mM NaCl and 0.05 µL of the seeds described above with 0.25 µL of 0.1 M potassium sodium tartrate tetrahydrate and 10% w/v of PEG 3350 (PACT premier® protein crystallization screen). Crystals grew in 1–2 days. They were transferred to a cryo-protectant solution containing 10% of glycerol and frozen in liquid nitrogen. Complete X-ray diffraction datasets were collected in beamline BL13-XALOC at ALBA facilities (Cerdanyola del Valles, Spain). Ser33 unliganded crystallized in the *P*2_1_2_1_2_1_ space group with four molecules in the asymmetric unit and diffracted to a maximum resolution of 3.19 Å (Supplementary Table 2).

Ser33 in complex with PGA was crystalized by mixing 0.2 µL of protein solution at 7.5 mg ml^−1^ in 25 mM Tris-HCl pH 7.5, 150 mM NaCl and 0.05 µL of the seeds described above, with 0.25 µL of 0.2 M 1,6-hexanediolm 0.2 M 1-butanol, 0.2 M 1,2-propanediol, 0.2 M 2-propanol, 0.2 M 1,4-butanediol, 0.2 M 1,3-propanediol, 0.1 M sodium HEPES; MOPS (acid) pH 7.5 and 40% v/v glycerol and 20% w/v PEG 4,000 (Morpheus® protein crystallization screen). PGA was added to the crystallization mix at a final concentration of 5 mM. Crystals grew in 1–2 days and were frozen in liquid nitrogen. Complete X-ray diffraction datasets were collected in beamline BL13-XALOC at ALBA facilities (Cerdanyola del Valles, Spain). Ser33 in complex with PGA crystallized in the *P*1 space group with four molecules in the asymmetric unit and diffracted to a maximum resolution of 2.7 Å (Supplementary Table 2).

Ser33 in complex with PHP was crystalized by mixing 0.2 µL of protein solution at 7.5 mg ml^-1^ in 25 mM Tris-HCl pH 7.5, 150 mM NaCl and 0.05 µL of the seeds described above with 0.25 µL of 0.1 M SPG (succinic acid, sodium phosphate monobasic monohydrate, glycine pH 6.0), 25% w/v PEG 1,500 (PACT premier® protein crystallization screen). PHP was added to the crystallization mix at a final concentration of 5 mM. Crystals grew in 1–2 days. They were transferred to a cryo-protectant solution containing 10% of ethylene glycol and frozen in liquid nitrogen. Complete X-ray diffraction datasets were collected in beamline I24 at Diamond Light Source (DLS, Oxfordshire, UK). Ser33 in complex with PHP crystallized in the *P*1 space group with four molecules in the asymmetric unit and diffracted to a maximum resolution of 2.6 Å (Supplementary Table 2).

Ser33 in complex with 2HG and L-serine was crystalized by mixing 0.2 µL of protein solution at 7.5 mg ml^−1^ in 25 mM Tris-HCl pH 7.5, 150 mM NaCl and 0.05 µL of the seeds described above with 0.25 µL of 0.2 M sodium citrate tribasic dihydrate, 0.1 M Bis-Tris propane pH 8.5, 20 % w/v PEG 3350 (PACT premier® protein crystallization screen). 2HG was added to the crystallization mix at a final concentration of 5 mM, and a quick soaking was done with a solution containing the crystallization conditions and L-serine at a concentration of 5 mM. Crystals grew in 1–2 days. They were transferred to a cryo-protectant solution containing 15% of glycerol and frozen in liquid nitrogen. Complete X-ray diffraction datasets were collected in beamline BL13-XALOC at ALBA facilities (Cerdanyola del Valles, Spain). Ser33 in complex with 2HG and L-serine crystallized in the *P*1 space group with eight molecules in the asymmetric unit and diffracted to a maximum resolution of 2.51 Å (Supplementary Table 2).

Ser33 in complex with L-serine was crystalized by mixing 0.2 µL of protein solution at 7.5 mg ml^−1^ in 25 mM Tris-HCl pH 7.5, 150 mM NaCl with 0.05 µL of the seeds described above and 0.25 µL of 0.3 M diethylene glycol, 0.3 M triethylene-glycol, 0.3 M tetraethylene glycol, 0.3 M pentaethylene glycol, 0.1 M of a mixture sodium HEPES, MOPS pH 7.5, 40% v/v glycerol and 20% w/v PEG 4000 (Morpheus® protein crystallization screen). L-serine was added to the crystallization mix at a final concentration of 5 mM. Crystals grew in 1–2 days and were frozen in liquid nitrogen. Complete X-ray diffraction datasets were collected in beamline I24 at Diamond Light Source (DLS, Oxfordshire, UK). Ser33 in complex with L-serine crystallized in the *P*1 space group with eight molecules in the asymmetric unit and diffracted to a maximum resolution of 2.69 Å (Supplementary Table 2). All the datasets were scaled and integrated using XDS accordingly^82^.

### Structure determination, refinement, and analysis

The structure determination of Ser33 in complex with NADH (Ser33-NADH) was performed by molecular replacement methods implemented in Phaser^83^ and the PHENIX suite (version 1.19.2)^84^, using the structure of the phosphoglycerate dehydrogenase (PHGDH) from *E. coli* (PDB code 1YBA)^23^ as a search template. Structure determination of Ser33-NADH-PGA, Ser33-NADH-PHP, Ser33-NADH-2HG-SER and Ser33-NADH-SER complexes was carried out by molecular replacement using the crystal structure of the unliganded form of Ser33 as a template model. The model rebuilding was carried out with Refmac5^85^ as implemented in the CCP4 suite (version 7.1.018)^86^ in alternating cycles. The final manual building was performed with Coot^87^ and refinement with phenix.refine^88^. The structure was validated by MolProbity^89^. Data collection and refinement statistics are presented in Supplementary Table 2. The atomic coordinates and structure factors were deposited in the Protein Data Bank, accession codes 8PIN (Ser33-NADH), 8PIR (Ser33-NADH-PGA), 8PIO (Ser33-NADH-PHP), 8Q2I (Ser33-NADH-2HG-SER) and 8PIS (Ser33-NADH-SER). Molecular graphics and structural analyses, including structure-based sequence alignment (Fig. 4) were performed with the UCSF Chimera and ChimeraX packages^90,91^.

### Enzymatic activity assays

The transhydrogenase activity of Ser33 and its variants was assessed in a reaction mixture containing 25 mM HEPES pH 7.1, 50 µM PGA, 25 µM αKG, 500 µM NAD⁺, 250 µM glutamic acid, 1 mM dithiothreitol, 1 mM MgCl₂, 5 µM pyridoxal 5′-phosphate, and 100 µg/ml of PSAT (coupling enzyme). The reaction was initiated by the addition of Ser33 or its variants (final concentration: 40 µg/ml) and incubated at 30 °C for 10 min. To terminate the reaction, heat inactivation was performed at 95 °C for 5 min. The samples were filtered through a regenerated cellulose filter (0.2 µm pore size) and analyzed for 2HG and 3PSer using LC-MS (Supplementary Table 2; Supplementary Fig. 10; Source data are provided as a Source Data file). For transhydrogenase inhibition assays, L-serine was added to the reaction mixture at varying concentrations (50 µM, 100 µM, 250 µM, and 500 µM).

The αKG reductase activity of Ser33 and its variants was evaluated in a reaction mixture containing 25 mM Tris-HCl pH 7.5, 500 µM αKG, 500 µM NADH, and 1 mM dithiothreitol. The reaction was initiated by adding Ser33 or its variants at final concentrations of 60 µg ml⁻¹ and 120 µg ml⁻¹, respectively, followed by incubation at 30 °C for 20 min. NADH consumption was monitored spectrophotometrically at 340 nm using a Tecan M200 Pro plate reader with 96-well plates. Initial velocities were calculated using an extinction coefficient for NADH of 6220 M⁻¹ cm⁻¹.

For all enzyme assays, ‘no enzyme’ and ‘no substrate’ control reactions were performed. For the αKG reductase activity, the control without substrate was used for background correction. For the transhydrogenase activity, no product was detected in the control reactions. Statistical analyses and graphical representations were performed using GraphPad Prism (version 10.0.0).

### LC-MS analysis

Intermediates of the de novo serine synthesis pathway (3PGA, 2HG, 3PSer, and αKG) were measured and quantified using a targeted LC-MS/MS method on a Shimadzu Nexera XR LC 20AD coupled to a Sciex API4000 Qtrap mass spectrometer equipped with an electrospray ion source. The formation of 2HG and 3PSer was used as a readout of transhydrogenase activity (Supplementary Fig. 10). Filtered samples (30 µl) were mixed with an equal volume of the internal standard, ^13^C-labeled αKG, at a final concentration of 60 µM. Metabolite separation was achieved using reverse-phase liquid chromatography on an EVO C18 column (Phenomenex Kinetex, 2.6 µm, 100 Å, 150 × 2.1 mm). The column temperature was maintained at 55 °C, with a flow rate of 0.2 ml min⁻¹. An isocratic elution was performed with 100% solvent A (water containing 0.5 % formic acid) to elute the analytes, followed by a washing step with 100% solvent B (methanol containing 0.5% formic acid) and column regeneration under starting conditions. Detection was carried out in negative ionization mode using multiple reaction monitoring (MRM). Chromatographic gradient details, MS instrument parameters, and specific mass transitions are provided in Supplementary Table 2. Measurement values were normalized to the ^13^C-labeled internal standard. Absolute concentrations were calculated based on external calibration curves.

### Circular dichroism

Spectra were acquired in a J-815 CD spectropolarimeter (Jasco Corp., Tokyo, Japan) equipped with a water-cooled Peltier by using Hellma 110-QS quartz cuvettes with a 1-mm optical path. The thermal dependence of ellipticity was monitored in the range from 25 °C to 80 °C at 222 nm. Temperature was increased stepwise by 1 °C min^−1^. Ser33 was assayed at 8 µM in 25 mM Tris-HCl pH 7.5 and 75 mM NaCl. The ligands 3PGA, 2HG, αKG, NADH, PHP and L-Serine were added at 5 mM each (Source data are provided as a Source Data file).

### Molecular dynamics simulations

We used the crystal structure of Ser33 enzyme in complex with L-serine and NADH as the starting structure for the molecular dynamics simulations (PDB entry: 8PIS). The structure was subsequently modified according to each simulation setup, by either retaining or removing the serine residues as appropriate. The simulations were carried out with AMBER 22 package^92^ implemented with ff14SB^93^ and gaff2^94^ force fields. The system was neutralized by adding explicit counter ions. Each complex was immersed in a water box with a 10 Å buffer of TIP3P water molecules^95^. A two-stage geometry optimization approach was performed. The first stage minimizes only the positions of solvent molecules, and the second stage is an unrestrained minimization of all the atoms in the simulation cell. The systems were then gently heated by incrementing the temperature from 0 to 300 K under the constant pressure of 1 atm and periodic boundary conditions. Harmonic restraints of 30 kcal mol^−1^ were applied to the solute, and the Andersen temperature coupling scheme was used to control and equalize the temperature. The time step was kept at 1 fs during the heating stages, allowing potential inhomogeneities to self-adjust. Long-range electrostatic effects were modeled using the particle-mesh-Ewald method^96^. An 8 Å cut-off was applied to Lennard-Jones interactions. Each system was equilibrated for 2 ns with a 2-fs time step at a constant volume and temperature of 300 K. A single long-timescale (1 µs) simulation was performed per condition to capture the relevant conformational dynamics. Dynamic cross-correlation maps (DCCMs) and root mean square fluctuation (RMSF) analyses were performed on the molecular dynamics trajectories using the cpptraj module included in AMBER22.

### Type II PHGDH sequence collection and alignment

Type II PHGDH sequences were identified through targeted UniProt text searches and NCBI BLASTp queries against specific taxonomic IDs, using *S. cerevisiae* Ser33 (UniProt code P40054) as a reference, combined with EC number filtering (EC 1.1.1.95) and verification of the Type II-specific CXC motif. Sequences with incomplete N-termini, ambiguous annotations, or missing motifs were excluded. The final dataset included representative bacterial orthologs as prokaryotic outgroups and fungal sequences from Ascomycota, Basidiomycota, and Chytridiomycota. To accurately model the large insertion/deletion event represented by the N-terminal extension, sequences were aligned using PRANK (v.170427), a phylogeny-aware alignment algorithm. Alignments were not trimmed to preserve evolutionary information contained in gap patterns. Ancestral sequences were inferred as part of the PRANK workflow.

### Phylogenetic analysis

Maximum-likelihood phylogenetic trees were reconstructed using IQ-TREE (v3.0.1) with ModelFinder Plus (-m MFP command) for automatic substitution model selection. Tree robustness was assessed using 1000 ultrafast bootstrap (UFBoot) replicates. Trees were visualized and annotated using FigTree (v1.4.4), with taxonomic groups and N-terminal extension presence highlighted.

### Phylogenetic signal and extension length analysis

N-terminal extension lengths were measured for all fungal sequences. Phylogenetic signal was assessed using Pagel’s λ implemented in the R package *phytools*. Statistical analyses, including Z-score-based outlier detection and one-way ANOVA across taxonomic groups, were performed in R (v4.5.1). Conservation scores were calculated using the AMAS algorithm via the AACon web service and visualized in Jalview. Functionally important residues and putative phosphorylation sites identified in the *S. cerevisiae* Ser33 sequence were mapped onto the alignment using custom annotation files to assess their evolutionary conservation across all included species.

### Reporting summary

Further information on research design is available in the Nature Portfolio Reporting Summary linked to this article.

## Supplementary information

Supplementary Information

Reporting Summary

Transparent Peer Review file

## Source data

Source Data

## Data Availability

The atomic coordinates and structure factors have been deposited in the Protein Data Bank (PDB) under the accession codes 8PIN (Ser33-NADH), 8PIR (Ser33-NADH-PGA), 8PIO (Ser33-NADH-PHP), 8Q2I (Ser33-NADH-2HG-SER), and 8PIS (Ser33-NADH-SER). MD simulations data are provided on Zenodo 10.5281/zenodo.19468609. The PDB code of the previously published structure used in this study is 1YBA. All other datasets generated or analyzed during this study are available from the corresponding authors upon request. Source Data is provided as a Source Data file. Source data are provided with this paper.
