# Supplementary material for: Molecular mechanisms of transhydrogenase activity and allosteric regulation in eukaryotic type II PHGDH Ser33
Source: Nat Commun. 2026 Jun 19;17:7756. doi: 10.1038/s41467-026-74211-9 (PMC13434204; doi:10.1038/s41467-026-74211-9)
Supplement: Supplementary file 1 [file 41467_2026_74211_MOESM1_ESM.pdf]

SUPPLEMENTARY INFORMATION

Molecular mechanisms of transhydrogenase activity and allosteric regulation in eukaryotic Type II PHGDH Ser33

Sebastian Perrone,[1,2,3,#] Javier O. Cifuente,[4,#] 'Deceased: Leonardo Mastrella',[1,3] Alberto Marina,[2] Beatriz Trastoy,[5,6] Julia Becker-Kettern,[3] Jean-François Conrotte,[3] Adrià Alcaide-Jiménez[7], Francisco Corzana,[8] Enrico Glaab[3], Marcelo E. Guerin[1,2,6,7,*], Carole L. Linster[3,*]

[1] Structural Glycobiology Laboratory, Biobizkaia Health Research Institute, Cruces University Hospital, 48903 Barakaldo, Bizkaia, Spain.

[2] Structural Glycobiology Laboratory, Center for Cooperative Research in Biosciences (CIC bioGUNE), Basque Research and Technology Alliance (BRTA), Bizkaia Technology Park, Building 801A, 48160, Derio, Spain.

[3] Luxembourg Centre for Systems Biomedicine, University of Luxembourg, L-4362 Esch-sur-Alzette, Luxembourg.

[4] Instituto Biofisika (UPV/EHU, CSIC), University of the Basque Country, E-48940, Leioa, Spain.

[5] Structural Glycoimmunology Laboratory, Biobizkaia Health Research Institute, Cruces University Hospital, 48903 Barakaldo, Bizkaia, Spain.

[6] Ikerbasque, Basque Foundation for Science, 48009 Bilbao, Spain.

[7] Structural Glycobiology Laboratory, Department of Structural and Molecular Biology; Molecular Biology Institute of Barcelona (IBMB), Spanish National Research Council (CSIC), Barcelona Science Park, c/Baldiri Reixac 4-8, Tower R, 08028 Barcelona, Catalonia, Spain.

[8] Departamento Química and Instituto de Investigación en Química de la Universidad de La Rioja (IQUR), Universidad de La Rioja, 26006 Logroño, Spain

[#]These authors contributed equally to this work

*Corresponding authors: Marcelo E. Guerin, Structural Glycobiology Laboratory, Molecular Biology Institute of Barcelona (IBMB), Spanish National Research Council (CSIC), 08028 Barcelona, Catalonia, Spain, e-mail: mrccri@ibmb.csic.es; Carole L. Linster, Luxembourg Centre for Systems Biomedicine, University of Luxembourg, L-4362 Esch-sur-Alzette, Luxembourg, e-mail: carole.linster@uni.lu.

Table of Contents

1. Supplementary Figures
Supplementary Figure 1. Production of full-length recombinant *S. cerevisiae* Ser33.
Supplementary Figure 2. Electron density maps of the refined Ser33 X-ray crystal structures.
Supplementary Figure 3. Ser33 electron density maps
Supplementary Figure 4. Ser33-ligands interactions in the Ser33-NADH complex.
Supplementary Figure 5. Ser33-ligands interactions in the Ser33-NADH-PGA complex.
Supplementary Figure 6. Ser33-ligands interactions in the Ser33-NADH-PHP complex.
Supplementary Figure 7. Ser33-ligands interactions in the Ser33-NADH-2HG-SER complex.
Supplementary Figure 8. Ser33-ligands interactions in the Ser33-NADH-SER complex.
Supplementary Figure 9. Proposed mechanism of the first half-reaction catalyzed by the Ser33 transhydrogenase.
Supplementary Figure 10. LC-MS/MS detection of 3PSer, 3PGA, 2HG, and αKG.
Supplementary Figure 11. NAD^+/αKG-dependency of the transhydrogenase activity.
Supplementary Figure 12. Localization of Ser33 variants.
Supplementary Figure 13. Structural comparison of NADH binding sites in PHGDHs.
Supplementary Figure 14. αKG reductase activity of wild-type Ser33 and selected Ser33 variants.
Supplementary Figure 15. L-serine effect on Ser33 transhydrogenase activity.
Supplementary Figure 16. Phylogenetic distribution of Type II PHGDH across Fungi and Prokaryotes.
Supplementary Figure 17. Structural comparison between Ser33 and human PHGDH.

2. Supplementary Tables
Supplementary Table 1. X-ray data collection and refinement statistics.
Supplementary Table 2. Targeted LC-MS method for quantitative measurements of serine synthesis pathway intermediates.

3. Supplementary References

1. Supplementary Figures

a

MHHHHHHENLYFQGSGAMSYSAADNLQDSFQRAMNFSGSPGAVSTSPTQSFMNTLPRRVSITKQPKALKP
FSTGDMNILLLENVNATAIKIFKDQGYQVEFHKSSLPEDELIEKIKDVHAIGIRSKTRLTEKILQHARNLVCIGCFC
IGTNQVDLKYAASKGIAVFNSPFSNSRSVAELVIGEIISLARQLGDRSIELHTGTWNKVAARCWEVRGKTLGIIG
YGHIGSQLSVLAEAMGLHVLYYDIVTIMALGTARQVSTLDELLNKSDFVTLHVPATPETEKMLSAPQFAAMKD
GAYVINASRGTVVDIPSLIQAVKANKIAGAALDVYPHEPAKNGEGSFNDELNSWTSELVSLPNIILTPHIGGSTE
EAQSSIGIEVATALSKYINEGNSVGSVNFPEVSLKSLDYDQENTVRVLYIHRNVPGVLKTVNDILSDHNIEKQFS
DSHGEIAYLMADISSVNQSEIKDIYEKLNQTSAKVSIRLLY

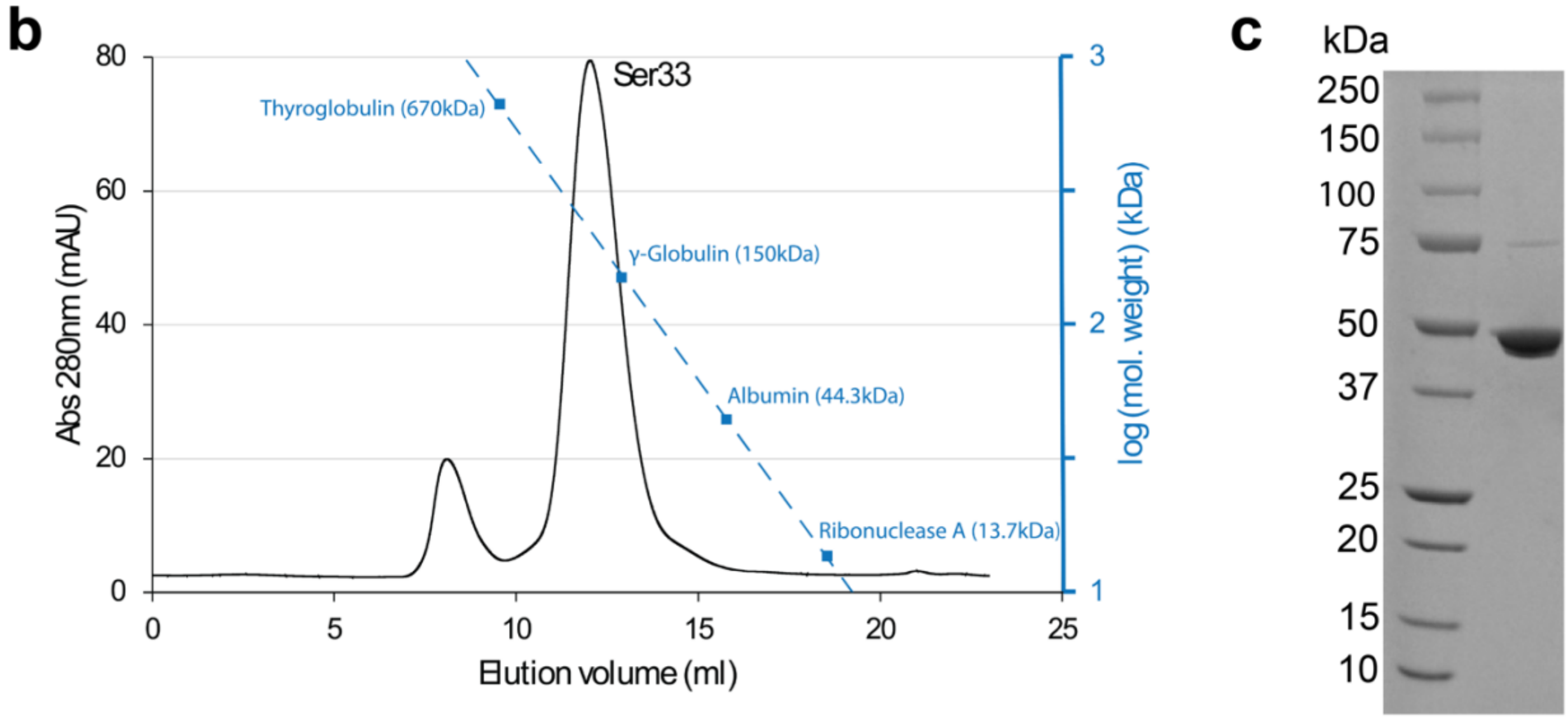

Supplementary Figure 1 | Production of full-length recombinant *S. cerevisiae* Ser33. a The Ser33 amino acid sequence is highlighted in yellow. The His$_6$ tag, TEV protease cleavage site, and a short linker sequence (GSGA) are highlighted in gray, orange, and red, respectively. **b** Elution profile of recombinant Ser33 protein from a Superdex 200 Increase 10/300 GL size exclusion column, following nickel affinity purification —expected MW of the tetramer: 206 kDa. **c** SDS-PAGE analysis with Coomassie staining of purified recombinant wild-type Ser33 protein —expected MW of the protomer: 51.5 kDa (Source data are provided as a Source Data file).

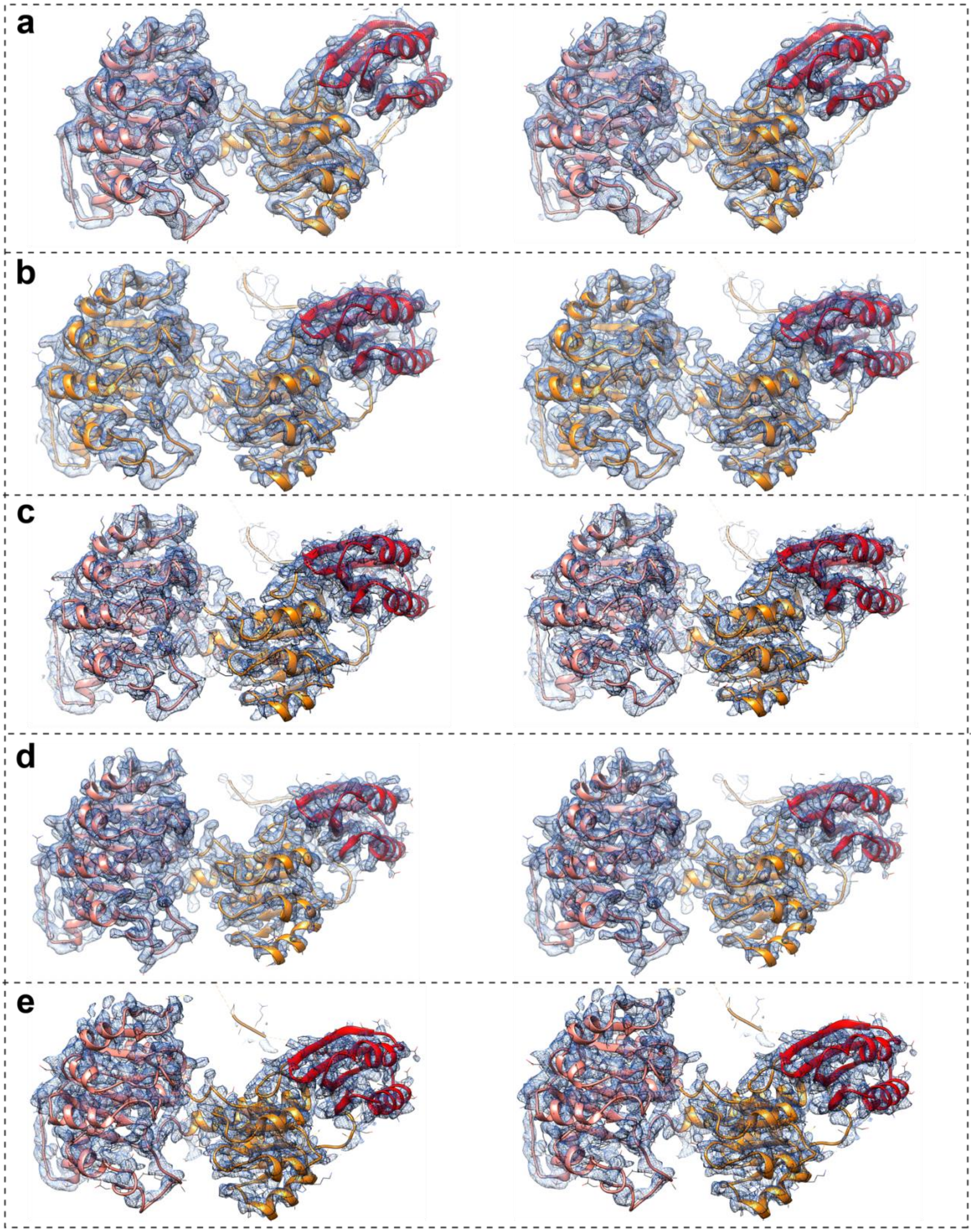

Supplementary Figure 2 | Electron density maps of the refined Ser33 X-ray crystal structures. Stereo views of the final electron density maps (2mFo-DFc contoured at 1σ) of **a** Ser33-NADH (PDB code 8PIN), **b** Ser33-NADH-PGA (PDB code 8PIR), **c** Ser33-NADH-PHP (PDB code 8PIO), **d** Ser33-NADH-SER (PDB code 8PIS) and **e** Ser33-NADH-2HG-SER (PDB code 8Q2I).

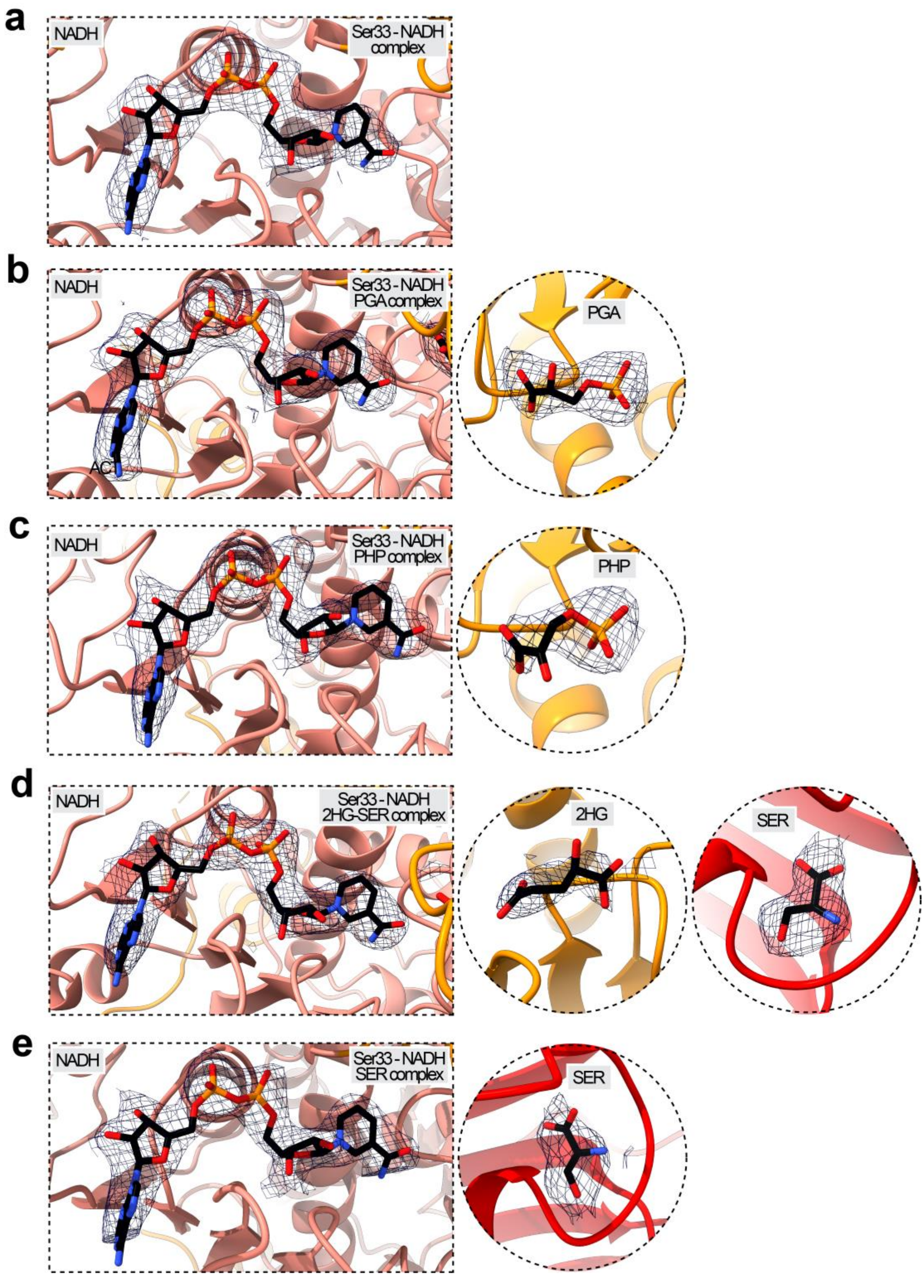

Supplementary Figure 3 | Electron density maps of ligands observed in the **a** Ser33-NADH, **b** Ser33-NADH-PGA, **c** Ser33-NADH-PHP, **d** Ser33-NADH-2HG-SER, and **e** Ser33-NADH-SER complexes.

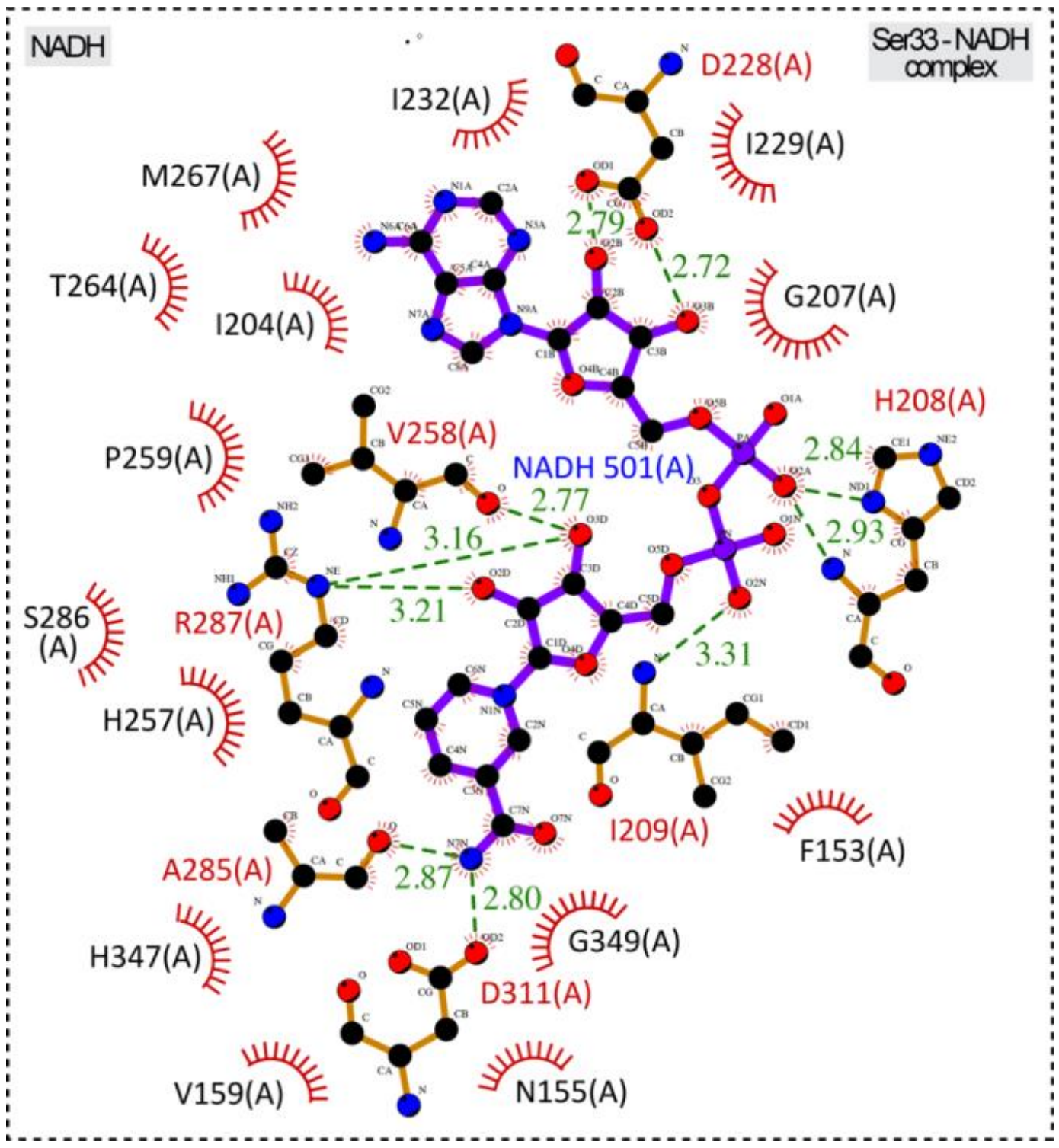

Supplementary Figure 4 | Ser33-ligands interactions in the Ser33-NADH complex. 2D representation of Ser33 interactions with NADH in the Ser33-NADH complex, generated with Ligplot+.[1]

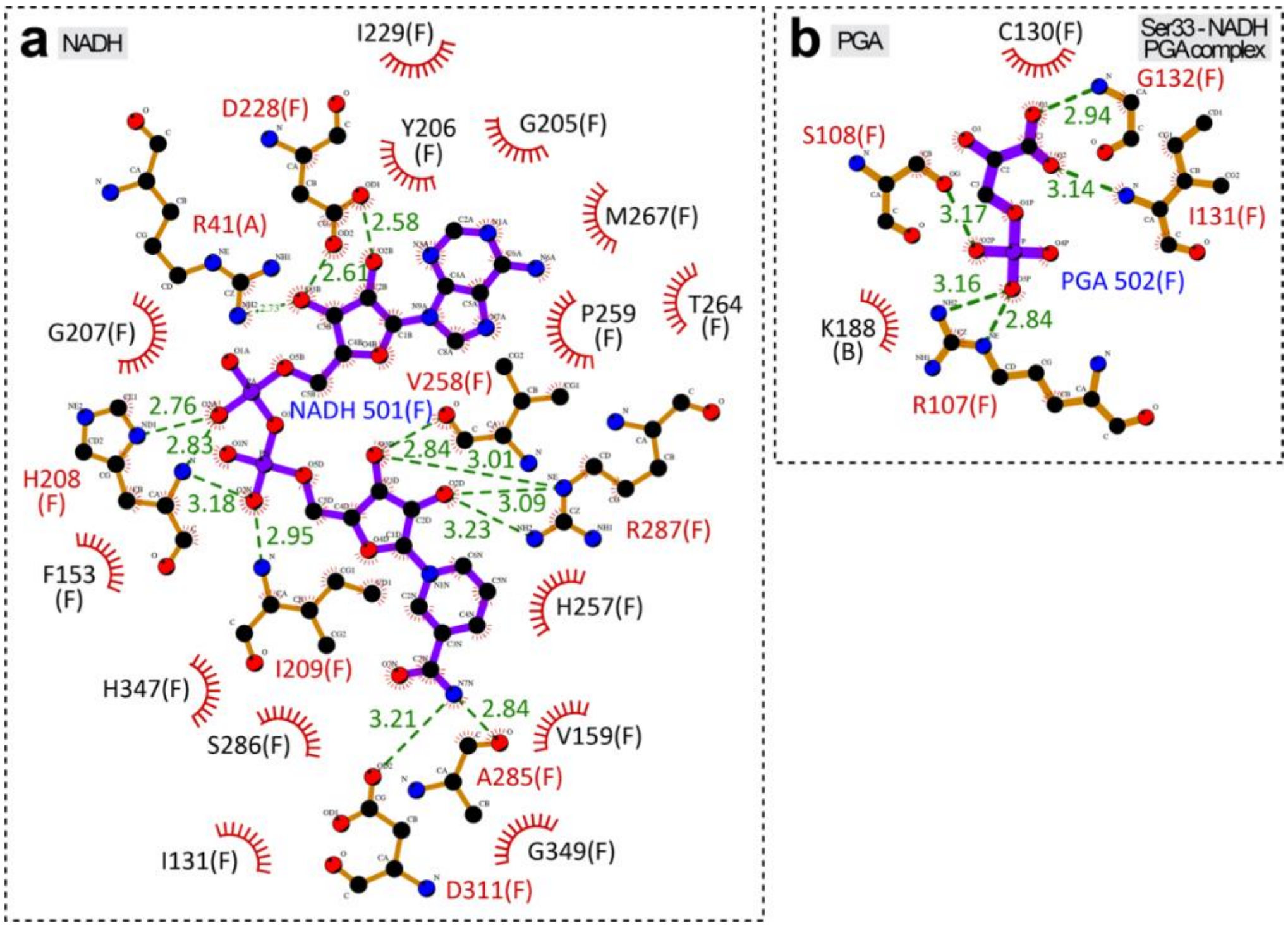

Supplementary Figure 5 | Ser33-ligands interactions in the Ser33-NADH-PGA complex. 2D representation of Ser33 interactions with NADH (**a**) and PGA (**b**) in the Ser33-NADH-PGA complex, generated with Ligplot.[1]

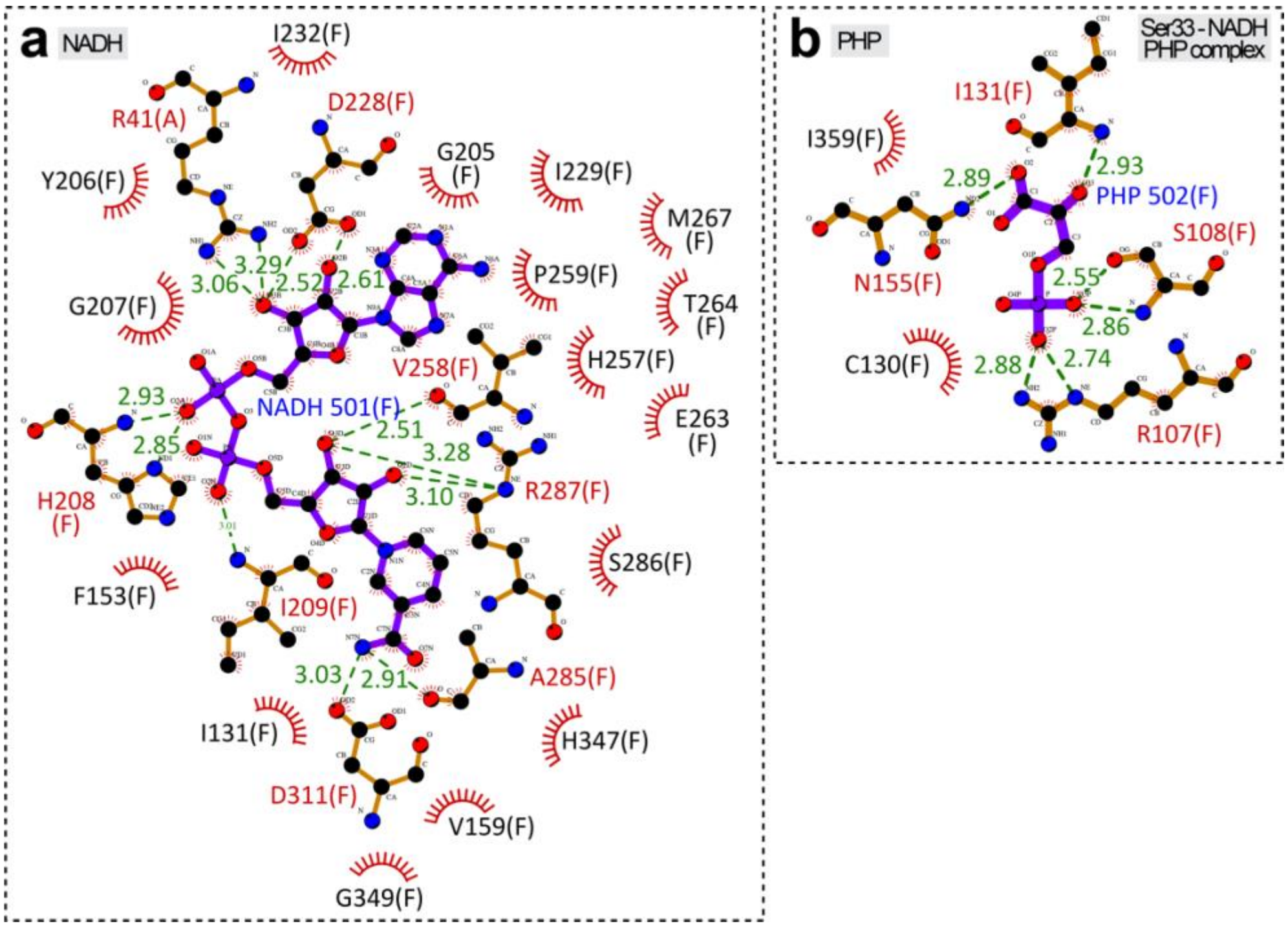

Supplementary Figure 6 | Ser33-ligands interactions in the Ser33-NADH-PHP complex. 2D representation of Ser33 interactions with NADH (**a**) and PHP (**b**) in the Ser33-NADH-PHP complex, generated with Ligplot+.[1]

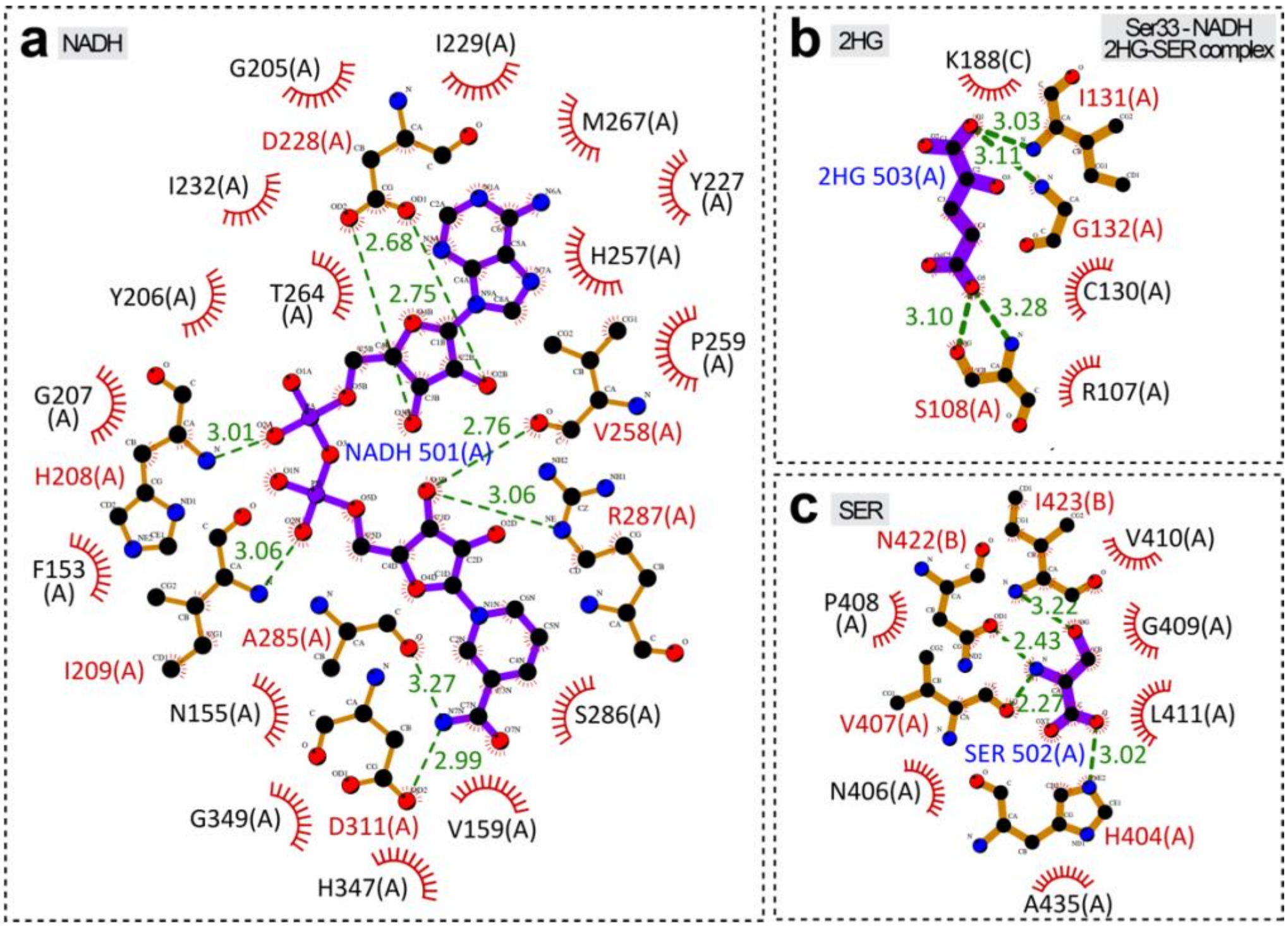

Supplementary Figure 7 | Ser33-ligands interactions in the Ser33-NADH-2HG-SER complex. 2D representation of Ser33 interactions with NADH (**a**), 2HG (**b**) and SER (**c**) in the Ser33-NADH-2HG-SER complex, generated with Ligplot+.[1]

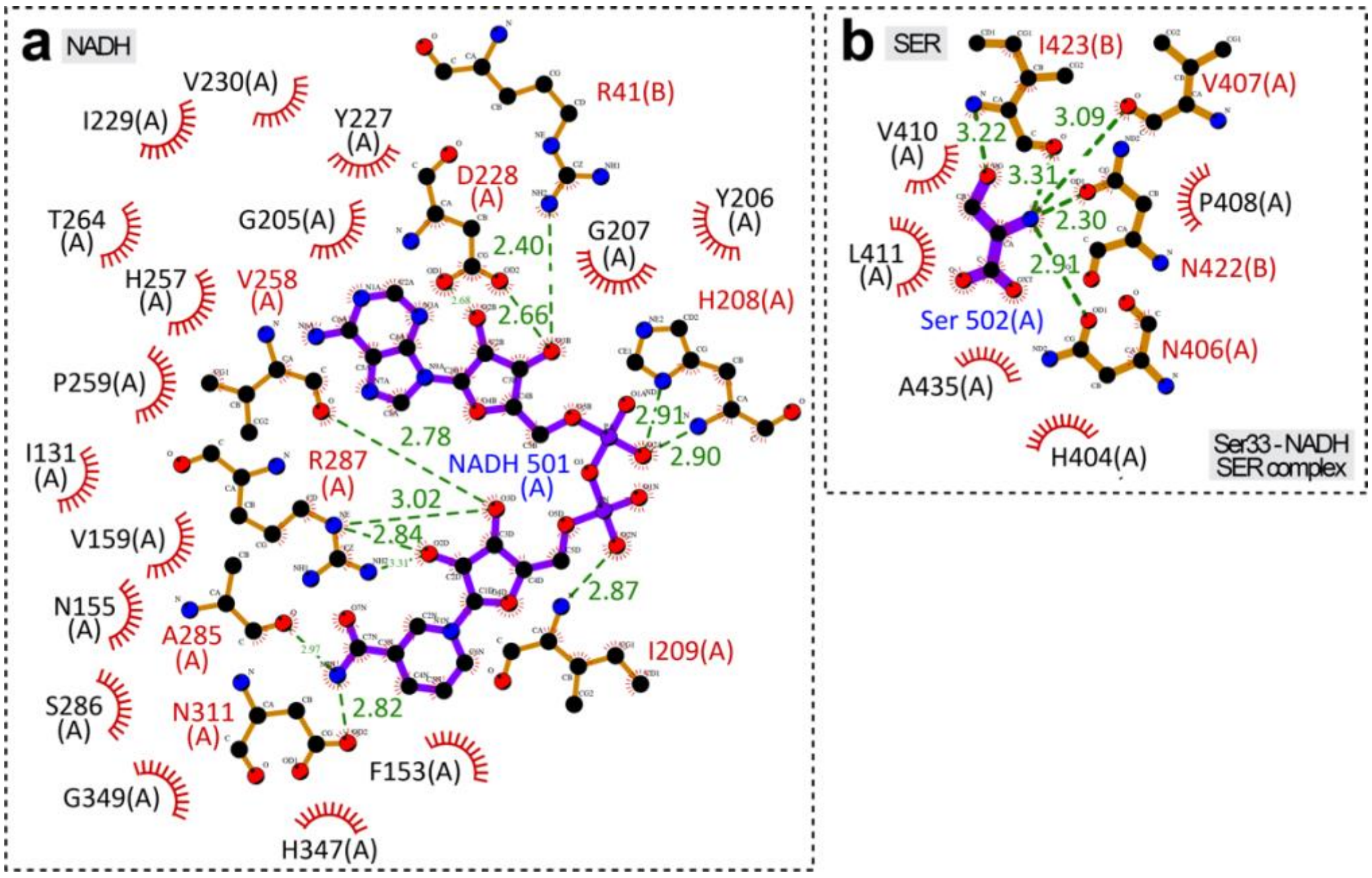

Supplementary Figure 8 | Ser33-ligands interactions in the Ser33-NADH-SER complex. 2D representation of Ser33 interactions with NADH (**a**) and SER (**b**) in the Ser33-NADH-SER complex, generated with Ligplot+.[1]

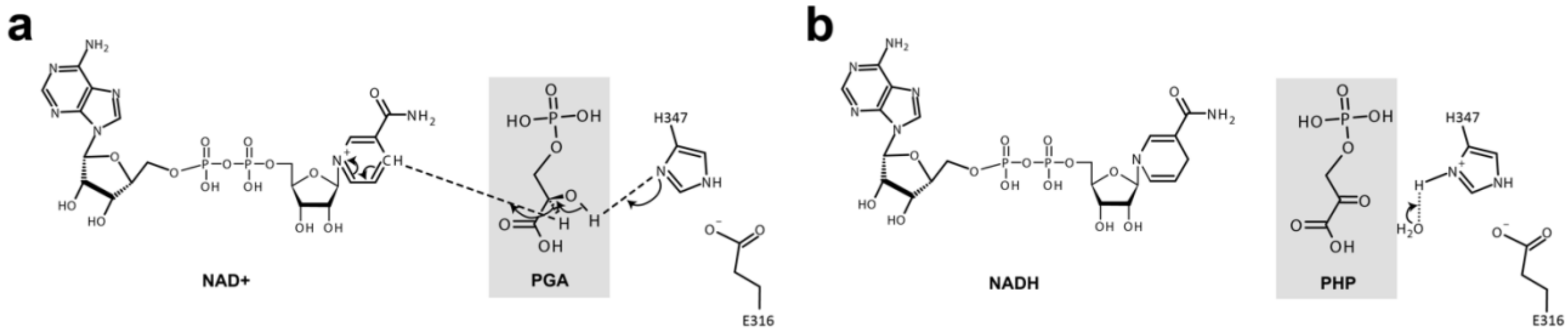

Supplementary Figure 9 | Proposed mechanism of the first half-reaction catalyzed by the Ser33 transhydrogenase. a The first step consists of a committed step, where the catalytic histidine H347 deprotonates the hydroxyl group in βC of PGA, a process facilitated by glutamic acid E316. A hydride is transferred to the positively charged nicotinamide ring in an aromatic bimolecular nucleophilic addition. **b** The catalytic H347 is deprotonated by a hydroxide ion derived from water, regenerating the enzyme and forming the products, NADH and PHP.

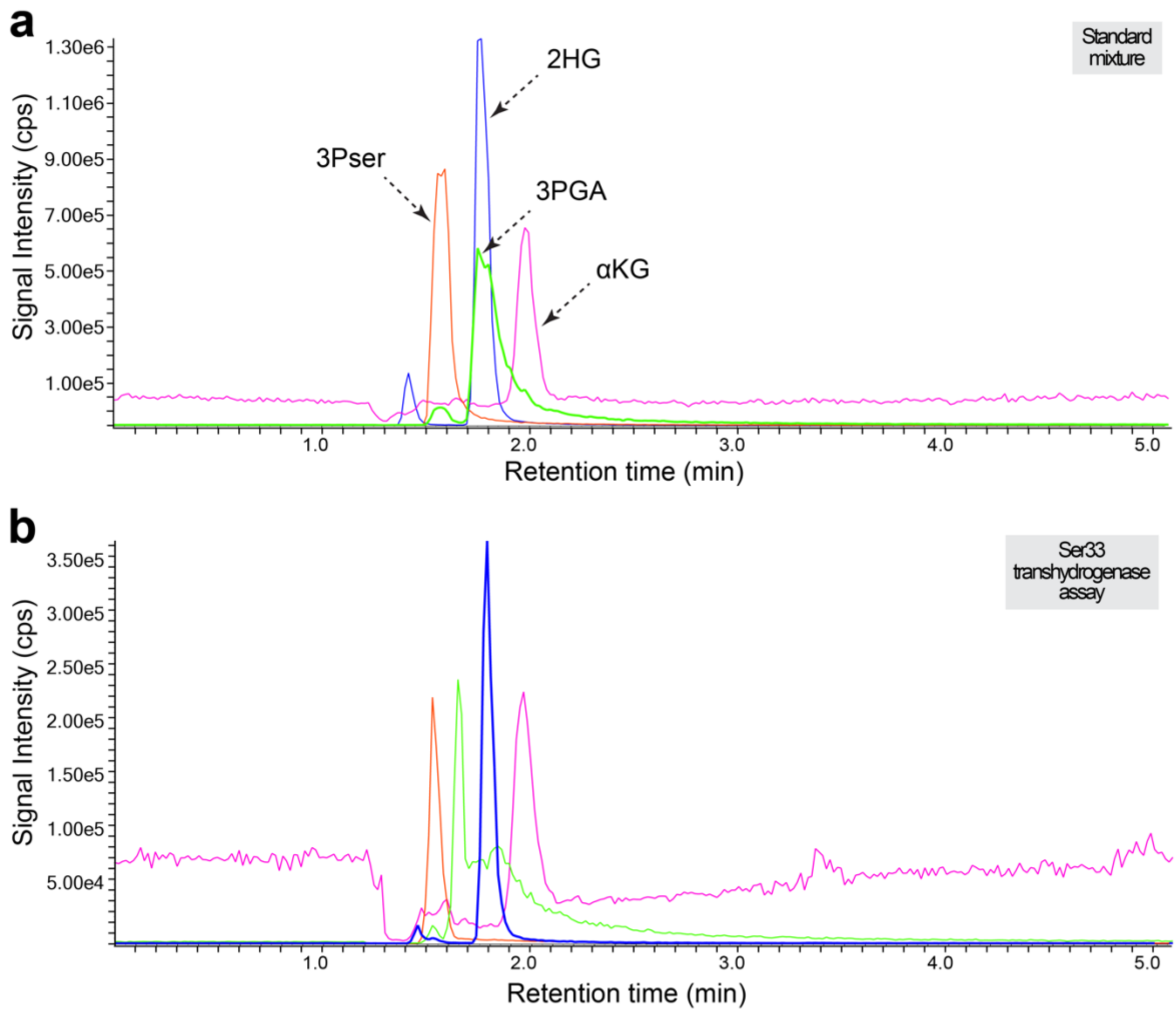

Supplementary Figure 10 | LC-MS/MS detection of 3PSer, 3PGA, 2HG, and αKG. Representative chromatograms for a standard mixture containing 3PSer, 3PGA, 2HG, and αKG (**a**) and a Ser33 transhydrogenase assay sample (**b**) analyzed using the targeted LC-MS/MS method described in this study. Metabolites were identified based on their retention times and specific m/z transitions: 3PSer (m/z 183.8 → 96.7), 3PGA (m/z 184.8 → 96.7), 2HG (m/z 146.8 → 128.8), αKG (m/z 144.7 → 100.9).

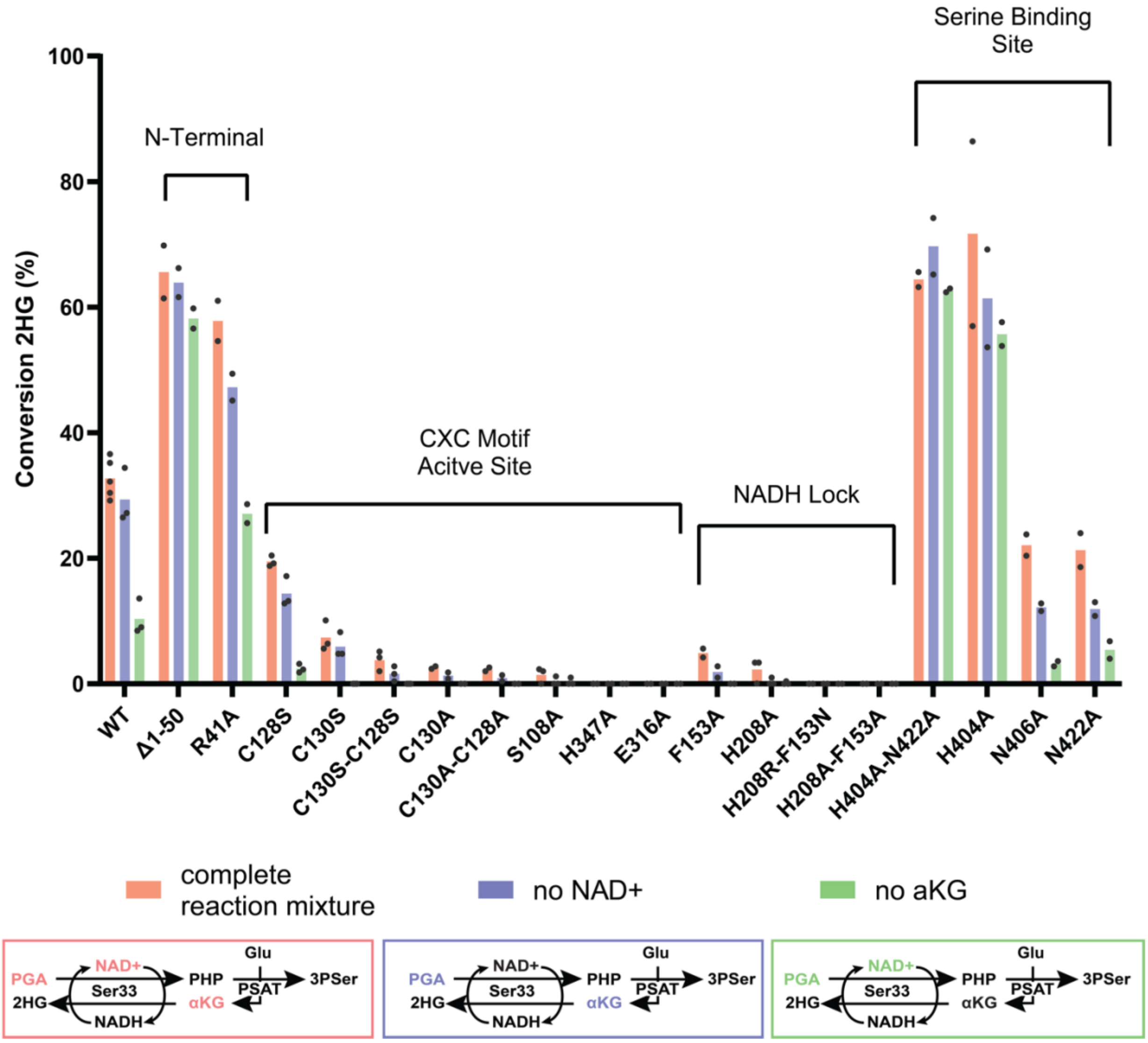

Supplementary Figure 11 | αKG-dependency of the transhydrogenase activity. Transhydrogenase activities measured for wild-type and variant Ser33 proteins in this study, in a complete reaction mixture (orange, identical to data shown in main Figure 3a), in a mixture lacking NAD^+ (light blue), and in a mixture lacking αKG (light green). Data are means n = 2-5 independent replicates shown as dots. (Source data are provided as a Source Data file).

a

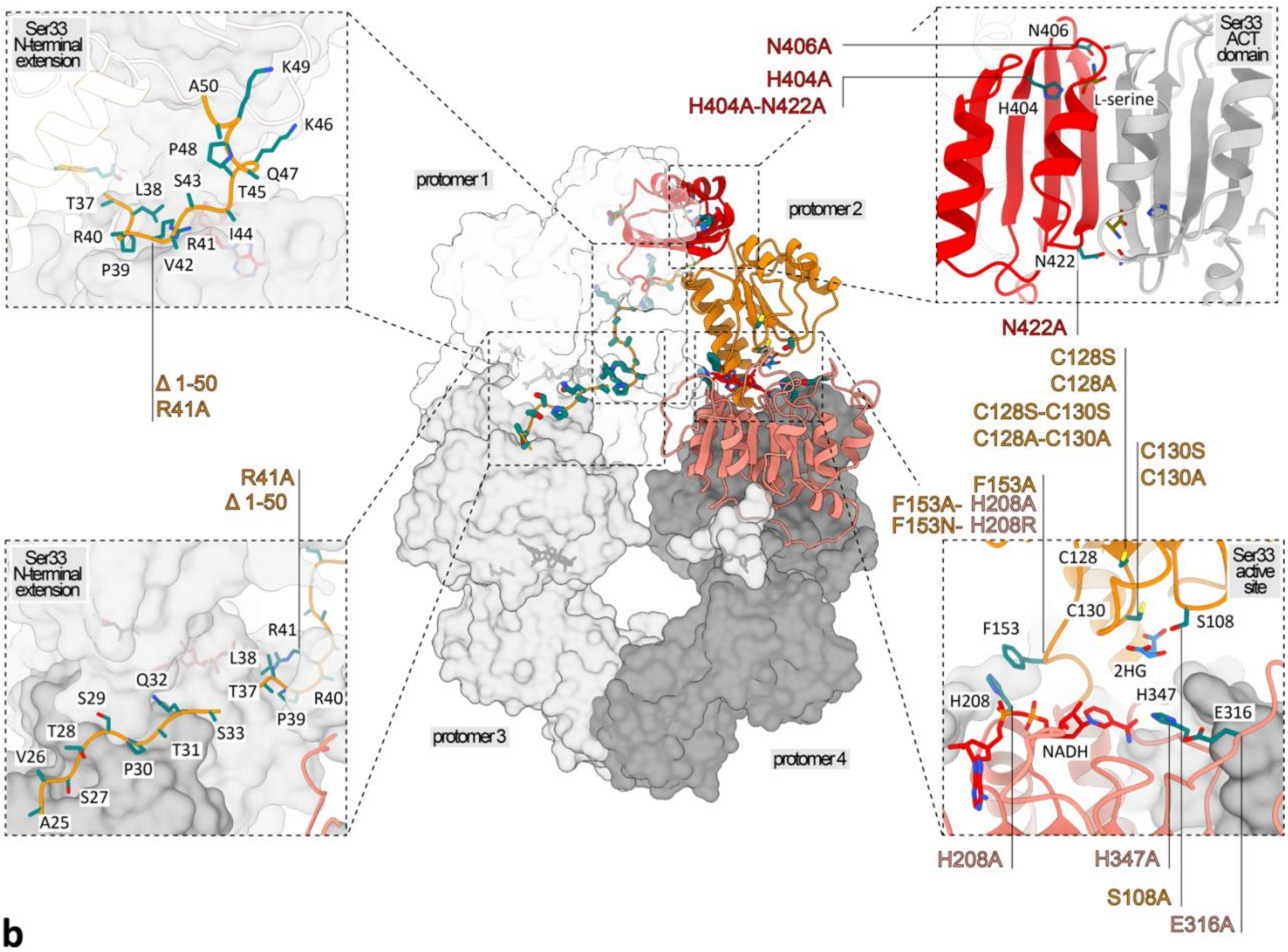

b

Variant	Localization
C128S	CXC motif
C130S	CXC motif
C128S - C130S	CXC motif
C128A	CXC motif
C130A	CXC motif
C130A - C128A	CXC motif
H347A	active Site
E316A	active Site
S108A	active Site

Variant	Localization
H208A	NADH lock
F153A	NADH lock
F153N - H208R	NADH lock
F153A - H208A	NADH lock
H404A	serine binding site
N406A	serine binding site
N422A	serine binding site
H404A - N422A	serine binding site
Δ1-50	N-terminus
R41A	N-terminus

Supplementary Figure 12 | Localization of Ser33 variants. a Cartoon and surface representations of Ser33 homotetramer highlighting the amino acid variants. **b** Description and localization of all Ser33 variants produced and analyzed in this study.

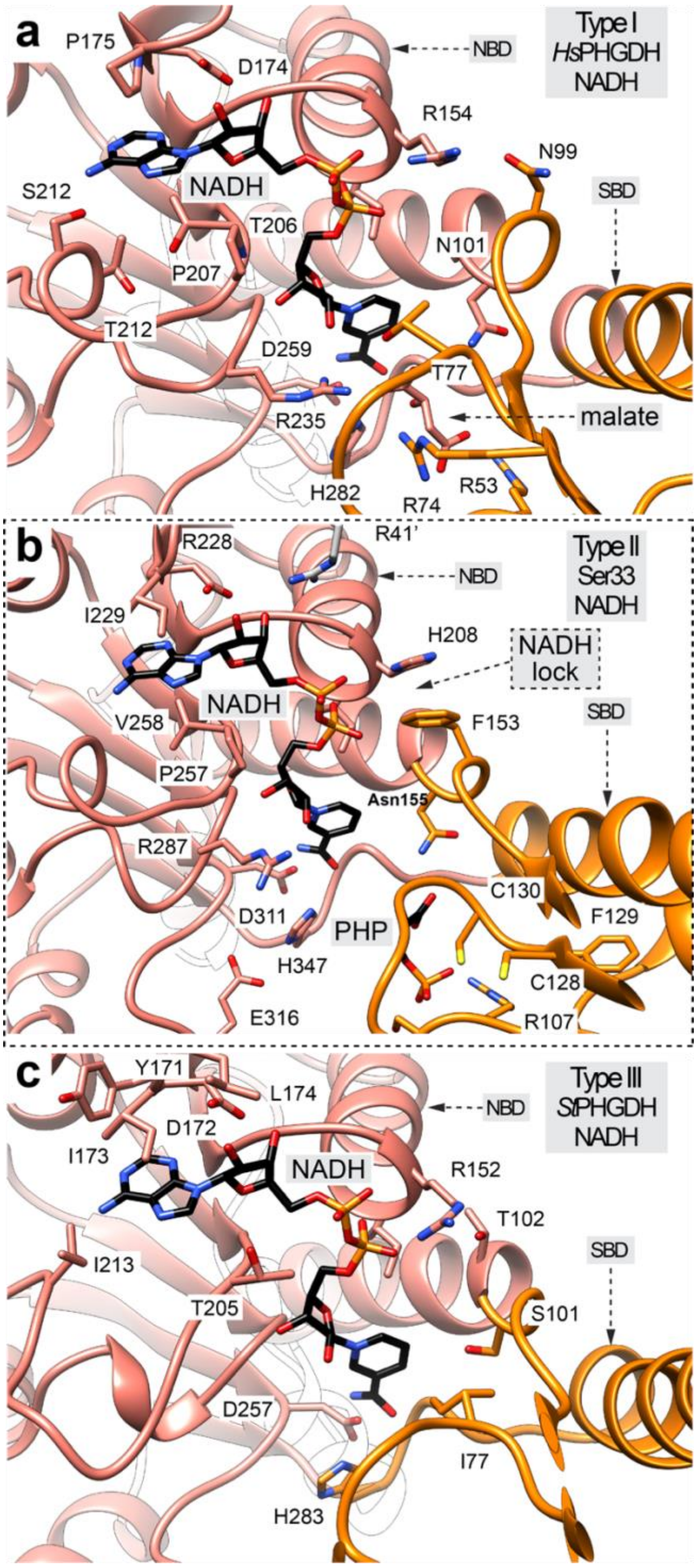

Supplementary Figure 13 | Structural comparison of NADH binding sites in PHGDHs. Close-up views of the NADH binding sites in the Type I PHGDH from *H. sapiens* (PDB code 2G76) (**a**), the Type II PHGDH (Ser33) from *S. cerevisiae* (PDB code 8PIO) (**b**), and the Type III PHGDH from *S. tokodaii* (PDB code 2EKL) (**c**).

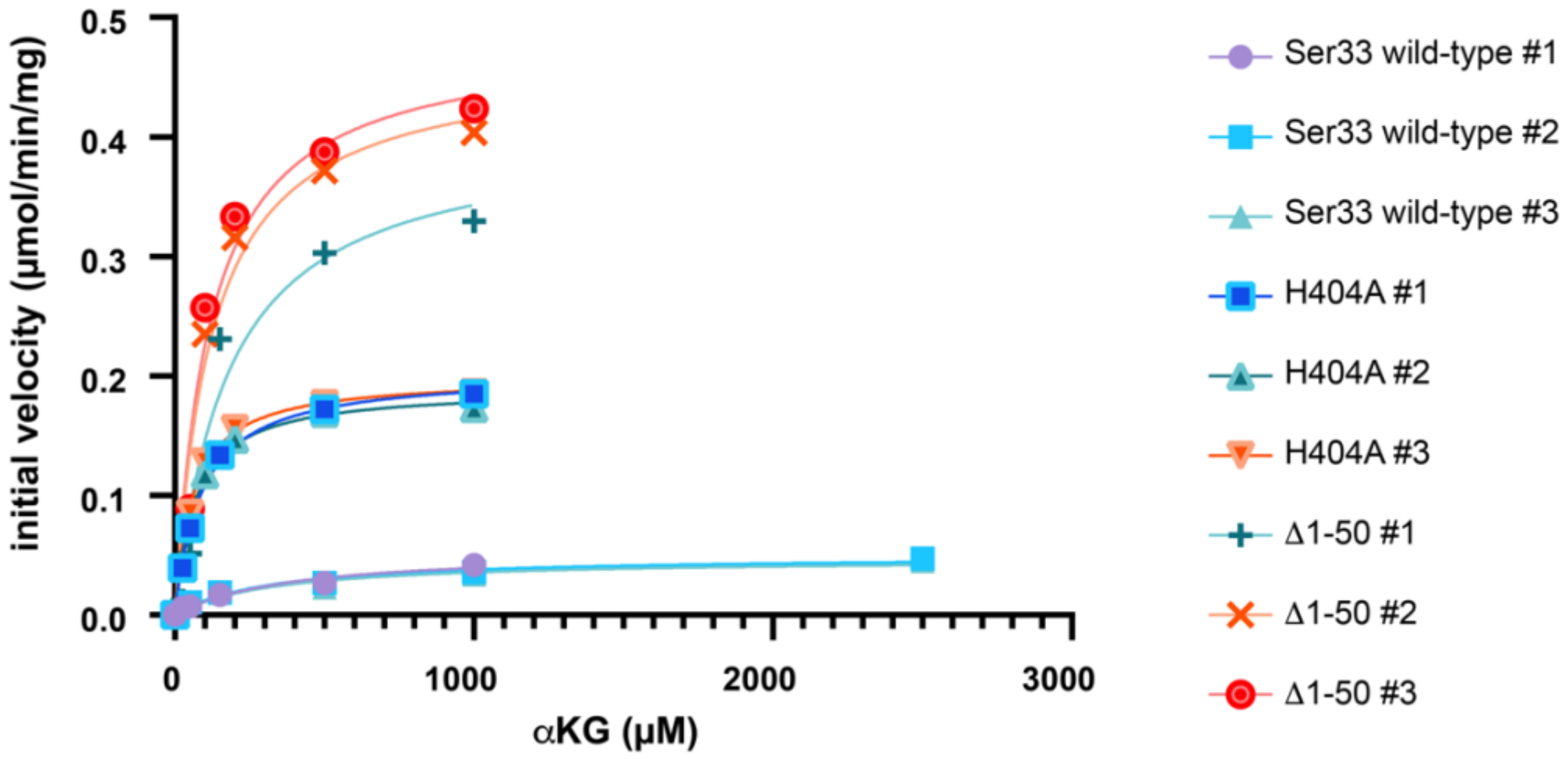

	Average αKG reductase				Std dev		
	**WT**	**H404A**	**Δ1-50**		WT	H404A	Δ1-50
**Vmax (µmol/min/mg)**	**0.050**	**0.197**	**0.451**	Vmax	0.003	0.006	0.034
**Km (µM)**	**335.5**	**71.7**	**137.8**	Km	25.1	11.7	27.9

Supplementary Figure 14 | αKG reductase activity of wild-type Ser33 and selected Ser33 variants. The spectrophotometric αKG reductase activity assays of wild-type Ser33 and selected variants contained 60 µg/ml of protein, 500 µM NADH, 1 mM DTT, and 25 mM Tris-HCl pH 7.5. Kinetics parameters were determined using GraphPad Prism (version 10.6.1). Data shown are means and SDs (n=3).

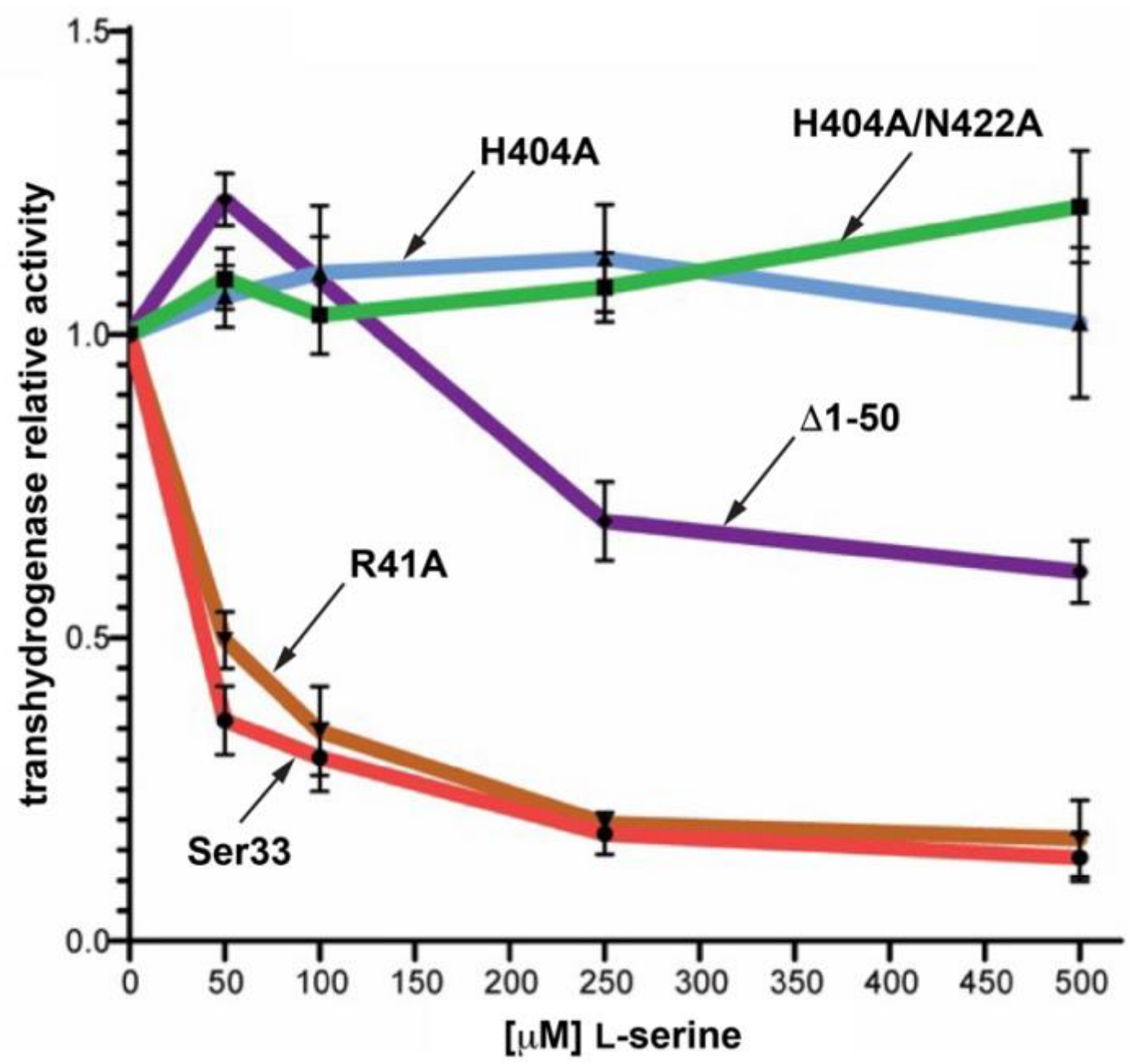

Supplementary Figure 15 | L-serine effect on Ser33 transhydrogenase activity. The effect of the indicated concentrations of L-serine on the Ser33 transhydrogenase activity was determined in the presence of 50 μM PGA. Enzymatic activity values are shown as relative values, where the activity in the absence of L-serine was considered 100%. All data are represented as means ± SDs of n = 3 independent replicates.

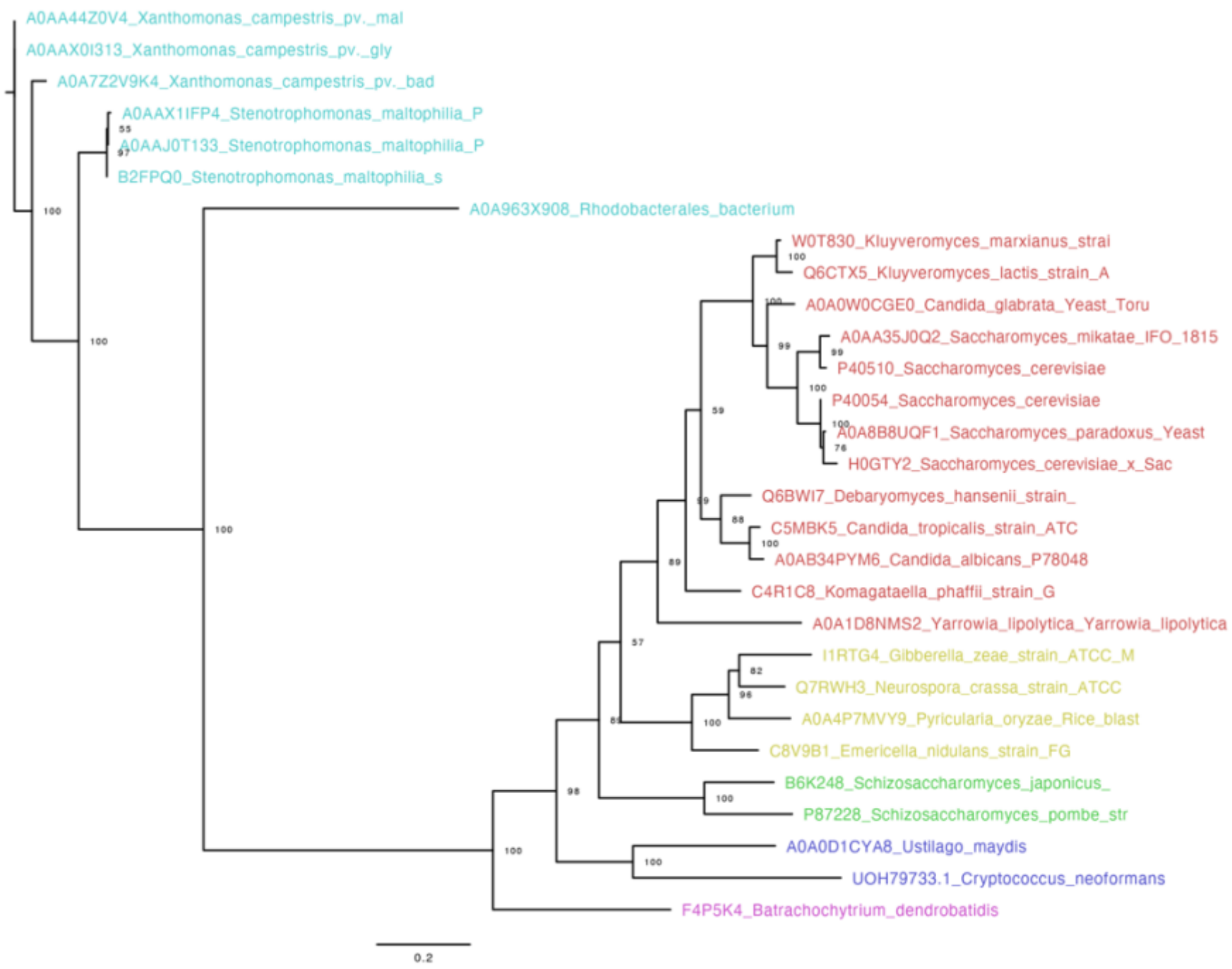

Supplementary Figure 16 | Phylogenetic distribution of Type II PHGDH across Fungi and Prokaryotes. The maximum-likelihood phylogenetic tree illustrates the evolutionary relationships between fungal Type II PHGDH sequences and prokaryotic orthologs. The tree clearly distinguishes the major fungal phyla, including Ascomycota (red/yellow/green), Basidiomycota (purple), and Chytridiomycota (magenta), from the bacterial outgroup (cyan). Branch support values represent UFBoot proportions (values ≥95 indicate strong support).

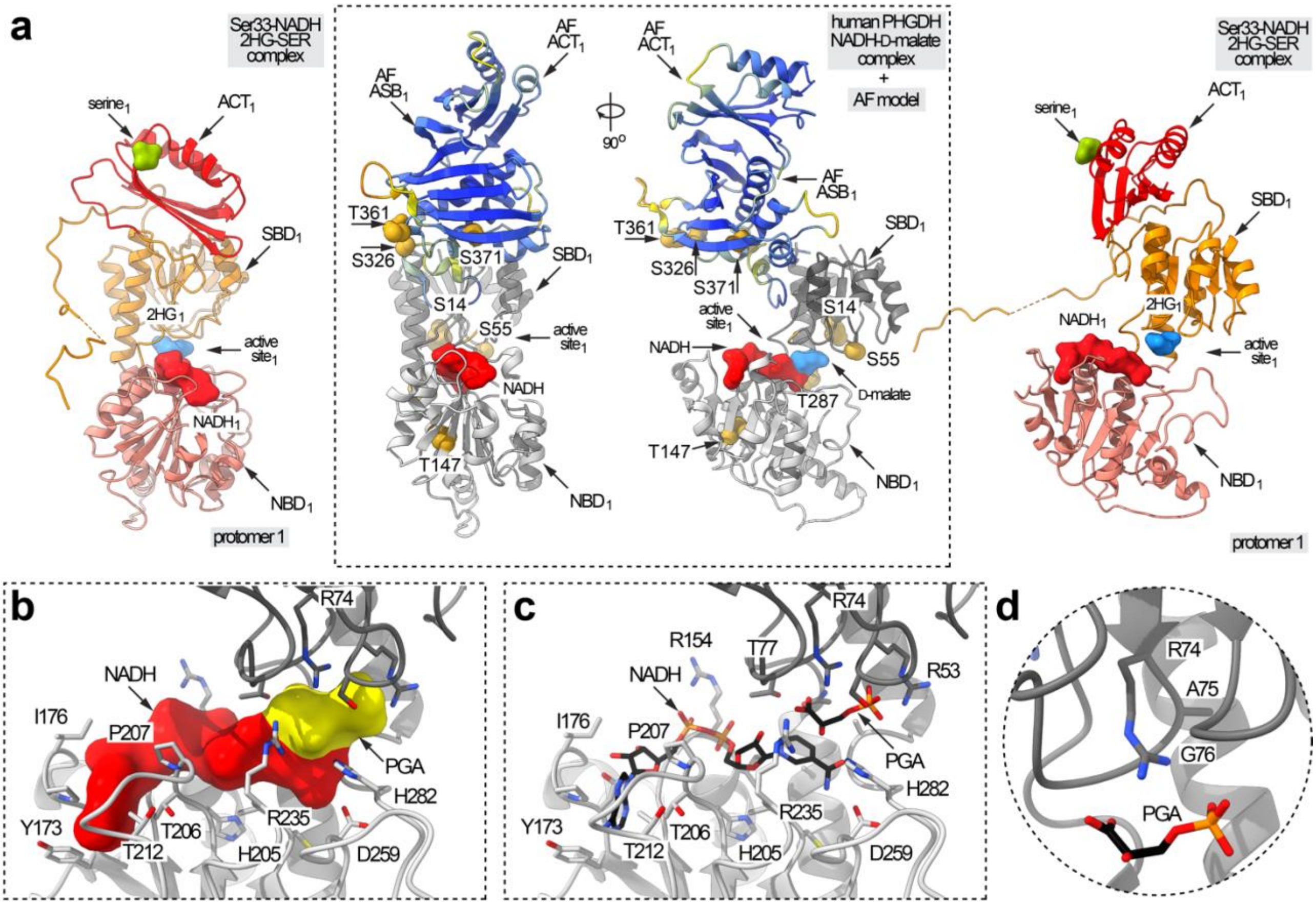

Supplementary Figure 17 | Structural comparison between Ser33 and human PHGDH homologues. **a** Two views of the cartoon representation of protomeric human PHGDH showing (i) the experimentally determined NBD and SBD domains (PDB code 2G76; *Hs*PHGDH in complex with NADH and D-malate), and (ii) the ASB and ACT domains, generated by AF. The Ser33 structure is compared as a reference. Interestingly, phosphorylation of several residues was experimentally determined, including S14, S55, T60, T78, T147, T287, S326, T361, S362, S371, S383 and S473 (yellow; PRIDE human phosphoproteome map)[2,3,4,5]. **b,c** Close-up view of the *Hs*PHGDH active site in the NADH-PGA complex (PDB code 6CWA), showing the location of NADH (red) and PGA (yellow). **d** Close-up view of the RAG motif in the *Hs*PHGDH active site.

2. Supplementary Tables

Supplementary Table 1. X-ray data collection and refinement statistics.

	Ser33_NADH	Ser33_NADH SER	Ser33_NADH PHP	Ser33_NADH PGA	Ser33_NADH 2HG_SER
**PDB code**	8PIN	8PIS	8PIO	8PIR	8Q2I
**Beamline**	ALBA BEAMLINE XALOC	Diamond	Diamond	ALBA BEAMLINE XALOC	ALBA BEAMLINE XALOC
**Wavelength**	0.9793	0.9999	0.9999	0.9793	0.9793
**Resolution Range**	46.6 - 3.19 (3.304 - 3.19)	43.11 -2.691 (2.787 - 2.691)	52.04 - 2.6 (2.693 - 2.6)	60.45 - 2.7 (2.797 - 2.7)	20.02 - 2.505 (2.595 - 2.505)
**Space Group**	P 21 21 21	P1	P1	P1	P1
**Unit Cell**	98.16 148.52 161.74 90 90 90	106.115 110.735 117.027 64.422 65.9487 67.0306	60.6 95.15 108.37 69.84 88.72 85.65	60.599 95.726 109.252 69.924 89.532 86.055	107.292 112.378 117.358 64.986 66.56 67.184
**Total reflections**	264856 (26550)	492153 (44161)	347813 (28424)	214279 (21875)	514502 (45321)
**Unique reflections**	40049 (3922)	114590 (10826)	68361 (6350)	61940 (6232)	146918 (13956)
**Multiplicity**	6.6 (6.8)	4.3 (4.1)	5.1 (4.5)	3.5 (3.5)	3.5 (3.2)
**Completeness**	99.78 (99.06)	96.97 (90.47)	97.87 (89.71)	97.73 (97.78)	97.03 (92.69)
**Mean I/sigma (I)**	25.22 (2.16)	9.57 (1.90)	9.82 (1.09)	13.01 (2.61)	10.50 (1.52)
**Wilson B-factor (Å2**	126.1	43.94	69.16	62.99	59.50
**R-merge**	0.04286 (0.8143)	0.1332 (0.3922)	0.1031 (1.424)	0.06412 (0.576)	0.075 (0.8167)
**R-meas**	0.04667 (0.8824)	0.1529 (0.4527)	0.1152 (1.606)	0.07621 (0.6817)	0.08874 (0.9889)
**CC1/2**	1 (0.829)	0.988 (0.893)	0.992 (0.604)	0.998 (0.905)	0.04689 (0.547)
**CC***	1 (0.952)	0.997 (0.971)	0.998 (0.868)	0.999 (0.975)	0.997 (0.65)
**Refinement**					0.999 (0.888)
**Reflections used in refinement**	40035 (3919)	114027 (10714)	68212 (6228)	61784 (6198)	146640 (13949)
**Reflections used for R-free**	2002 (196)	5996 (498)	3369 (275)	3127 (317)	7403 (694)
**R-work**	0.2342 (0.3481)	0.2152 (0.2643)	0.2253 (0.4728)	0.2156 (0.3273)	0.2357 (0.3859)
**R-free**	0.2338 (0.3513)	0.2641 (0.3502)	0.2595 (0.4347)	0.2456 (0.3435)	0.2525 (0.3976)
**CC(work)**	0.926 (0.712)	0.944 (0.884)	0.959 (0.616)	0.954 (0.875)	0.962 (0.649)
**CC(free)**	0.893 (0.661)	0.922 (0.743)	0.933 (0.662)	0.934 (0.867)	0.942 (0.580)
**Number of non-hydrogen atoms**	11800	26149	13225	13319	27369
**macromolecules**	11604	25574	12946	13040	26795
**ligands**	196	352	244	244	392
**Protein residues**	1708	3500	1748	1752	182
**RMS(bonds) (Å)**	0.01	0.006	0.015	0.013	3505
**RMS(angles) (º)**	1.25	0.86	1.6	1.67	0.010
**Ramachandran favored (%)**	94.27	96	95.32	95.6	1.59
**Ramachandran allowed (%)**	5.32	3.79	4.62	4.4	96.05
**Ramachandran outliers (%)**	0.41	0.2	0.06	0	0.26
**Rotamer outliers (%)**	0	0.74	0.3	0.44	2.46
**Clashscore**	10.54	9.66	7.98	9.31	6.54
**Average B-factor (Å2)**	112.29	48.17	79.36	73.03	39.76
**macromolecules**	112.47	48.34	79.59	73.2	39.59
**ligands**	101.71	41.1	70.6	66.47	44.24
**solvent**		39.69	53.81	57.83	54.88

Statistics for the highest-resolution shell are shown in parentheses

a

**mobile phase A**	H_2O + 0.5% formic acid
**mobile phase B**	MeOH + 0.5% formic acid
**column temperature**	55 °C
**injection volume**	2 µL
**autosampler temperature**	4 °C

	Time (min)	%B	Flow rate (µL/min)
Gradient	0.00	0	200
	2.50	0	200
	3.00	95	200
	5.00	95	200
	5.10	0	200
	10.00	0	200

b

Compound	Q1 (m/z, Da)	Q3 (m/z, Da)	Dwell time (ms)	DP (volts)	EP (volts)	CE (volts)	CXP (volts)	RT (min)
α-Ketoglutaric acid	145	101 / 56.9	100	-38	-10	-8	-4	1.97
2-Hydroxyglutaric acid	147	84.9	100	-50	-10	-20	-5	1.78
3-Phosphoglyceric acid	185	96.7	100	-40	-10	-20	-5	1.76
3-Phosphoserine	182.9	79.1	100	-47	-8	-20	-15	1.57

Supplementary Table 2. Targeted LC-MS method for quantitative measurements of serine synthesis pathway intermediates. a Liquid chromatography method parameters are shown in the two tables. The MS instrument parameters were set as follows: nebulizer gas, 60 psi; auxiliary gas, 75 psi; curtain gas, 30 psi; collision gas, medium; ion spray voltage, -4500 V and temperature, 550 °C; interface heater, 100°C. **b** Mass transitions of the target analytes and MS detection parameters. The MRM detection window was set to 30 sec. Q1, quadrupole 1 (mother ion m/z); Q3, quadrupole 3 (daughter ion m/z); DP, declustering potential; EP, entrance potential; CE, collision energy; CXP, collision cell exit potential; RT, retention time.

3. Supplementary References

1. Laskowski, R. A. & Swindells, M. B. LigPlot+: multiple ligand-protein interaction diagrams for drug discovery. *J. Chem. Inf. Model.* **51**, 2778–2786 (2011).

2. Zhou, H. et al. Toward a comprehensive characterization of a human cancer cell phosphoproteome. *J. Proteome Res.* **12**, 260-271 (2013).

3. Bian, Y. An enzyme assisted RP-RPLC approach for in-depth analysis of human liver phosphoproteome. *J. Proteomics*. **96**, 253–262 (2014).

4. Ochoa, D. et al. The functional landscape of the human phosphoproteome. *Nat. Biotechnol.* **38**, 365–373 (2020).

5. Zerbini, E. et al. Post-translational regulation of human D-3-phosphoglycerate dehydrogenase in Alzheimer's disease. *Protein Sci.* **35**, e70505 (2026).
