# Supplementary material for: Molecular mechanisms of transhydrogenase activity and allosteric regulation in eukaryotic type II PHGDH Ser33
Source: Nat Commun. 2026 Jun 19;17:7756. doi: 10.1038/s41467-026-74211-9 (PMC13434204; doi:10.1038/s41467-026-74211-9)
Supplement: Supplementary file 3 [file 41467_2026_74211_MOESM3_ESM.pdf]

nature portfolio

Peer Review File

Molecular mechanisms of transhydrogenase activity and allosteric regulation in eukaryotic Type II PHGDH Ser33.

Corresponding Author: Professor Dr. Marcelo E. Guerin

This file contains all reviewer reports in order by version, followed by all author rebuttals in order by version.

Attachments originally included by the reviewers as part of their assessment can be found at the end of this file.

Version 0:

Reviewer comments:

Reviewer #1

(Remarks to the Author)

[Editorial Note: See attachment at end of file]

Reviewer #2

(Remarks to the Author)
This manuscript provides novel and detailed insights into the structure and function of the Saccharomyces cerevisiae Ser33 enzyme, a type II PHGDH, highlighting its transhydrogenase activity and regulation via L-serine binding. The comprehensive combination of crystallography, mutagenesis, and enzymatic assays build a strong model.
I like very much the manuscript. In particular, I find appealing the flip-flap suggested molecular mechanism within the tetramer, the mechanism of inhibition by the N-terminal segment and the identification of the L-serine allosteric inhibitor binding sites.
Overall, this is an impressive highly informative manuscript that will be of interest to a wide audience in biochemistry, structural biology, and metabolic research. With minor revisions to enhance the depth of the analysis around allosteric regulation, it should extend the audience to those with interest in drug discovery and drug development directed to the allosteric mechanism in the human counterpart.
I have minor suggestions for improvement:
1- On the flip-flap suggested mechanism. The Fig7 representation is welcomed, but it can be improved. It is not easy to show dynamics in simple figures and schemes. However, I think that the attractiveness to the wide readership of the manuscript depends on the clarity of the schematic part of figures. I suggest that the authors work on improving the scheme-based models to explain the suggested mechanisms. Perhaps some arrows or step-by-step depiction will help to show the presumed dynamics and movement required on the protein and on the substrates.
2- In Fig. 7, What could be the meaning of the difference in the slope/shape of the temperature-stability curves? While the unliganded protein and the L-serine bound proteins share the shape and slope, other slopes/shapes diverge. Presumably, the binding site of the aKG is stabilized at its binding site but some distant regions are destabilized? Can the author comment on the shape of the curves and on which regions are destabilized by aKG binding?
3- It might be helpful to emphasize how the cofactor locking mechanism and the active site CXC motif interact or synergize. The figures are very well descriptive. Again, the inclusion of simplified schemes, with arrows may help to better visualize the relationships between the different domains, binding sites and allosteric effects.
4- The discovery and structural validation of the serine allosteric site is outstanding. However, given its allosteric role, we suggest the authors explore or discuss: a- Possible dynamic effects that translate the allosteric effect from the ACT domain interfaces to the catalytic core. b- Have the author attempted simple Molecular dynamics simulations in the presence and absence of L-serine, and with or without NADH? What clues on the allosteric effects emerge from simple MD simulations?
5- The L-serine allosteric inhibition is conserved. On the other hand, the pharmaceutical inhibition (or allosteric inhibition) of the human counterpart is considered for drug therapies against cancers. Therefore, for the benefit of wide readership of the article, I suggest that the authors invest some effort in depicting the possible similarities or differences with the human counterpart that is considered as a drug target. Is the L-serine binding site conserved?. In the human structure, is it possible

to identify the L-serine binding site in the human drug target counterpart? Would a figure that highlights the alignment of the L-serine binding site among different PHGDH types, help to emphasize the conservation/divergence of regulatory mechanism?.
6- The discussion section and the Figure 7 could be extended to more clearly depict, using simplified schemes and arrows, the regulation by the N-terminal region and the L-serine allosteric inhibition.
7- Additional information on the Supplementary figure legends would be beneficial. Explain the seemed repetition of the image in Supplementary Figure 2. Double-check stated PDB-codes.

Reviewer #3

(Remarks to the Author)
The paper from Perrone et al. focuses on the mechanisms of Ser33, an enzyme involved in de novo biosynthesis of L-serine in the yeast Saccharomyces cerevisiae. The authors did a deep structural characterization using X-ray crystallography, LC-MS/MS, mutagenesis and biochemical studies to deep into specific functional details on the classical 3PG dehydrogenation as well as on the transhydrogenase reaction based on a-ketoglutarate.
This is a very detailed work, who addressed the scientific problem in great depth. The study is of significance to the field and represents a significant step forward, providing significant details to the reaction mechanism.

1. The main criticism is about the tentative made by the authors to link this study to the role of human PHGDH and related pathological states (a very relevant issue). Ser33 is a type II PHGDH, containing a catalytic core and the ACT domain, while lacking the ASB one present in type I PHGDH, such as the human counterpart. The modulation of human PHGDH is peculiar and can be investigated only using this protein, especially now that its participation in the serinosome has been reported. Unfortunately, the 3D structure of the whole-length protein has not been solved yet. For sake of clarity, the mention to human PHGDH role (and the correlation with cancer and neurodegenerative conditions) in the Abstract, Introduction and Discussion sections should be amended.
In addition, the sequence identity score between Ser33 and human PHGDH should be reported.

2. I suggest to improve the slightly verbose (although detailed) presentation of the experimental data, that does not help the general reader. I trust that the manuscript would benefit from a shortening and text revision.

3. “The overall architecture of Ser33” and “The transhydrogenase mechanism of Ser33” paragraphs: is it possible to measure the dissociation constant for the NAD+ and NADH cofactors?

4. “Discussion” section: here, when the interaction with PSAT and PSP is mentioned, a discussion about the proposed serinosome should be introduced.
Furthermore, I’m lost where the flip-flop mechanism for the Ser33 tetramer has been proved.

Further points:
- please modify mutant with variant when referring to protein
- Introduce in Suppl Figure 1b a calibration curve, change gel-filtration with gel permeation

Version 1:

Reviewer comments:

Reviewer #1

(Remarks to the Author)
The authors have addressed the previously raised concerns and I believe the manuscript if published will be a good article with broad interest.

Reviewer #2

(Remarks to the Author)
The revised manuscript addresses the points raised in my previous review. The authors have improved the presentation of the data and clarified several aspects of the structural analysis.
The new molecular dynamics simulations provide a useful dynamic perspective that helps explain the structural observations and the proposed communication between protomers.
The additional kinetic measurements and the evolutionary analysis of the N-terminal extension also strengthen the mechanistic interpretation of the work.
Overall, the revisions improve the manuscript and I support its publication.

Reviewer #3

(Remarks to the Author)
The revised version of the paper from Perrone et al. represents a significant improvement compared to the original one.
I’ve to mention that not all the criticisms and additional experiments I suggested were taken into consideration. In particular,

when phosphorylation is mentioned, the recent paper on the human enzyme (under physiological and non-pathological conditions) should be considered DOI: 10.1002/pro.70505.
Furthermore, the text is still quite verbose. Anyway, I agree with my previous view that this is a very detailed work, who addressed the scientific problem in great depth.
The study is of significance to the field and represents a significant step forward, providing significant details to the reaction mechanism.

Open Access This Peer Review File is licensed under a Creative Commons Attribution 4.0 International License, which permits use, sharing, adaptation, distribution and reproduction in any medium or format, as long as you give appropriate credit to the original author(s) and the source, provide a link to the Creative Commons license, and indicate if changes were made.
In cases where reviewers are anonymous, credit should be given to 'Anonymous Referee' and the source.
The images or other third party material in this Peer Review File are included in the article's Creative Commons license, unless indicated otherwise in a credit line to the material. If material is not included in the article's Creative Commons license and your intended use is not permitted by statutory regulation or exceeds the permitted use, you will need to obtain permission directly from the copyright holder.
To view a copy of this license, visit https://creativecommons.org/licenses/by/4.0/

Point-by-point response to reviewers' comments

Reviewer #1:

The manuscript titled "Molecular mechanisms of transhydrogenase activity and allosteric regulation in Ser33, a key enzyme in de novo L-serine biosynthesis" by Perrone et al. presents solid structural work with S. cerevisiae PHGDH enzyme characterized in multiple states, bound to its physiologically relevant ligands, and probed through point mutations. But the manuscript suffers greatly from its presentation, which needs to be improved before it can be published in Nature Comm.

ANSWER: We thank the reviewer for all the constructive comments and suggestions to improve the manuscript.

1. The work talks about mechanistic details of the enzyme activity in terms of the structural features, but gives very superficial background information or material for comparison. That makes it hard to gage the novelty and the relevance of the work presented. Based on only the manuscript, it looks almost like nothing is known about these enzymes. However, search by Ser33 sequence of the pdb database returns over 200 entries, spanning eukaryotes, prokaryotes and archaea, with relevant ligands (including Ser), and of course papers discussing their mechanisms associated with them… A lot of them are referenced, but in a very condensed, not informative way. Usually, the readers are not required to go and first read the whole of the referenced works in order to understand the manuscript contribution.

ANSWER: When searching the PDB database with the Ser33 amino acid sequence (UniProt ID P40510), 218 entries are retrieved. Screening through this list, we note that 42 of the 218 entries are phosphoglycerate dehydrogenases (PHGDH), while the remaining 176 entries correspond to other enzymes —mostly dehydrogenases acting on other organic acids including glycerate, D-lactate, formate, 4-phosphoerythronate, phosphite, D-mandelate. Five of the 42 PHGDH entries are the ones contributed by our study. The remaining 37 PHGDH entries are linked to 14 publications, of which 7 are cited in our original manuscript. Six of the remaining 7 publications, which are not cited in our original manuscript, describe structural work on type I PHGDHs.

Focusing on these 37 PHGDH PDB entries, they show that crystal structures are available for bacterial and eukaryotic enzymes, encompassing the three PHGDH classes: (i) type I PHGDHs, structures have been described for the enzymes of *Mycobacterium tuberculosis*, maize and human —however, no full-length structures are available for eukaryotic type I PHGDHs; (ii) type II PHGDHs, the *Escherichia coli* enzyme has by far been studied most intensively from a structural point of view; (iii) type IIIK PHGDHs, structures have been described for the *Entamoeba Histolytica* enzyme. Collectively, these studies established the conserved Rossmann-fold NAD-binding domain shared by all PHGDHs, delineated class-specific domain architectures, and revealed/predicted distinct modes of quaternary assembly and regulation. We have made an effort to improve the readability of the entire manuscript, incorporating additional information on PHGDHs and references as requested.

1. Schuller, D. et al. The allosteric ligand site in the V_{max}-type cooperative enzyme phosphoglycerate dehydrogenase. *Nat. Struct. Mol. Biol.* 2, 69–76 (1995). doi: 10.1038/nsb0195-69 (*E. coli* type II PHGDH; cited in original manuscript; reference 24).

2. Bell, J. K. et al. Multiconformational states in phosphoglycerate dehydrogenase. *Biochemistry* 43, 3450–3458 (2004). doi: 10.1021/bi035462e. (*E. coli* type II PHGDH; cited in original manuscript; reference 26).

3. Dey, S. et al. Crystal structure of *Mycobacterium tuberculosis* D-3-phosphoglycerate dehydrogenase: extreme asymmetry in a tetramer of identical subunits. *J. Biol. Chem.* 280, 14892–14899 (2005). doi: 10.1074/jbc.M414489200. (*M. tuberculosis* type I PHGDH; cited in original manuscript; reference 28).

4. Grant, G. A. et al. Identification of amino acid residues contributing to the mechanism of cooperativity in *Escherichia coli* D-3-phosphoglycerate dehydrogenase. *Biochemistry* 44, 16844–16852 (2005). doi: 10.1021/bi051681j. (*E. coli* type II PHGDH; not cited in original manuscript; now cited).

5. Dey, S. et al. The effect of hinge mutations on effector binding and domain rotation in *Escherichia coli* D-3-phosphoglycerate dehydrogenase. *J. Biol. Chem.* 282, 18418–18426 (2007). doi: 10.1074/jbc.M701174200. (*E. coli* type II PHGDH; cited in original manuscript; reference 32).

6. Dey, S. et al. Structural analysis of substrate and effector binding in *Mycobacterium tuberculosis* D-3-phosphoglycerate dehydrogenase. *Biochemistry* 47, 8271–8282 (2008). doi: 10.1021/bi800212b. (*M. tuberculosis* type I PHGDH; cited in original manuscript; reference 58).

7. Gao, D. et al. Discovery of novel drug-like PHGDH inhibitors to disrupt serine biosynthesis for cancer therapy. *J. Med. Chem.* 66, 285–305 (2023). doi: 10.1021/acs.jmedchem.2c01202. (human type I PHGDH; not cited in original manuscript; now cited).

8. Unterlass, J. E. et al. Validating and enabling phosphoglycerate dehydrogenase (PHGDH) as a target for fragment-based drug discovery in PHGDH-amplified breast cancer. *Oncotarget* 9, 13139–13153 (2016). doi: 10.18632/oncotarget.11487. (human type I PHGDH; not cited in original manuscript; now cited).

9. Unterlass, J. E. et al. Structural insights into the enzymatic activity and potential substrate promiscuity of human 3-phosphoglycerate dehydrogenase (PHGDH). *Oncotarget* 8, 104478–104491 (2017). doi: 10.18632/oncotarget.22327. (human type I PHGDH; cited in original manuscript; reference 13).

10. Weinstabl, H. et al. Intracellular trapping of the selective phosphoglycerate dehydrogenase (PHGDH) Inhibitor BI-4924 disrupts serine biosynthesis. *J. Med. Chem.* 62, 7976–7997 (2019). doi: 10.1021/acs.jmedchem.9b00718. (human type I PHGDH; not cited in original manuscript; now cited).

11. Mullarky, E. et al. Inhibition of 3-phosphoglycerate dehydrogenase (PHGDH) by indole amides abrogates de novo serine synthesis in cancer cells. *Bioorg. Med. Chem. Lett.* 29, 2503–2510 (2019). doi: 10.1016/j.bmcl.2019.07.011. (human type I PHGDH; not cited in original manuscript; now cited).

12. Tan, Y. et al. Biophysical and biochemical properties of PHGDH revealed by studies on PHGDH inhibitors. *Cell. Mol. Life Sci.* 79, 27 (2021). doi: 10.1007/s00018-021-04022-2. (human type I PHGDH; not cited in original manuscript; now cited).

13. Zhang, Y. et al. Phosphoglycerate dehydrogenase is required for kernel development and defines a predominant serine synthesis pathway in maize. *Plant Cell.* 37, koaf126 (2025). doi: 10.1093/plcell/koaf126. (maize type I PHGDH; not cited in original manuscript; now cited).

14. Singh, R. K. et al. Crystal structures and kinetics of Type III 3-phosphoglycerate dehydrogenase reveal catalysis by lysine. *FEBS J.* 281, 5498–5512 (2014). doi: 10.1111/febs.13091. (E. histolytica type IIIK PHGDH; cited in original manuscript, reference 23).

2. Excessive descriptions of the structural interactions take most of the space in the manuscript, with barely any given to explain the rationale, relevance (see 1) or authors' thought process.

ANSWER: We find it useful that selected residues and structural interactions are described, discussed, and incorporated in the Results and Discussion sections of the main text.

A lot of this can go into contact list tables (if it's a long list for an interface), into Suppl., or be better shown on the Figures.

ANSWER: We have now incorporated schematic two-dimensional representations of the Ser33-ligand complexes illustrating their interactions, included as the new Supplementary Figures 4 to 8.

The Figures right now have residues labeled, but are not very helpful because too crowded: mutated resides not distinguished (highlighted somehow), some are not even visible, like C128; contacts are not shown by lines - makes it hard to perceive angles and distance in 2D view. I would recommend for Figure 2, panels on the right of the lower group - dedicate to contacts: loose the ribbons, only show relevant/discussed in the text contacts, display lines and distances.

ANSWER: We believe that the combination of Figure 2, panels (i) e/f, (ii) g/h, and (iii) i/j, provides a clear visualization of the active sites in each of the Ser33-NADH-PGA (PDB code 8PIR), Ser33-NADH-PHP (PDB code 8PIO), and Ser33-NADH-2HG-SER (PDB code 8Q2I) complexes, respectively. These panels are now complemented with the two-dimensional representations of the Ser33-ligand complexes included as the new Supplementary Figures 4 to 8. We found appropriate to incorporate the label of C128 in Figure 4, panels c, d, e, and f. Finally, it is worth mentioning that the location of the mutated residues was already described in the original Supplementary Figure 7. We have made an effort to modify this Figure —now Supplementary Figure 12— to improve the visualization/localization of the selected mutated residues.

Figure 5 - Ser (which is the very point of this figure) is shown as a yellow blob on most of the panels (happily blending in with other yellow-orange parts), and only in the middle circle is the attempt to show the interactions - but it is impossible to see through with no lines for them (ribbon is not needed here!), and it does not help that NAD is colored the same as the ACT and so on.

ANSWER: In Figure 5, panels a, b, c, d and f, NADH, 2HG and serine ligands are shown not only using a surface representation but also labeled. We have modified the color of serine to green for clarity.

3. The mutational studies are hard to appreciate because of poorly explained rationale (see 2) and because only enzymatic activity data is not sufficient to build the mechanistic explanations upon. Especially since the assay setup involves a coupled step that shares product/substrate with the measured reaction. One needs binding/affinity measurement for the relevant ligands with mutated versions of the protein to be able to draw conclusions.

ANSWER: As clearly explained in the manuscript, and illustrated in previous Supplementary Figure 7 —now Supplementary Figure 12—, the mutated residues were carefully selected based on: (i) previously known roles in the catalytic site —His347, Glu316—, (ii) evolutionary conservation in type II PHGDHs specifically —Cys128 and Cys130 in the CXC motif, Phe153 and His208 in the NBD—, (iii) location in a unique N-terminal extension —50 amino acids— whose function we wanted to investigate (Δ1-50, Arg41), and (iv) proximity to the regulator serine in the ACT domain —His404, Asn406, Asn422.

There is a thermal stability experiment data in the paper (which for some reason is not even part of the Results, and only appear in Discussion), but it is only done with the wt protein.

ANSWER: We have moved the thermal stability experiment data to the Results section.

4. Overall, the work is presented without using the wealth of data in pdb database, sequencing databases and (co)-folding softwares like AlphaFold, Boltz, Chai to figure out the impact of mutations. The authors can do better in taking advantage of all these things that would enrich this work, and also make it into better understandable and appreciable article for the readers.

ANSWER: We found it very interesting to perform and provide the structural comparison between Ser33 and human PHGDH —also based on comments and suggestions of reviewers 2 and 3. In the new version of the manuscript, we have incorporated these analyses in the Discussion section, and prepared a new Supplementary Figure 17, accordingly.

5. N-terminal Extension: The role of the unique N-terminal extension in modulating enzyme activity is not clear. However, the discussion would benefit from a more detailed analysis of its evolutionary conservation and potential regulation via phosphorylation.

ANSWER: We performed an in-depth phylogenetic analysis to elucidate the evolutionary origin and conservation of the N-terminal extension in Type II PHGDHs, as well as the conservation of the putative phosphorylation sites in this N-terminal extension. Our analyses show that the N-terminal extension is absent from prokaryotic PHGDHs, but conserved in all fungal lineages, where some of the identified phosphorylation sites are also widely conserved, strongly supporting a regulatory role. In the new version of the manuscript, we have incorporated these analyses in the Results, Discussion and Methods sections, and prepared new Figures —Figure 8 and Supplementary Fig. 16—, accordingly.

The authors mention several putative phosphorylation sites; providing experimental evidence (or a more thorough bioinformatics analysis) to support their regulatory role would further strengthen this aspect.

ANSWER: We agree with the reviewer that further advancing in the elucidation of the regulatory role of the putative phosphorylation sites, by providing experimental evidence, would be relevant for the field. However, considering the complexity of the task, it certainly represents a separate several-year project. We believe that this is out of the scope of the current manuscript.

6. Although the manuscript provides activity measurements under several conditions, additional kinetic parameters (e.g., Km and Vmax for both the transhydrogenase and α-KG reductase reactions) would enable a more quantitative comparison of wild-type and mutant enzymes. If these data are available in supplementary materials, the authors should reference them explicitly.

ANSWER: Based on our initial activity measurements, we observed markedly increased enzymatic activities for Ser33 mutants lacking the N-terminal extension (Δ1–50) or carrying point mutations in the serine-binding site. Notably, for these mutants, the transhydrogenase activity became independent of externally supplied α-ketoglutarate, suggesting an increased affinity for this substrate. To quantitatively test this hypothesis, we determined the kinetic parameters of the α-ketoglutarate reductase activity for the Δ1–50 and His404Asn mutants and compared them to wild-type Ser33. These spectrophotometric assays revealed a 9-fold and 4-fold increase in Vmax for the Δ1–50 and His404Asn mutants, respectively, together with a 2.4-fold and 4.7-fold decrease in Km for α-ketoglutarate. These results are consistent with our mechanistic interpretation and corroborate the increased apparent affinity for α-ketoglutarate in these variants. The corresponding data have now been incorporated into the Results section and Supplementary Figure 14 of the revised manuscript. It is worth noting that detailed kinetic analyses of the transhydrogenase activity are technically challenging. Detection of this enzymatic activity requires coupling to another enzyme —PSAT1— which regenerates one of the substrates of the transhydrogenase activity —α-ketoglutarate. In addition, the transhydrogenase activity is measured as an end-point assay based on LC-MS quantification of reaction products after fixed incubation times. Under these conditions, reliable determination of kinetic parameters is not straightforward and would require substantial additional methodological development. We therefore consider such analyses to be beyond the scope of the present study.

7. Explain solid circle and empty circle in Fig 1.

ANSWER: The solid circle corresponds to a 2-fold axis. We have incorporated this information in the Figure legend. We have removed the empty circles in all figures for clarity.

8. Show the statistics significance in Fig. 3.

ANSWER: Statistical significance is now shown in Figure 3 for the differences between Ser33 variant activities compared to wild-type activity.

9. Label axes in Supplementary Fig. 6 (LC-MS chromatograms).

ANSWER: We have labelled the axes as requested —now Supplementary Figure 10.

10. Please include recent publication: https://www.nature.com/articles/s41586-022-04758-2. I think it is relevant to current study.

ANSWER: We agree with the reviewer that this publication is relevant to our study, and for this reason, we had already cited it in our originally submitted manuscript —reference 73— now reference 74.

Reviewer #2:

This manuscript provides novel and detailed insights into the structure and function of the Saccharomyces cerevisiae Ser33 enzyme, a type II PHGDH, highlighting its transhydrogenase activity and regulation via L-serine binding. The comprehensive combination of crystallography, mutagenesis, and enzymatic assays build a strong model. I like very much the manuscript. In particular, I find appealing the flip-flap suggested molecular mechanism within the tetramer, the mechanism of inhibition by the N-terminal segment and the identification of the L-serine allosteric inhibitor binding sites. Overall, this is an impressive highly informative manuscript that will be of interest to a wide audience in biochemistry, structural biology, and metabolic research. With minor revisions to enhance the depth of the analysis around allosteric regulation, it should extend the audience to those with interest in drug discovery and drug development directed to the allosteric mechanism in the human counterpart. I have minor suggestions for improvement:

ANSWER: We thank the reviewer for considering our manuscript to be impressive and highly informative, and of interest to a wide audience in biochemistry, structural biology, and metabolic research, and for all the constructive comments and suggestions to improve the manuscript.

1- On the flip-flap suggested mechanism. The Fig7 representation is welcomed, but it can be improved. It is not easy to show dynamics in simple figures and schemes. However, I think that the attractiveness to the wide readership of the manuscript depends on the clarity of the schematic part of figures. I suggest that the authors work on improving the

scheme-based models to explain the suggested mechanisms. Perhaps some arrows or step-by-step depiction will help to show the presumed dynamics and movement required on the protein and on the substrates.

ANSWER: Many thanks for pointing this out to us. In the new version of the manuscript, we have developed a new version of Figure 7 —now Figure 9—, accordingly.

2- In Fig. 7, What could be the meaning of the difference in the slope/shape of the temperature-stability curves? While the unliganded protein and the L-serine bound proteins share the shape and slope, other slopes/shapes diverge. Presumably, the binding site of the aKG is stabilized at its binding site but some distant regions are destabilized? Can the author comment on the shape of the curves and on which regions are destabilized by aKG binding?

ANSWER: The slope is associated to the degree of cooperativity in the unfolding (steep slope/sharp transition/high cooperative unfolding; shallow slope/broad transition/less cooperative unfolding). We prefer to be circumspect in our analysis of the slopes, since there is no clear reason for the differences in the slope/shape of the curves for the (i) aKG and PHP, compared to the (ii) unliganded, NADH-bound, and L-serine-bound Ser33 forms.

3- It might be helpful to emphasize how the cofactor locking mechanism and the active site CXC motif interact or synergize. The figures are very well descriptive. Again, the inclusion of simplified schemes, with arrows may help to better visualize the relationships between the different domains, binding sites and allosteric effects.

ANSWER: Many thanks for pointing out that figures are very well descriptive. We have incorporated the label of Cys128 in Figure 4, panels c, d, e and f, for clarity. In addition, we have expanded and further developed the explanations on the proposed mechanism in the Discussion section.

4- The discovery and structural validation of the serine allosteric site is outstanding. However, given its allosteric role, we suggest the authors explore or discuss: a- Possible dynamic effects that translate the allosteric effect from the ACT domain interfaces to the catalytic core. b- Have the author attempted simple Molecular dynamics simulations in the presence and absence of L-serine, and with or without NADH? What clues on the allosteric effects emerge from simple MD simulations?

ANSWER: Following the reviewer's suggestion, we have performed MD simulations to study the regulatory mechanism of Ser33, associated to serine binding. The corresponding data have been incorporated into the Results, Discussion and Methods sections, and new Figure 6 of the revised manuscript.

5- The L-serine allosteric inhibition is conserved. On the other hand, the pharmaceutical inhibition (or allosteric inhibition) of the human counterpart is considered for drug therapies against cancers. Therefore, for the benefit of wide readership of the article, I suggest that the authors invest some effort in depicting the possible similarities or differences with the human counterpart that is considered as a drug target. Is the L-serine binding site conserved? In the human structure, is it possible to identify the L-serine binding site in the human drug target counterpart? Would a figure that highlights the alignment of the L-serine binding site among different PHGDH types, help to emphasize the conservation/divergence of regulatory mechanism?

ANSWER: We agree with the reviewer that depicting similarities and differences between Ser33 and the human counterpart is very relevant. In the Introduction section, we review the main known structural and functional differences between type I (including human), type II (including Ser33), and type III PHGDHs. We also point out that, while serine allosterically inhibits type II PHGDHs, the mammalian PHGDHs that have been studied so far, are not sensitive to serine. We have performed a detailed comparison and analysis between the experimental structures of Ser33 —this study— and human PHGDH. It is worth mentioning that, as reviewer 3 correctly points out, the three-dimensional structure of full-length human PHGDH has not been reported yet.

6- The discussion section and the Figure 7 could be extended to more clearly depict, using simplified schemes and arrows, the regulation by the N-terminal region and the L-serine allosteric inhibition.

ANSWER: In the revised manuscript, we have developed a new version of Figure 7 - now divided into new Figures 6 and 9, taking this feedback into account.

7- Additional information on the Supplementary figure legends would be beneficial.

ANSWER: We have made an effort (i) to incorporate additional information in Supplementary Figure legends and (ii) to improve the readability of the entire manuscript.

Explain the seemed repetition of the image in Supplementary Figure 2.

ANSWER: Many thanks for pointing this out to us. The images correspond to stereo views. We have incorporated this information in the corresponding Supplementary Figure legend.

Double-check stated PDB-codes.

ANSWER: We have revised/confirmed/homogenized the PDB codes over the main text and Supplemental Information.

__Reviewer #3:__

The paper from Perrone et al. focuses on the mechanisms of Ser33, an enzyme involved in de novo biosynthesis of L-serine in the yeast Saccharomyces cerevisiae. The authors did a deep structural characterization using X-ray crystallography, LC-MS/MS, mutagenesis and biochemical studies to deep into specific functional details on the classical 3PG dehydrogenation as well as on the transhydrogenase reaction based on a-ketoglutarate. This is a very detailed work, who addressed the scientific problem in great depth. The study is of significance to the field and represents a significant step forward, providing significant details to the reaction mechanism.

ANSWER: We thank the reviewer for considering that our study is of significance to the field and represents a significant step forward, providing significant details to the reaction mechanism, all the constructive comments and suggestions in order to improve the manuscript.

1. The main criticism is about the tentative made by the authors to link this study to the role of human PHGDH and related pathological states (a very relevant issue). Ser33 is a type II PHGDH, containing a catalytic core and the ACT domain, while lacking the ASB one present in type I PHGDH, such as the human counterpart. The modulation of human PHGDH is peculiar and can be investigated only using this protein, especially now that its participation in the serinosome has been reported. Unfortunately, the 3D structure of the whole-length protein has not been solved yet. For sake of clarity, the mention to human PHGDH role (and the correlation with cancer and neurodegenerative conditions) in the Abstract, Introduction and Discussion sections should be amended. In addition, the sequence identity score between Ser33 and human PHGDH should be reported.

ANSWER: Many thanks for pointing out this to us. We have reorganized all information related to human PHGDH throughout the entire manuscript to improve clarity and prevent any potential overinterpretation. Given the importance of human PHGDH in human disease, including cancer and neurodegenerative disorders, we believe it is appropriate —as also suggested by reviewer 2— to compare these enzymes using the structural data currently available. In that context, we performed a detailed yet deliberately circumspect structural comparison and analysis between the experimental structures of eukaryotic Ser33 —this study— and the previously reported structures of truncated human PHGDH —also reporting the sequence identity score between Ser33 and human PHGDH— 31.17% identity —and incorporated our vision in the Discussion section, and Supplementary Figure 17.

2. I suggest to improve the slightly verbose (although detailed) presentation of the experimental data, that does not help the general reader. I trust that the manuscript would benefit from a shortening and text revision.

ANSWER: We have made an effort to improve the readability of the entire manuscript. However, we prefer to preserve the organization of the manuscript in its current form.

3. “The overall architecture of Ser33” and “The transhydrogenase mechanism of Ser33” paragraphs: is it possible to measure the dissociation constant for the NAD+ and NADH cofactors?

ANSWER: We thank the reviewer for this thoughtful suggestion. Although this experiment would certainly strengthen the study, it could not be performed within the framework of the current work.

4. “Discussion” section: here, when the interaction with PSAT and PSP is mentioned, a discussion about the proposed serinosome should be introduced.

ANSWER: We agree with the reviewer. We have incorporated this notion in the Discussion section (second-to-last paragraph) and included a reference accordingly.

Rabattoni, V., Marchesani, F., Murtas, G., Sacchi, S., Mozzarelli, A., Bruno, S., Peracchi, A., Pollegioni, L., Campanini, B. The human phosphorylated pathway: a multienzyme metabolic assembly for l-serine biosynthesis. *FEBS J.* 290, 3877-3895 (2023). doi: 10.1111/febs.16787.

Furthermore, I’m lost where the flip-flop mechanism for the Ser33 tetramer has been proved.

ANSWER: We agree with the reviewer. We have tone down the statement. A flip-flop mechanistic model is proposed for Ser33 in the Discussion section. We have revised the corresponding text for better clarity.

5. please modify mutant with variant when referring to protein.

ANSWER: The text has been revised accordingly.

6. Introduce in Suppl Figure 1b a calibration curve, change gel-filtration with gel permeation

ANSWER: A calibration curve has been added to Supplementary Figure 1b and we have replaced ‘gel-filtration’ by the more general term ‘size-exclusion chromatography (SEC)’ throughout the manuscript.

We wish to take this opportunity to thank the reviewers for their many thoughtful suggestions, which have substantially improved the manuscript.

Reviewer #1

The authors have addressed the previously raised concerns and I believe the manuscript if published will be a good article with broad interest.

ANSWER: Many thanks for considering our revisions and your positive feedback on the revised version of the manuscript.

Reviewer #2

The revised manuscript addresses the points raised in my previous review. The authors have improved the presentation of the data and clarified several aspects of the structural analysis. The new molecular dynamics simulations provide a useful dynamic perspective that helps explain the structural observations and the proposed communication between protomers. The additional kinetic measurements and the evolutionary analysis of the N-terminal extension also strengthen the mechanistic interpretation of the work. Overall, the revisions improve the manuscript and I support its publication.

ANSWER: Many thanks for considering our revisions and your positive feedback on the revised version of the manuscript.

Reviewer #3

The revised version of the paper from Perrone et al. represents a significant improvement compared to the original one. I've to mention that not all the criticisms and additional experiments I suggested were taken into consideration. In particular, when phosphorylation is mentioned, the recent paper on the human enzyme (under physiological and non-pathological conditions) should be considered DOI: 10.1002/pro.70505. Furthermore, the text is still quite verbose. Anyway, I agree with my previous view that this is a very detailed work, who addressed the scientific problem in great depth. The study is of significance to the field and represents a significant step forward, providing significant details to the reaction mechanism.

ANSWER: Many thanks for considering our revisions and your positive feedback on the revised version of the manuscript. In addition, we have incorporated the new reference - doi:10.1002/pro.70505 - in the main text and Supplementary Information, as requested.

We wish to take this opportunity to thank the referees for their many thoughtful suggestions that have made the manuscript so much better.

The manuscript titled "Molecular mechanisms of transhydrogenase activity and allosteric regulation in Ser33, a key enzyme in de novo L-serine biosynthesis" by Perrone et al. presents solid structural work with S. cerevisiae PHGDH enzyme characterized in multiple states, bound to its physiologically relevant ligands, and probed through point mutations. But the manuscript suffers greatly from its presentation, which needs to be improved before it can be published in Nature Comm.

Major points:

1. The work talks about mechanistic details of the enzyme activity in terms of the structural features, but gives very superficial background information or material for comparison. That makes it hard to gage the novelty and the relevance of the work presented. Based on only the manuscript, it looks almost like nothing is known about these enzymes. However, search by Ser33 sequence of the pdb database returns over 200 entries, spanning eukaryotes, prokaryotes and archaea, with relevant ligands (including Ser), and of course papers discussing their mechanisms associated with them… A lot of them are referenced, but in a very condensed, not informative way. Usually, the readers are not required to go and first read the whole of the referenced works in order to understand the manuscript contribution.

2. Excessive descriptions of the structural interactions take most of the space in the manuscript, with barely any given to explain the rationale, relevance (see 1) or authors' thought process. A lot of this can go into contact list tables (if it's a long list for an interface), into Suppl., or be better shown on the Figures. The Figures right now have residues labeled, but are not very helpful because too crowded: mutated resides not distinguished (highlighted somehow), some are not even visible, like C128; contacts are not shown by lines – makes it hard to perceive angles and distance in 2D view. I would recommend for Figure 2, panels on the right of the lower group – dedicate to contacts: loose the ribbons, only show relevant/discussed in the text contacts, display lines and distances. Figure 5 – Ser (which is the very point of this figure) is shown as a yellow blob on most of the panels (happily blending in with other yellow-orange parts), and only in the middle circle is the attempt to show the interactions – but it is impossible to see through with no lines for them (ribbon is not needed here!), and it does not help that NAD is colored the same as the ACT and so on.

3. The mutational studies are hard to appreciate because of poorly explained rationale (see 2) and because only enzymatic activity data is not sufficient to build the mechanistic explanations upon. Especially since the assay setup involves a coupled step that shares product/substrate with the measured reaction. One needs binding/affinity measurement for the relevant ligands with mutated versions of the protein to be able to draw conclusions. There is a thermal stability experiment data in the paper (which for some reason is not even part of the Results, and only appear in Discussion), but it is only done with the wt protein.

4. Overall, the work is presented without using the wealth of data in pdb database, sequencing databases and (co)-folding softwares like AlphaFold, Boltz, Chai to figure out the impact of mutations. The authors can do better in taking advantage of all these things that would enrich this work, and also make it into better understandable and appreciable article for the readers.

5. N-terminal Extension: – The role of the unique N-terminal extension in modulating enzyme activity is not clear. However, the discussion would benefit from a more detailed analysis of its evolutionary conservation and potential regulation via phosphorylation. The authors mention several putative phosphorylation sites; providing experimental evidence (or a more thorough bioinformatics analysis) to support their regulatory role would further strengthen this aspect.

6. Although the manuscript provides activity measurements under several conditions, additional kinetic parameters (e.g., Km and Vmax for both the transhydrogenase and α-KG reductase reactions) would

enable a more quantitative comparison of wild-type and mutant enzymes. If these data are available in supplementary materials, the authors should reference them explicitly.

Some minor issues:

1. Explain solid circle and empty circle in Fig 1.

2. Show the statistics significance in Fig. 3.

3. Label axes in Supplementary Fig. 6 (LC-MS chromatograms).

4. Please include recent publication: https://www.nature.com/articles/s41586-022-04758-2. I think it is relevant to current study.
